# 3D triply periodic minimal surface gyroid hydrogel scaffolds for soft tissue engineering

**DOI:** 10.1038/s41467-026-73452-y

**Published:** 2026-05-19

**Authors:** Xiaoxiao Han, Ning He, Dandan Yuan, Na Li, Wei Zhu, Xiaolong Zhu, Jing Li, Xun Yuan, Wenxin Wang, Xingshi Gu, Feng Chen, Wei Wang

**Affiliations:** 1 College of Mechanical and Vehicle Engineering, Hunan University, Changsha, China; 2 Department of Radiology, Xiangya 3rd Hospital, Central South University, Changsha, China; 3 School of Biomedical Engineering, Shenzhen Campus of Sun Yat-sen University, Guangming District, Shenzhen, Guangdong China

**Keywords:** Biomedical engineering, Implants, Biomaterials, Tissue engineering

## Abstract

Engineering functional soft tissue constructs remains difficult because scaffolds must meet the mechanical, physicochemical, and biological requirements simultaneously. Here, we present a cell-laden hydrogel gyroid scaffold designed to support vascularisation, long-term multicellular culture, and implantation for the development of 3D tissue constructs. The scaffold is structurally optimised to balance nutrient transport and mechanical stability, and is fabricated with high fidelity by mitigating cell-induced light scattering. The gyroid architecture supports the formation of dense microvascular networks throughout the 3D construct, and its curved Gaussian curvature promotes endothelial self-assembly. The scaffold also enables high-density co-culture of HepG2 cells and HUVECs without active perfusion, resulting in a vascularised 3D liver tumour model with enhanced tissue-specific function. Upon subcutaneous implantation in mice, the constructs show enhanced neovascularisation and facilitate tumour formation. These findings identify the gyroid scaffold as a biologically favourable architecture for generating bulk vascularised constructs, with potential applications in disease modelling, drug screening and regenerative medicine.

## Abstract (web summary)

Engineering functional soft tissue constructs remains difficult due to mechanical, physicochemical, and biological requirements. Here, the authors report on a gyroid scaffold design that promotes capillary network formation and enables production of vascularised 3D models and soft tissue engineering scaffolds.

## Introduction

Tissue engineering aims to fabricate vascularised and implantable tissues capable of integrating with the body and regenerating over time^1–3^. As a central component of these constructs, three-dimensional (3D) scaffolds provide structural support and a biomimetic microenvironment for cell growth, organisation, and tissue development, while also maintaining mechanical integrity upon implantation^4^. Advances in 3D bioprinting, particularly digital light processing (DLP), have enabled the fabrication of increasingly complex architectures at biologically relevant sizes (0.1–10 cm) and cell densities (≥1 × 10^7^ cells ml^−1^)^5–7^. As engineered tissues move towards larger and more complicated constructs, scaffold design has become a critical determinant of long-term cell viability and tissue performance. However, the scaffolds currently employed for cell-laden constructs, especially for softer models, are predominantly planar lattice-like^8–12^ or channelled^13,14^ architectures. These designs often fail to simultaneously satisfy mechanical (e.g. integrity and stability), physicochemical (e.g. porosity and interconnectivity) and biological (e.g. viability and vascularisation) requirements^15^. Therefore, new scaffolds offering greater flexibility, tunability and design freedom are urgently needed to meet these multifaceted demands.

Among the many challenges in engineering tissue constructs, vascularisation remains one of the most important^16–18^. Pre-vascularisation has emerged as a promising strategy to improve the in vivo performance of engineered constructs^7^. By establishing microvascular networks in vitro before implantation, pre-vascularised constructs can promote anastomosis with host vessels and reduce hypoxia-induced cell death. However, its effectiveness depends strongly on scaffold architecture and the ability to support uniform vascular network formation throughout the 3D construct. Existing strategies have mainly relied either on the fabrication of perfusable channels lined with endothelial cells (ECs)^19–21^ or on direct bioprinting of cell-laden bioinks to create constructs in which ECs self-assemble into capillary networks during culture^22,23^. However, channel-based approaches are often limited to relatively simple tubular networks with low vascular density due to fabrication constraints^17^, while cell-laden bioprinting strategies frequently employ relatively low cell densities^8,9^, produce thin-layered structures^10,24^, or suffer from compromised structural stability upon scaling^25^. These limitations highlight the need for scaffold architectures that can robustly support vascularisation within a fully 3D environment.

Designing soft-material scaffolds also requires balancing architectural complexity with structural stability. Features such as interconnected pores, distributed microchannels, and thin walls are beneficial for nutrient transport and cell activity^26^, but excessive porosity can weaken the scaffold and compromise its structural integrity. This challenge is amplified in low-concentration hydrogel bioinks, which often provide favourable biological compatibility but possess limited stiffness and mechanical strength after crosslinking^27,28^. A further difficulty lies in combining high cell density with reliable manufacturability. High cell density is beneficial for intercellular communication and tissue-specific function^29^, but in light-based bioprinting, cells can induce significant light scattering, which reduces fabrication resolution and fidelity and hinders the creation of fine structural features important for cell behaviour^5,30–32^. Addressing these concomitant issues is anticipated to yield substantial improvements in both in vitro and in vivo performance of engineered constructs. Furthermore, in addition to material-based physical cues such as stiffness, viscoelasticity, and plasticity^33^, recent studies suggest that cells also respond to 3D geometric cues, including curvature, through mechanotransduction-related pathways^34,35^. This raises the possibility of using scaffold geometry itself as a design variable to regulate cellular behaviour.

Triply periodic minimal surface (TPMS) scaffolds, such as gyroid, offer several features attractive for tissue engineering, including continuous curved surfaces, tunable porosity, high interconnectivity, and intrinsic structural stability^36^. TPMS-based scaffolds have been widely explored in bone and cartilage engineering, where they have been shown to support osteogenic and chondrogenic responses^37,38^. However, their application in cell-laden soft tissue engineering remains limited. The geometric complexity of TPMS architectures poses substantial fabrication challenges when combined with soft, cell-laden bioinks, which are typically dilute, swellable, and susceptible to light scattering. As a result, TPMS scaffolds have been used predominantly in hard tissue systems employing materials such as PLA, poly(ε-caprolactone), and hydroxyapatite, which offer higher stiffness and better processability^36^. Consequently, the potential of TPMS scaffolds for soft cellularised models, as well as the cellular responses they elicit within a 3D curved microenvironment, remains largely unexplored.

In this study, we investigated the potential of cell-laden gyroid scaffolds for the development of vascularised bulk soft tissue constructs. Specifically, we optimised the gyroid scaffold design to achieve suitable mass transport, mechanical compatibility, and curvature-related geometric cues for soft tissue applications. We then evaluated its ability to support capillary network formation under static culture and examined how the 3D curved architecture influenced cytoskeletal organisation, endothelial behaviour, and vascular morphogenesis. As a proof of concept, the scaffold was further applied to the construction of a vascularised 3D liver tumour model using HepG2 cells and ECs. Its biological performance was subsequently assessed in vivo following subcutaneous implantation.

## Results

### Optimal design of cell-laden gyroid scaffold ensuring adequate mass transport and mechanical strength

**Fig. 1:**
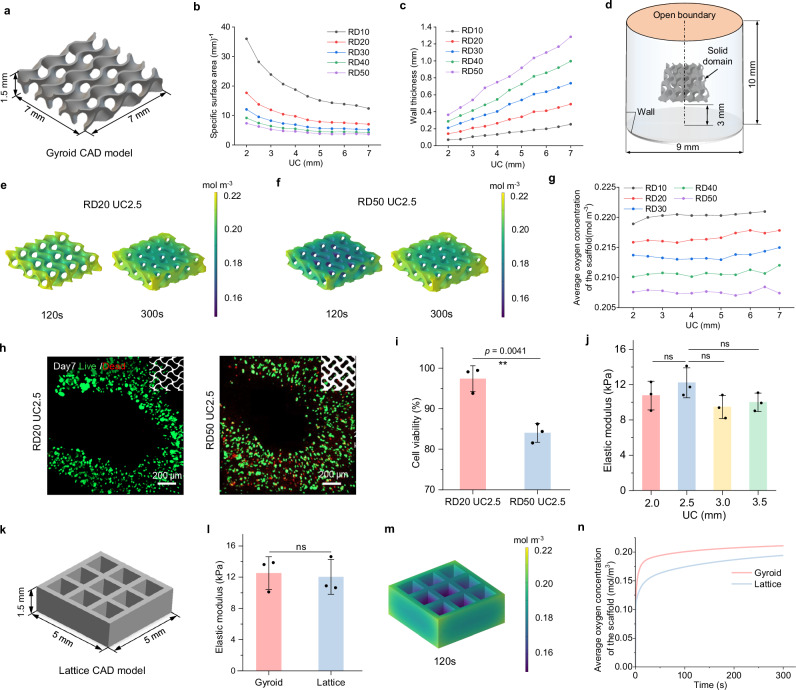
Optimisation of the gyroid scaffold to achieve adequate oxygen diffusion and mechanical stability. **a** CAD model of the gyroid scaffold. **b** Specific surface area and **c** space-averaged wall thickness of gyroid scaffolds with varying relative density (RD) and unit cell size (UC). **d** Schematic illustration of the computational domain used for modelling oxygen concentration within the scaffolds. Modelled spatiotemporal variations in oxygen concentration in two gyroid scaffolds: **e** RD = 20%, UC = 2.5 mm, and **f** RD = 50%, UC = 2.5 mm. **g** Average oxygen concentration in gyroid scaffolds with varying RDs and UCs. Live/Dead assay showing cell viability in two scaffolds (RD = 20%, UC = 2.5 mm, and RD = 50%, UC = 2.5 mm) on day 7: **h** confocal images, and **i** quantitative analysis (*n* = 3 independent experiments). **j** Elastic modulus of scaffolds with RD = 20%, UC = 2.0, 2.5, 3.0 and 3.5 mm (*n* = 3 independent experiments). **k** CAD model of the lattice scaffold used as a control. Parameters, including cell density, total cell number, and mechanical strength, were matched to those of the gyroid scaffold. **l** Comparison of elastic modulus between the gyroid and lattice scaffolds (*n* = 3 independent experiments). **m** Modelled oxygen concentration in the lattice scaffold at 120 s. **n** Comparison of the average oxygen concentration over time between the lattice and gyroid scaffolds (RD = 20% and UC = 2.5 mm). Data in (**i**), (**j**) and (**l**) are presented as mean ± standard deviation. Statistical significance was determined by a two-tailed *t*-test following the Shapiro–Wilk normality test: *p < 0.05, **p < 0.01, ***p < 0.001, and *p* > 0.05 (not significant). The maximum and minimum values of the colour bars in (**e**), (**f**) and (**m**) are 0.22 and 0.15, respectively. Source data are provided as a source data file.

Of many design requirements, oxygen and nutrient supply, which is heavily influenced by the scaffold architecture, is a paramount concern due to its importance to long-term cellular survival. Among various TPMS structures, the gyroid type was selected in this study because of its design flexibility and near-isotropic physical properties across the volume fractions^39^. The gyroid, defined by an algebraic equation, forms a continuous curved surface that is nearly uniformly distributed throughout 3D space (Fig. 1a). The fundamental geometry of a gyroid can be parametrically defined using a set of variables (Supplementary Note 1), such as the size of the unit cell (UC) and relative density (RD). Reducing RD increased the specific surface area, thinned the wall, and enlarged the pores (Fig. 1b, c and Supplementary Fig. 1), which generally promotes nutrient diffusivity from the surrounding medium into the scaffold, but reduces elastic modulus and total stiffness^39^. UC is another critical parameter in designing the scaffold, which is positively proportional to both the wall thickness and pore diameter, as shown in Fig. 1c and Supplementary Fig. 1. Theoretically, it is possible to reduce the structure to extremely thin features by tuning the core geometric parameters, thereby maximising its permeability. Yet, it encounters weak mechanical strength. Therefore, optimal RD and UC should be explored to simultaneously ensure adequate matter transport and mechanical strength.

A 10 wt% GelMA formulation was selected as the photocurable hydrogel for scaffold fabrication, as it is widely used in 3D tissue engineering and provides a practical balance between structural integrity and biological compatibility^32,40^. The measured elastic modulus was approximately 13.1 kPa (Supplementary Fig. 2), which lies near the lower end of the stiffness range reported for liver tumours (14–72 kPa for hepatocellular carcinoma), thereby offering a mechanically relevant matrix for liver cancer cell culture^41^. An oxygen diffusion model was employed to simulate spatiotemporal oxygen distribution within the scaffolds during culture. The computational domain represented a gyroid scaffold maintained in a semi-closed incubator environment (Fig. 1d). To minimise potential damage to cell membranes and proteins caused by prolonged light exposure during DLP printing^42^, the scaffold dimensions were set to 7 × 7 × 1.5 mm to balance construct thickness with cytocompatibility.

Figure 1e, f shows representative simulations of oxygen concentration within the model domain and its temporal evolution in scaffolds with varying RD (UC = 2.5 mm). At RD = 20%, oxygen was more uniformly distributed and reached steady-state diffusion more rapidly compared to RD = 50%. This difference arises from the thicker walls and smaller pores at higher RD, which hinder oxygen transport and increase diffusion distances. To further evaluate the influence of geometric parameters on permeability, a parametric study was conducted by varying both RD and UC. As noted earlier, increasing RD led to thicker walls (Fig. 1c) and reduced pore size (Supplementary Fig. 1), both of which impaired diffusion. Consequently, oxygen diffusivity decreased monotonically with increasing RD (Fig. 1g). In contrast, increasing UC enlarged pore size (Supplementary Fig. 1), which facilitated diffusion, but also increased wall thickness (Fig. 1c), which could hinder diffusion by extending transport pathways. These opposing effects partially offset one another, resulting in a relatively minor net influence of UC on diffusion (Fig. 1g). The Live/Dead assay of HepG2 cells demonstrated that scaffolds with RD = 20% exhibited substantially higher cell viability compared to those with RD = 50%. A pronounced presence of dead cells (red) was observed within the RD = 50% scaffolds after 7 days of culture. This observation was further supported by quantitative analysis, which showed a decrease in cell viability from approximately 97% to 84% as RD increased from 20% to 50% (Fig. 1i). Overall, these results indicate that lower RD enhances diffusion, whereas UC has a comparatively minor effect.

Another critical consideration is mechanical strength, which must be sufficient to maintain scaffold integrity during bioprinting and after implantation. Although RD = 10% theoretically provided the best material exchange and highest cell viability, it produced a gyroid with extremely thin walls (<90 μm), resulting in significant deformation and distortion after printing. In contrast, RD = 20% could be fabricated reliably, representing an optimal balance between nutrient transport and structural stability. Since UC regulates macropore size and influences mechanical properties, compression tests were performed on scaffolds with UC values of 2.0, 2.5, 3.0, and 3.5 mm (Fig. 1i). Scaffolds with UC = 2.5 mm exhibited the highest compressive modulus (Fig. 1j), providing higher mechanical support beneficial for in vivo applications.

Although UC showed minimum impact on oxygen diffusion, it could alter the curvature of the scaffold, and thereby might provide different geometric cues to cells^34^. Varying UC size (1.7, 2.5 and 3.5 mm) produced different mean Gaussian curvatures (−5.00, −2.30 and −1.25 mm^−2^), corresponding to high, moderate and low curvature magnitudes, respectively. These three UC values were selected because they kept the wall thickness below ~200 μm (Fig. 1c), thereby minimising hypoxia-related effects. Capillary network formation assays were then conducted using these three gyroid scaffolds laden with HUVECs. The relative density was kept constant at 20% to ensure comparable total cell number and high cell viability among the groups. The corresponding cell morphology and quantitative analyses of capillary network parameters are shown in Supplementary Fig. 3, and the scaffold parameters are summarised in Supplementary Table 1. Compared with the scaffold with low curvature magnitude, cells cultured in the scaffold with moderate curvature (−2.30 mm^−2^ with UC = 2.5 mm) exhibited a more elongated morphology and significantly improved capillary network parameters (number of junctions, number of segments, and total network length), indicating enhanced endothelial network formation. However, this promotive effect diminished at the highest curvature magnitude, likely due to increased local geometric confinement. The impact of curvature on endothelial alignment was somewhat minor. To this, geometric parameters (RD = 20% and UC = 2.5 mm) not only provided adequate diffusion capacity and mechanical stability, but also promoted capillary formation more effectively. Therefore, this optimised architecture was used in the subsequent studies.

Lattice scaffolds have been commonly used in 3D tissue engineering^10–12^. To provide a meaningful comparison, a lattice scaffold was designed as a control, with parameters, including material, cell density, total cell number, and mechanical strength, matched to those of the gyroid scaffold (Fig. 1k, l). This design resulted in a wall thickness of approximately 350 µm, comparable to scaffolds previously employed for generating vascularised constructs^43,44^. Consequently, the lattice scaffold exhibited reduced nutrient supply relative to the gyroid, as indicated by lower oxygen concentrations (Fig. 1m, n). We attempted to decrease the lattice wall thickness to levels similar to the gyroid scaffold, but equivalent mechanical strength could not be achieved, leading to structural integrity issues during printing (Supplementary Note 2 and Supplementary Fig. 4).

### Vascularisation of the entire 3D scaffold with a dense microvasculature

**Fig. 2:**
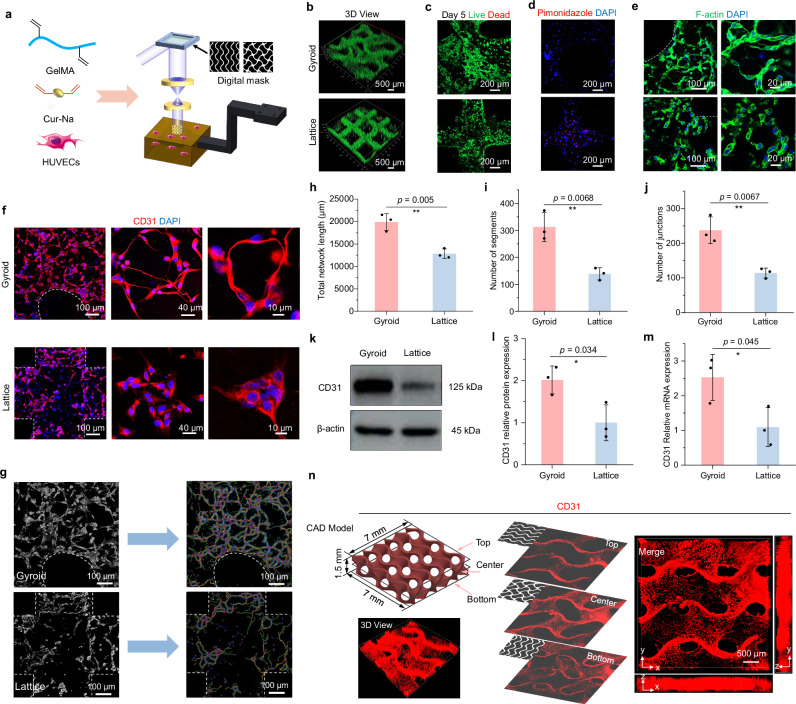
Vascularisation capability of the scaffolds. **a** Schematic illustration of the printing process for scaffolds encapsulating HUVECs. Live/dead assay images showing cell viability on day 5 in the gyroid (upper) and lattice (lower) scaffolds: **b** 3D view and **c** high-magnification images. **d** Pimonidazole staining (red) showing hypoxia within the scaffolds. **e** Cytoskeleton staining of the cells (green: F-actin; blue: DAPI). Cells in the gyroid scaffold displayed enhanced morphogenesis and cytoskeletal remodelling, characterised by elongated morphologies and filopodia formation. **f** CD31 immunofluorescence staining showing endothelial network morphology after 5 days of culture (red: CD31; blue: DAPI). **g** Visualisation of endothelial networks using the Angiogenesis Analyzer plugin in ImageJ. All confocal images shown above are representative of *n* = 3 independent experiments. Quantitative analysis of endothelial network-related parameters, including **h** the total network length, **i** the number of segments, and **j** the number of junctions (*n* = 3 independent experiments). **k** and **l** Western blot analysis for CD31 protein expression (*n* = 3 independent experiments). **m** qPCR analysis for CD31 mRNA expression (*n* = 3 independent experiments). **n** Panoramic 3D view of CD31 expression of the gyroid scaffold, indicating the formation of a dense vascular network distributed uniformly throughout the structure. Data in (**h**–**j**), (**l**) and (**m**) are presented as mean ± standard deviation. Statistical significance was determined by a two-tailed *t*-test following the Shapiro–Wilk normality test: *p < 0.05, **p < 0.01, ***p < 0.001, and *p* > 0.05 (not significant). Source data are provided as a source data file.

One of our primary motivations was to enhance vascularisation throughout the entire 3D scaffold, thereby assisting in developing vascularised models and preventing the formation of necrotic regions in bulk constructs. As with the optimised architecture, we further explored the capability of the gyroid scaffold to support EC self-assembly into vascular networks in a 3D environment. A bioink composed of HUVECs with a high density of 1 × 10^7^ cells ml^−1^ and GelMA hydrogel (10 wt%) was developed and photo-polymerised into 3D cellularised scaffolds using a DLP bioprinter (Fig. 2a). To mitigate the cell-induced light-scattering effect that can substantially deteriorate the printing resolution and geometric accuracy of the fabricated objects, a dual-function and biocompatible photoinhibiting additive (curcumin-Na (Cur-Na)) was added to the bioink^32^. Cur-Na can effectively suppress the scattering effect via a mechanism of simultaneous physical photoabsorption and chemical free-radical reaction. As a result, the gyroid and lattice scaffolds were fabricated successfully with high fidelity, reproducing the digitally designed fine-scale features, such as the interconnected pores and curved thin walls (Fig. 2b and Supplementary Fig. 5). Active perfusion culture through pre-patterned channels was typically required to facilitate the mass transport of large engineered tissues^45^. While effective in promoting nutrient supply in vitro, it is tempered by its complexity and cost in transferring to in vivo applications^3,46^. We sought to examine whether adequate nutrient delivery was achievable in the optimised scaffold without active perfusion, simplifying the culture process. These scaffolds were, therefore, cultured statically in a culture medium supplemented with vascular endothelial growth factor A (VEGF-A, 50 ng ml^−1^).

By day 2, bright-field imaging revealed a noticeable difference in cell spreading between the two scaffold types. Cells within the gyroid scaffold began to elongate and initiated network formation, whereas these behaviours were less evident in the lattice scaffold (Supplementary Fig. 6). After 5 days of incubation, architecture-dependent differences in hypoxia and cell morphology became more pronounced (Fig. 2c–e). Compared with the gyroid scaffold, the lattice scaffold exhibited stronger pimonidazole staining, indicating a more pronounced hypoxic microenvironment (Fig. 2d). Cytoskeletal organisation, assessed by actin staining, also differed between the two architectures (Fig. 2e). Cells in the lattice scaffold remained aggregated with limited spreading, while ECs in the gyroid scaffold displayed enhanced morphogenesis and cytoskeletal remodelling, adopting elongated shapes and extending filopodia. These structural adaptations likely support cell migration, environmental sensing, and intercellular interactions^47^.

To further investigate endothelial network formation after 5 days of in vitro culture, the scaffolds were stained for CD31 by immunofluorescence (Supplementary Fig. 7). The gyroid scaffold supported the formation of filamentous pseudopodia and lumen-like structures, enabling the establishment of complex vascular-like networks through intercellular connections (Fig. 2f). This pattern is a hallmark of healthy vascularisation commonly observed in 3D culture systems using dilute gel-like matrices^48,49^. Although such matrices support network formation, they often lack the structural integrity necessary to produce tissue models of therapeutically relevant size and architecture. In contrast, vascular network formation in the lattice scaffold was markedly suppressed, particularly in the central crisscross regions, where extended diffusion distances may have limited oxygen and VEGF-A availability. For quantitative comparison, endothelial network-related parameters were analysed using the Angiogenesis Analyzer plugin (Fig. 2g). The total network length, number of segments, number of junctions, and alignment index in the gyroid scaffold were approximately 1.5-fold, 2.0-fold, 2.2-fold, and 2.2-fold higher, respectively, than those in the lattice scaffold (Fig. 2h–j and Supplementary Fig. 8), indicating enhanced vascularisation. This enhancement in the gyroid scaffold was further supported by western blot (WB) and real-time PCR (qPCR) analyses, which showed an approximately 2.5-fold increase in CD31 mRNA expression compared with the lattice scaffold (Fig. 2k–m and Supplementary Fig. 9). Moreover, the panoramic 3D view of CD31 expression demonstrated that the gyroid scaffold facilitated the formation of a dense vascular network distributed more uniformly throughout the structure (Fig. 2n), suggesting its superior capacity to support cell growth and vascularisation throughout the entire 3D scaffold.

### The effect of scaffold curvature on cell behaviours

**Fig. 3:**
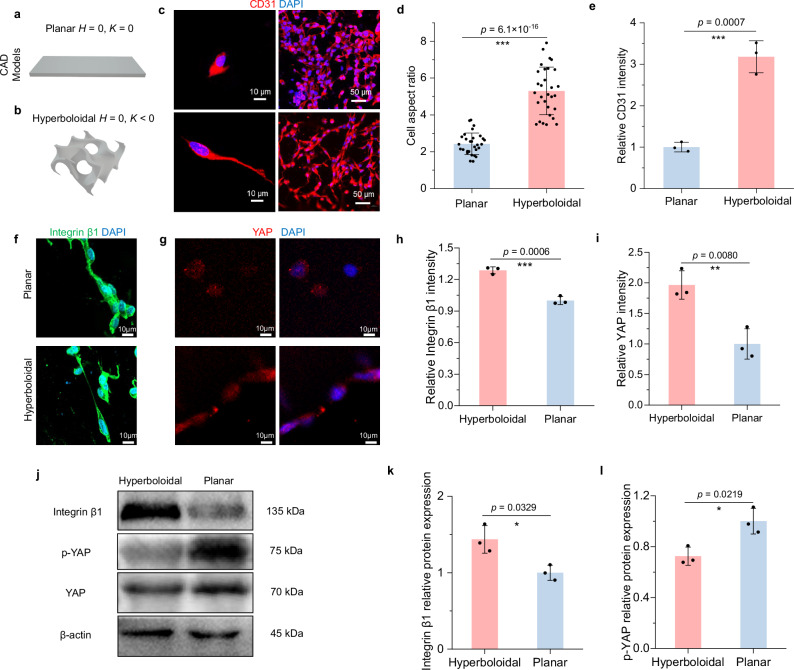
Effects of curvature on HUVEC network formation. **a** CAD model of the planar structure. **b** CAD model of the hyperboloidal structure. Mean curvature (*H*) and Gaussian curvature (*K*) are defined as $({k}_{1}+{k}_{2})/2$ and ${k}_{1}\times {k}_{2}$, respectively, where ${k}_{1}$ and ${k}_{2}$ represent the principal curvatures at a point on the curved surface. **c** Representative CD31 immunofluorescence images (red: CD31; blue: DAPI). **d** Quantitative comparison of cell aspect ratio in (**c**) (*n* = 3 independent experiments; 10 cells were randomly selected from each experiment to ensure statistical robustness). **e** Quantification of CD31 fluorescence intensity (*n* = 3 independent experiments). **f** Integrin β1 immunofluorescence staining in planar and hyperboloidal structures (green: CD31; blue: DAPI). **g** Yes-associated protein (YAP) staining (red: YAP; blue: DAPI). **h** Relative integrin β1 fluorescence intensity (*n* = 3 independent experiments). **i** Relative YAP fluorescence intensity (*n* = 3 independent experiments). **j** Western blot analysis of integrin β1 and YAP protein expression. **k** Integrin β1 relative expression level (*n* = 3 independent experiments). **l** Phosphorylated YAP (p-YAP) relative expression level (*n* = 3 independent experiments). Data in (**d**), (**e**), (**h**), (**i**), (**k**) and (**l**) are presented as mean ± standard deviation. Statistical significance was determined by a two-tailed *t*-test following the Shapiro–Wilk normality test: *p < 0.05, **p < 0.01, ***p < 0.001, and *p* > 0.05 (not significant). Source data are provided as a source data file.

From the perspective of geometric characteristics, the two scaffolds (gyroid and lattice) are 3D structures composed of volumetric entities with different local curvatures, implying that the 3D curvature may play a pivotal role in modulating cellular behaviours. This hypothesis is supported by the well-established principle that cells can sense and respond to biophysical cues, including mechanical properties and geometric features of the extracellular matrix (ECM), via mechanotransduction signalling pathways^33^. To investigate the effects of curvature on vascularisation, ECs encapsulated in scaffolds with different 3D curvatures were assessed. Curvature was quantified using mean curvature ($H=({k}_{1}+{k}_{2})/2$) and Gaussian curvature ($K={k}_{1}\times {k}_{2}$), where ${k}_{1}$ and ${k}_{2}$ are the principal curvatures at a point on the curved surface. Two structures (Fig. 3a), including a gyroid and a plate embodying hyperboloidal ($H=0$ and $K < 0$) and planar ($H=0$ and $K=0$) curvatures, respectively, were designed for the analysis. The wall thickness of both structures was constrained to be ~200 µm to ensure sufficient oxygen diffusion, thereby minimising hypoxia. Other geometric parameters, including size and volume fraction, were adjusted to maintain an equal total cell number across scaffolds. The corresponding cellular constructs were fabricated using the same GelMA bioink containing ECs (1 × 10^7^ cells ml^−1^) and subsequently cultured statically in vitro under identical conditions (5 days) for vascularisation analysis.

We first examined cell morphology, which is known to be sensitive to the type and degree of substrate curvature^33^. Representative CD31 immunofluorescence images (Fig. 3c and Supplementary Fig. 10) show that cells confined to a 3D flat substrate adopted a relatively rounded shape. This contrasts with conventional studies^34,35^ in which cells seeded on 2D surfaces often displayed elongated, cobblestone-like morphologies due to minimal constraints on the apical side. These observations highlight the importance of a 3D microenvironment for studying cell-ECM interactions, as the in vivo environment is intrinsically 3D. Within the gyroid scaffold, ECs exhibited significantly greater elongation and the longest filopodia, suggesting enhanced cytoskeletal reorganisation. Quantitatively, the cell aspect ratio and CD31 expression were approximately 2.5-fold and 3.1-fold higher, respectively, compared with the planar scaffold (Fig. 3d, e). This response is likely attributable to the increased mechanical stimulation induced by the hyperboloidal nature of the gyroid scaffold. VE-cadherin, a key component of adherens junctions, mediates strong cell-cell adhesion through homophilic interactions^3^. VE-cadherin expression in the gyroid scaffold was approximately 3.0-fold higher than in the planar scaffold (Supplementary Fig. 11), indicating greater vessel maturity.

These results motivated us to further investigate the molecular mechanisms underlying the effects of curvature. We examined several mechanotransduction-related proteins, including integrin β1, Yes-associated protein (YAP), and phosphorylated YAP (p-YAP). Immunofluorescence staining demonstrated that the gyroid scaffold upregulated integrin β1 and YAP expression compared with the planar plate (Fig. 3f–i). Western blot analysis further confirmed increased integrin β1 expression. In addition, enhanced nuclear accumulation of YAP and reduced YAP phosphorylation were observed, indicating promoted YAP nuclear translocation (Fig. 3j–l and Supplementary Fig. 12). Overall, these results indicate that hyperboloidal curvature serves as an important geometric cue to which ECs respond.

### Generation of a vascularised liver tumour model with promoted hepatic function

As a proof of concept, we used the highly vascularised liver tumour model for evaluating the ability of the scaffold to support long-term multicellular culture, endothelial network formation, and in vivo tumour tissue development. The immortalised HepG2 cell line was employed as the tumour cell component because of its high oxygen consumption and proliferative rate^50^, making it a suitable model for testing the performance limits of the gyroid scaffold. HepG2 (1.6 × 10^7^ cells mL^−1^) and HUVECs (4 × 10^6^ cells mL^−1^) were mixed in the GelMA hydrogel (10 wt%) at a ratio of 4:1, previously shown to support optimal hepatic function^51^. At this high cell density (2.0 × 10^7^ cells mL^−1^), the gyroid and lattice scaffolds were successfully fabricated with preserved cell viability and geometric fidelity (Supplementary Fig. 13). Still, these scaffolds were cultured statically in medium containing 50 ng mL^−1^ VEGF-A.

**Fig. 4:**
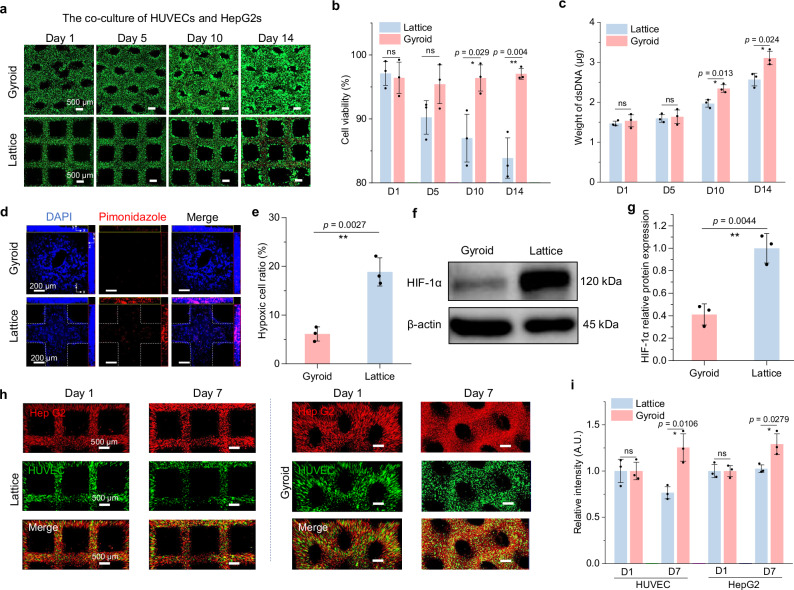
Generation of a vascularised liver tumour model by co-culture of HUVECs and HepG2 over 14 days. **a** Live/dead staining showing cell viability in gyroid and lattice scaffolds for 14 days of culture (live cells: green; dead cells: red). **b** Quantitative comparison of cell viability (*n* = 3 independent experiments). **c** Quantitative comparison of measured genomic DNA content (*n* = 3 independent experiments). **d** Pimonidazole staining for visualisation of hypoxic cells (red: pimonidazole; blue: DAPI). **e** Proportion of hypoxic cells in the scaffolds on day 10 (*n* = 3 independent experiments). **f**, **g** Western blot analysis of HIF-1α protein expression after 10 days of culture (*n* = 3 independent experiments). **h** HepG2 and HUVECs labelled with red and green membrane fluorescent probes, respectively. **i** Relative fluorescence intensity normalised to day 1 for each cell type (*n* = 3 independent experiments). Data in (**b**), (**c**), (**e**), (**g**) and (**i**) are presented as mean ± standard deviation. Statistical significance was determined by a two-tailed *t*-test following the Shapiro–Wilk normality test: *p < 0.05, **p < 0.01, ***p < 0.001, and *p* > 0.05 (not significant). Source data are provided as a source data file.

Live/Dead, genomic DNA, and CCK-8 assays were first performed to assess cell viability, proliferation and cellular metabolic activity, respectively, in the co-cultured scaffolds over 14 days (Fig. 4a–c and Supplementary Fig. 14). The gyroid scaffold maintained consistently high cell viability (~96%) throughout the culture period, whereas viability in the lattice scaffold declined significantly from ~96% to ~83%, likely owing to insufficient oxygen supply (Fig. 4b). Consistent with this trend, cell proliferation in the gyroid scaffold was approximately 1.28-fold higher than in the lattice scaffold, as indicated by genomic DNA quantification (Fig. 4c). Accordingly, cellular metabolic activity in the gyroid scaffold was approximately threefold higher (Supplementary Fig. 14). This enhanced proliferation also altered the structural morphology of the gyroid construct, as confocal imaging on day 14 revealed extensive cellular occupancy within the scaffold pores (Fig. 4a).

Hypoxia-induced cell death is a major barrier to the generation of functional bulk tissue constructs^2^. To assess hypoxic conditions within the scaffolds, pimonidazole staining was performed. By day 10, hypoxia was markedly increased in the lattice scaffold, as indicated by stronger hypoxia-marker staining in orthogonal confocal images (Fig. 4d). Quantitative analysis showed that the proportion of hypoxic cells in the gyroid scaffold was approximately 6%, which was threefold lower than that in the lattice scaffold (Fig. 4e). The superior nutrient transport capacity of the gyroid scaffold was further supported by WB analysis of hypoxia-inducible factor-1 alpha (HIF-1α), a key transcriptional regulator of cellular responses to low-oxygen environments (Fig. 4f, g and Supplementary Fig. 15). Further, we sought to determine which cell type was more susceptible to hypoxia in a co-culture system. HepG2 and HUVECs were then stained with red and green fluorescent colours, respectively, using cell membrane fluorescent probes (Fig. 4h). The number of HUVECs in the lattice was decreased by ~25% on day 7, while the proliferation of both cells was observed in the gyroid (Fig. 4i). These findings suggest that HUVECs, although less tolerant of hypoxia, can remain viable and proliferate in the gyroid scaffold despite competition for oxygen, highlighting the capacity of the gyroid architecture to support multicellular co-culture.

**Fig. 5:**
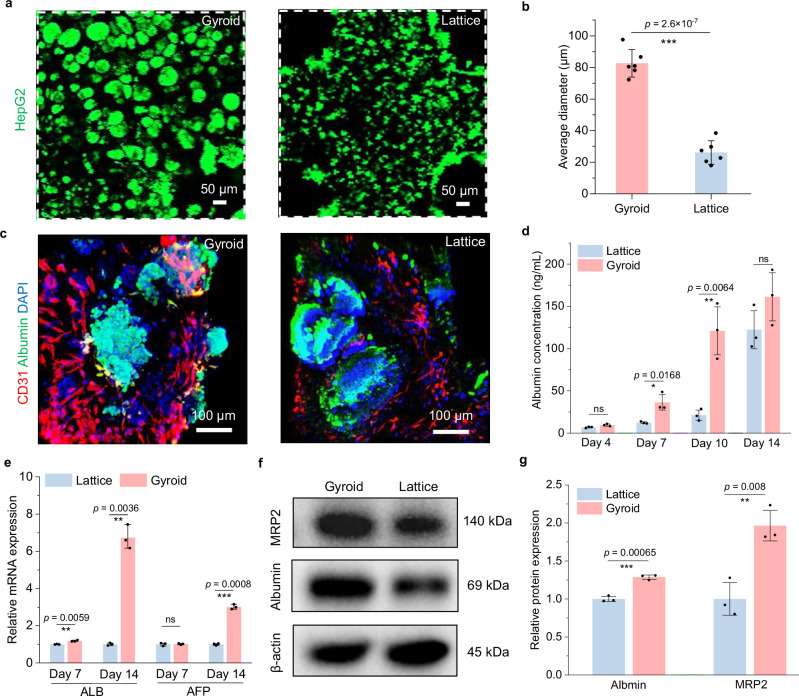
Cell distribution, morphology and liver-specific functions used to assess the maturation of the co-cultured models in vitro. **a** Representative confocal images showing the distribution of HepG2 cells in gyroid (left) and lattice (right) scaffolds after 10 days of culture. **b** Quantification of the diameter of HepG2 aggregates (*n* = 3 independent experiments; two regions of interest per experiment). **c** Co-immunostaining of albumin (ALB) for HepG2 (green), CD31 for HUVEC (red), and DAPI for nucleus (blue), showing the morphology and spatial distribution of the two cell types. **d** Temporal profile of albumin secretion in the scaffolds (*n* = 3 independent experiments). **e** qPCR analysis of the expression of the mature hepatic marker ALB and foetal hepatic marker alpha-fetoprotein (AFP) (*n* = 3 independent experiments). **f**, **g** Western blot analysis of multidrug resistance protein 2 (MRP2), which is associated with drug metabolism, and ALB expression (*n* = 3 independent experiments). Data in **b**), (**d**), (**e**) and (**g**) are presented as mean ± standard deviation. Statistical significance was determined by a two-tailed *t*-test following the Shapiro–Wilk normality test: *p < 0.05, **p < 0.01, ***p < 0.001, and *p* > 0.05 (not significant). Source data are provided as a source data file.

We next examined cell morphology and hepatic function to evaluate the maturation of the co-cultured models. After 10 days of culture, cells in the gyroid self-organised into multicellular clusters (Fig. 5a–c), amorphology that has been associated with tumour development in vivo; such cellular aggregation was not, however, prominent in the lattice scaffold. The average diameter of the spherical aggregates in the gyroid scaffold was approximately four times larger than that in the lattice scaffold (Fig. 5b). To further characterise cellular heterogeneity and spatial distribution, the scaffolds were co-immunostained for albumin to identify HepG2 cells (green), CD31 to identify HUVECs (red), and DAPI to label nuclei (blue), demonstrating the multicellular nature of the aggregates (Fig. 5c). Compared with previously reported 3D co-culture models^12,51^ and with the lattice scaffold used here, the gyroid scaffold exhibited greater vascularisation and structural heterogeneity, with multicellular HepG2 aggregates surrounded by extensive vascular networks. This organisation closely resembles the in vivo architecture of solid tumours. The enhanced tissue organisation is likely attributable to improved mass transport and curvature-induced stimulation provided by the gyroid architecture (Fig. 3), which together promote stronger cell-cell interactions between HUVECs and HepG2 cells^49,52^. In addition to morphology, hepatic anabolic function was evaluated by measuring albumin (ALB) secretion, a representative protein synthesised by liver cells. ALB secretion in the gyroid scaffold increased gradually during the first week, followed by a sharp rise on day 10; in contrast, a similar increase was delayed until day 14 in the lattice scaffold (Fig. 5d). qPCR analysis further showed that the expression of the mature hepatic marker ALB and the foetal hepatic marker AFP was markedly higher in the gyroid scaffold than in the lattice scaffold, particularly on day 14 (Fig. 5e). Consistently, WB analysis demonstrated that the expression of multidrug resistance protein 2 (MRP2), which is associated with drug metabolism, as well as ALB, was higher in the gyroid scaffold on day 14 than in the lattice scaffold (Fig. 5f, g and Supplementary Fig. 16). The expression levels of MRP2 and ALB in the gyroid scaffold increased over time, in contrast to previous study^51^, which reported a decline in both markers in lattice-like structures. The upregulation of liver-specific genes and functional proteins observed in the gyroid scaffold indicates tumour tissue formation with enhanced hepatic phenotype compared to the lattice scaffold.

### Enhanced tumour tissue formation and neovascularisation in vivo

**Fig. 6:**
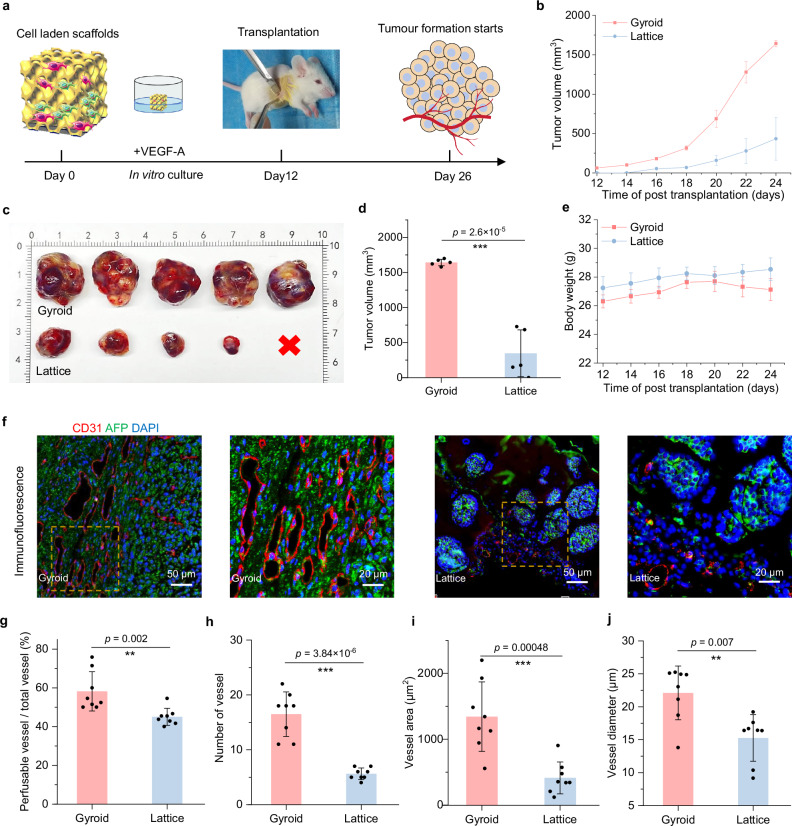
In vivo analysis of engineered tumour tissue constructs. **a** Schematic diagram illustrating the process of an in vivo study. Pre-vascularised liver tumour models were implanted subcutaneously into male severely immunodeficient mice after 14 days of in vitro culture. **b** Temporal change in tumour volume over 24 days after implantation (*n* = 5 independent experiments). **c** Solid tumours harvested on day 24 after implantation, when tumour size reached the ethical limit. **d** Quantitative comparison of tumour volume in (**c**) (*n* = 5 independent experiments). **e** Temporal change in mice body weight over 24 days after implantation (*n* = 5 independent experiments). **f** Immunofluorescence analysis of tumour tissue morphology and vascularisation. Tumour tissues were co-stained for CD31 (red), AFP (green), and DAPI (blue). Quantitative vascular parameters in the tumour tissue, including **g** proportion of perfusable vessel (≥6μm), **h** number of vessels, **i** vessel area, and **j** vessel diameter (*n* = 4 independent experiments, as tumours did not form in one lattice scaffold sample; two images were analysed per experiment). Data in (**b**), (**d**), (**e**), and (**g**–**j**) are presented as mean ± standard deviation. The significant differences of (**b**), (**d**), (**e**), (**h**) and (**i**) were determined by a two-tailed *t*-test following the Shapiro–Wilk normality test, whereas (**g**) and (**j**) were analysed using the two-tailed Mann–Whitney U test: *p < 0.05, **p < 0.01, ***p < 0.001, and *p* > 0.05 (not significant). Source data are provided as a source data file.

To evaluate the in vivo outcomes of the engineered constructs, we subcutaneously implanted the pre-vascularised models into the male severely immunodeficient mice after 14-day culture in vitro (Fig. 6a). The liver tumour formation from the gyroid implant commenced by day 12 post-implantation, whereas it was postponed to day 16 for the lattice (Fig. 6b), suggesting early bioactivity towards tumour formation. This early response is vital for the success of implanted grafts, and a primary issue leading to its therapeutic failure is that the cells encapsulated in the graft run the risk of hypoxia due to delayed ingrowth of functional blood vessels (weeks) from the surrounding host tissue^7^. Compared to the lattice, the development of the tumour proceeded rapidly, particularly after 20 days of post-implantation. By day 24, solid tumours formed within the gyroid scaffold were nearly five times the volume of those in the lattice scaffold (Fig. 6c, d), indicating enhanced tumour tissue formation. Additionally, the body weight of mice implanted with gyroid scaffolds remained consistently lower, likely due to an increased metabolic burden associated with rapid tumour growth (Fig. 6e).

Upon reaching the ethical endpoint on day 24 post-implantation, we analysed tumour tissue morphology and vascularisation using immunohistochemistry and immunofluorescence. To ensure accuracy and eliminate false-positive results, we performed co-staining for CD31, AFP, and DAPI. Many perfusable blood vessels were formed in the tissue from the gyroid scaffold, showing a morphology similar to natural solid tumours (Fig. 6f and Supplementary Fig. 17). However, fewer vessels were formed for the lattice and tumour cells remained clustered. Statistically, the proportion of perfusable vessels, number of vessels, vessel area and vessel diameter were higher in tumour tissues formed from gyroid than that of the lattice by ~18%, ~3.0-folds, ~2.8-folds and ~34% (Fig. 6g–j), respectively, indicating promoted neovascularisation. GelMA hydrogels degrade primarily through enzymatic processes, particularly via matrix metalloproteinases such as collagenase. The degradation rate depends on material properties, processing parameters, and the type and activity of encapsulated cells. In this study, no residual hydrogel was detected in tumour tissue derived from the gyroid scaffold, indicating enhanced degradation. In contrast, residual hydrogel was observed in the lattice scaffold sample, where tumour formation did not occur (Fig. 6c). In vivo, cells and tissue infiltrate and fill the hole of a scaffold through a complex process of cellular infiltration, migration, proliferation, and angiogenesis, guided by the scaffold’s porous architecture and material properties^53^. Minimal empty space was observed in tumour tissue derived from the gyroid scaffold, whereas substantial void regions were present in tissue from the lattice scaffold (Supplementary Fig. 17), likely due to its larger pore size and reduced cellular activity.

After 24 days of in vivo cultivation, no fibrotic encapsulation was observed at the implantation site, nor was there any evidence of non-specific inflammatory responses in the surrounding tissues. Furthermore, major organs, including the heart, lung, kidney, spleen, and liver, exhibited normal histological features (Supplementary Fig. 18), demonstrating the good biocompatibility of the scaffolding system.

## Discussion

A major bottleneck in engineering 3D vascularised tissue models, whether through technology-driven strategies or by exploiting intrinsic cellular self-organisation, is the limited ability of current scaffolds to simultaneously satisfy the mechanical, physicochemical, and biological requirements necessary for both in vitro and in vivo applications^15,18,26,28^. One of the central challenges is the trade-off between nutrient transport and structural stability. To preserve construct integrity, scaffolds with relatively high solid volume fractions are often used^5^, but these can promote hypoxia in regions distant from perfusion channels. Alternatively, scaffold designs are frequently restricted to thin and relatively simple geometries unless combined with active perfusion systems, which increase technical complexity and may expose the construct to poorly controlled shear stress^10,11,13,24,46^. In this study, we addressed this challenge by exploiting the intrinsic geometric advantages of TPMS architectures, together with structural optimisation and high-precision cell bioprinting. The porous, thin-walled gyroid architecture enabled improved oxygen diffusion throughout the scaffold while preserving structural stability, owing to its periodic minimal surface geometry, which intrinsically minimises stress concentration and distributes mechanical loads efficiently^36^. In addition, we found that the 3D curved features of the gyroid, particularly at moderate curvature, promoted endothelial cell-mediated vascular self-assembly, potentially through activation of integrin and YAP-related signalling pathways. Compared with the lattice scaffold, the gyroid scaffold showed superior structural continuity, higher printing fidelity, greater stability at comparable RD, and improved biological performance (Supplementary Table 2). Together, these advantages enabled the generation of a highly pre-vascularised bulk liver tumour model in vitro with more physiologically relevant function, morphology, and heterogeneity, while also enhancing neovascularisation and tumour tissue formation after implantation.

Although gyroid scaffolding systems have shown promise in bone tissue engineering^38,54^, their translation to soft tissue models is nontrivial due to fundamental differences in the ECM microenvironment, cellular properties, and cell-matrix interactions. Gyroid scaffolds designed for bone regeneration are typically fabricated from stiff materials such as wollastonite bioceramics or β-tricalcium phosphate, which exhibit elastic moduli in the MPa range and provide load-bearing support suitable for osteogenesis^36^. In contrast, soft tissues require a compliant 3D matrix environment. To mimic this setting, we employed 10 wt% GelMA hydrogels, achieving an elastic modulus of approximately 13.1 kPa, comparable to that of native liver tumour tissue. As matrix stiffness is a key mechanical cue regulating cell behaviour, such differences in material properties are expected to result in distinct biological responses^33^. In addition to mechanical properties, cell incorporation strategies differ between bone and soft tissue scaffolds. In most bone tissue engineering studies^37,38,54^, cells are seeded onto the scaffold surface after fabrication, resulting in relatively low cell densities and limited infiltration into the scaffold interior. In contrast, soft tissues are characterised by a dense and homogeneous 3D cellular distribution. To better recapitulate this physiological architecture, we encapsulated cells directly within the scaffold, generating a bulk, cellularised construct with a high cell density (2 × 10^7^ cells mL^−1^). A major challenge in this context is that soft, cell-laden bioinks often suffer from poor printability due to their dilute polymer content and cell-induced light scattering, particularly in complex 3D architectures with fine features^5^. By leveraging the light-scattering inhibition strategy established in our previous work^32^, we successfully fabricated cell-laden gyroid scaffolds with high structural fidelity. Furthermore, cell-matrix interactions differ between cell-seeded and cell-laden scaffolds. In cell-seeded curved scaffolds, cells mainly adhere to the scaffold surface and sense matrix properties through integrin-mediated adhesion and actomyosin-generated traction forces, which promote focal adhesion maturation and downstream signalling. Consistent with this, Yang et al. showed that the hyperboloidal curvature of gyroid scaffolds enhanced focal adhesion organisation and stress fibre formation in surface-seeded human mesenchymal stem cells^38^. In contrast, in our cell-laden system, cells were confined within a 3D curved matrix, where mechanosensing could be governed by integrin binding and clustering, spatial confinement, and dynamic cell-matrix remodelling^33^. Under these conditions, cells exhibited distinct cytoskeletal organisation, with more pronounced actin assembly and filopodia/lamellipodia formation. For mechanosensing behaviour, it has been demonstrated that increasing the curvature of gyroid scaffolds could promote osteogenic differentiation of human mesenchymal stem cells via focal adhesion kinase (FAK) activation^38^. Compared with lattice scaffolds composed of planar struts, we observed that the gyroid scaffold upregulated integrin β1 expression in ECs, which was accompanied by enhanced morphogenesis and network formation.

Despite the well-recognised advantages of gyroid architectures in bone tissue engineering, their application in cell-laden soft tissue engineering remains limited because the requirements for soft bioinks, print fidelity, and biological support differ substantially from those of hard tissue systems. Existing studies^31,32,55^ have largely focused on demonstrating printability or have been restricted to short-term culture of a single cell type. For example, Huh et al.^31^ successfully fabricated a centimetre-scale cell-laden hydrogel gyroid scaffold, but did not report long-term cell viability or functional outcomes. Recently, Moon et al.^56^ bioprinted a thick gyroid construct laden with HepG2 cells, followed by coating the interconnected vascular channels with ECs. Compared with the lattice scaffold, the gyroid construct achieved a more uniform cell distribution and higher cell viability under perfusion conditions, owing to more homogeneous fluid flow and optimised surface shear stress. This finding further underscores the importance of scaffold structural optimisation. In the present study, we selected 10 wt% GelMA that supported high cell viability and provided a mechanically relevant matrix for liver cancer cell culture. Liver tumour stiffness is generally higher than that of normal liver (1–7 kPa) and varies with disease stage, increasing from fibrosis to cirrhosis and ultimately hepatocellular carcinoma, with representative ranges of 4–13 kPa, 9–25 kPa, and 14–72 kPa, respectively^41^. The material stiffness of our 10 wt% GelMA was approximately 13.1 kPa, which lies at the lower end of the tumour-associated stiffness ranges. We then optimised the scaffold parameters to achieve adequate mass transport and mechanical stability, and appropriate curvature cues. This strategy produced a thin-walled gyroid architecture (~170–210 µm) with a moderate curvature that supported the formation of a dense and uniformly distributed vascular network throughout the entire 3D volume. This is important because one of the major challenges in engineering large tissue constructs is the development of hypoxic microenvironments in the scaffold core, which can lead to cellular dysfunction and necrosis^7^. In contrast, the lattice scaffold with an equivalent wall thickness exhibited severe structural instability because of the limited intrinsic mechanical strength of 10 wt% GelMA. The gyroid architecture could compensate for this weakness through its structural resilience, providing sufficient mechanical stability for both in vitro and in vivo studies. Nevertheless, the construct remained subject to relatively low compressive load tolerance and was therefore unsuitable for load-bearing applications such as bone and cartilage. For such applications, GelMA could be combined with reinforcing materials (e.g., alginate/methylcellulose or PEGDA) to enhance the mechanical performance of printed constructs^57,58^.

Oxygen transport in scaffold-based constructs is generally limited to a few hundred micrometres, placing cells located far from a perfused boundary at risk of hypoxia^2,7^. In this context, a scaffold height of 1.5 mm remains relatively large for a cell-laden construct compared with those used in many previous studies^8–10,24^. Thus, although the present scaffold does not yet represent a fully scaled tissue model, its height, together with the high cell density used in this study, creates a sufficiently diffusion-constrained system to evaluate whether the gyroid architecture can support vascular network formation and multicellular function throughout a truly 3D cell-laden construct. The selected scaffold height was also influenced by the layer-by-layer nature of DLP bioprinting. Increasing scaffold height requires greater cumulative light exposure, which extends cellular exposure to free radicals generated during photocrosslinking and may compromise viability by damaging cell membranes and proteins^42^. A height of 1.5 mm, therefore, provided a practical balance between construct thickness, printing fidelity, and cytocompatibility. Despite the advantages of the gyroid architecture, scaling this model to larger constructs will likely require more efficient bioprinting technologies, such as volumetric additive manufacturing based on tomographic reconstruction^59^.

Geometric curvatures present in ECM have only recently been recognised as a physical cue regulating cellular behaviours, with most studies focusing on cells cultured in 2D systems, such as on curved substrates^33,34,60^. In natural vasculature, ECs, which are often embedded in a 3D matrix, are exposed to curvature cues of various geometries (e.g. cylindrical curvature of vessels and hyperbolic curvature at vascular bifurcation), with magnitudes ranging from 0.1 to 200 mm^−1^^35^. Park et al. showed that tumour cells located in regions of higher vascular curvature within 3D bioprinted multilayered cerebrovascular conduits could activate the vascular barrier and disrupt endothelial junctions through increased secretion of inflammatory cytokines, highlighting the biomechanical role of curvature in regulating local microenvironments^61^. In this study, we demonstrated that a 3D curved volumetric architecture acts as an important geometric cue capable of directing endothelial behaviour and function via mechanotransduction. Integrins, serving as physical signal receptors, have been identified as central regulators of mechanotransduction in 3D, mediating cytoskeletal organisation and the formation of protrusive actin structures such as filopodia. We found that the 3D curved architecture of the gyroid scaffold, with a moderate mean Gaussian curvature of −2.30 mm^−2^, was associated with upregulation of integrin β1. Elevated integrin dynamics enable cells to better sense and respond to the physical properties of the ECM, which can lead to activation of various downstream pathways and transcription regulators^33^. Among these, the YAP signalling pathway has been shown to play an important role in regulating endothelial cell migration, proliferation, vascular remodelling and vessel formation^62^. Li et al.^54^ reported that gyroid scaffolds could suppress YAP/TAZ phosphorylation and enhance YAP/TAZ nuclear translocation of HUVEC seeded on scaffold surface, resulting in enhanced proliferation, migration, tube formation, and high expression of CD31. Consistent with this, in our soft, cell-laden gyroid scaffold, we observed a significant increase in nuclear YAP levels accompanied by reduced YAP phosphorylation, indicating enhanced YAP nuclear translocation. It is noteworthy that the promotive effect on network formation was weakened significantly at a higher curvature (e.g., 5.00 mm^−2^), likely because of increased local geometric confinement. Curvature-dependent changes in focal adhesion organisation, actin stress fibres, and traction force have been reported across curved and confined microenvironments, suggesting that excessive curvature may limit the spreading and force transmission needed for efficient cell migration and network formation^63,64^. Collectively, these results identify 3D curvature as a critical biophysical cue regulating endothelial behaviour and highlight its potential to be harnessed in the rational design of bulk tissue-engineered constructs.

Pre-vascularisation of in vitro tissue constructs has emerged as an effective strategy for promoting graft vascularisation in vivo^2,3,65^. Several approaches have been used to enhance pre-vascularisation before implantation, including the incorporation of angiogenic growth factors, co-culture of ECs with stromal or perivascular support cells, immobilisation of bioactive molecules, and scaffold-based strategies^66^. In the present study, our aim was to demonstrate that the 3D architecture of the gyroid scaffold can facilitate vascularisation and improve the viability and function of co-cultured parenchymal cells in vivo by supporting the formation of preliminary vascular structures in vitro. We used ECs alone to eliminate confounding contributions from support cells, particularly their effects on vessel stability and maturation, so that differences in in vitro and in vivo outcomes could be attributed more directly to scaffold geometry. Following implantation, maturation of EC-lined structures can still occur through recruitment of host support cells, provided that the construct offers a permissive microenvironment for host-graft integration^3^. In addition, HepG2 cells were used as a reproducible hepatic tumour cell source to establish a vascularised 3D tumour model, enabling proof-of-concept evaluation of scaffold-supported multicellular organisation, endothelial network formation, and in vivo tissue development. Translation of the gyroid model to the in vivo setting markedly enhanced tumour tissue formation and vascularisation, highlighting the critical importance of in vitro maturation for in vivo performance. This improvement is likely attributable to the optimised vascularisation and hepatic function established before implantation, together with the structural advantages of the gyroid scaffold, which continued to provide a supportive microenvironment for cell growth and host integration after implantation. These advances are particularly relevant to cell-based therapies such as islet microcapsule transplantation, which often encounters excessive fibrosis in vivo because of insufficient nutrient supply and poor vascularisation^67^. Compared with conventional tumour xenograft models^68^, the use of 3D vascularised scaffolds for tumour generation offers a more efficient strategy and produces tumours with morphology and function that more closely resemble physiological conditions. This approach, therefore, holds promise for advancing cancer research and drug development by providing more physiologically relevant models for studying tumour progression and therapeutic responses.

In summary, we successfully designed and fabricated a soft hydrogel gyroid scaffold and demonstrated its suitability for both in vitro and in vivo applications through the development of a vascularised 3D liver tumour model. By integrating numerical modelling with experimental validation, we identified optimal geometric parameters that balanced mass transport with the mechanical strength required for implantation. This optimisation yielded a structurally complex architecture characterised by interconnected channels, thin walls, and continuously varying Gaussian 3D curvature. Our findings further demonstrated that the gyroid scaffold effectively supported HUVEC culture and enabled vascularisation throughout the entire 3D construct, producing a dense microvascular network. In addition, we found that the continuously varying Gaussian curvature played an important role in promoting endothelial cell-mediated vascular self-assembly, suggesting that scaffold geometry itself can serve as a physical cue for regulating cell behaviour in 3D environments. The scaffold also supported high-density multicellular co-culture of HepG2 cells and HUVECs, enabling the generation of a highly vascularised liver tumour model with more physiologically relevant function, morphology, and heterogeneity. Following subcutaneous implantation in mice, the constructs formed tumours rapidly and exhibited enhanced neovascularisation and tumour morphology resembling that of native solid tumours. Collectively, this study introduces a biologically favourable scaffold architecture for generating highly vascularised tumour models and highlights its potential for soft tissue engineering applications.

## Methods

### Numerical modelling of oxygen diffusion in the scaffolds

The geometric models of the gyroid scaffold were generated using MSLattice software, and the detailed generation process is available in Supplementary Note 1. The lattice scaffold was designed with Computer-Aided Design (CAD) 2021 software. Each scaffold was placed in a cylindrical incubator (9 mm diameter × 10 mm height), representing a typical culture configuration composed of two distinct regions: a nutrient medium region and a porous scaffold region. The scaffold was centrally positioned 3 mm above the base and fully immersed in the nutrient medium. The initial oxygen concentration in the culture medium was set to 0.119 mol m^−3^^69^, and an open boundary condition was applied at the upper surface of the incubator. The diffusion coefficient for the GelMA material was set to 3.093 × 10^−9^ m^2^ s^−1^^69^. The model was implemented in COMSOL Multiphysics software and solved using the finite element method (FEM). The oxygen diffusion was numerically simulated using the transient diffusion equations, capturing the transition from initial hypoxic conditions to steady-state oxygenation across the scaffold architectures.

### Curcumin-Na synthesis

Curcumin-Na (Cur-Na) was synthesised following the procedure outlined in our previous study^32^. Specifically, 368 mg of curcumin (Aladdin) was dissolved in a mixture of 4 mL methyl alcohol (Sigma-Aldrich) and 4 mL distilled water, with continuous stirring until complete dissolution. Subsequently, 294 mg of sodium bicarbonate (NaHCO_3_, Sigma-Aldrich) was added to the solution and allowed to react overnight at room temperature. The reaction mixture was then evaporated using a rotary evaporator, and the resulting residue was dissolved in 3 mL dichloromethane (Sigma-Aldrich), followed by filtration to remove any impurities. Finally, the solution was freeze-dried to obtain Cur-Na as a solid powder. The resulting Cur-Na powder was stored at −20 °C until further use.

### Preparation of hydrogels

For the fabrication of cell-laden scaffolds, photocurable hydrogels were prepared using 10 wt% Gelatin methacryloyl (GelMA, Black Flame Medical, Shanghai, China) and 17 mM lithium phenyl-2,4,6-trimethylbenzoylphosphinate (LAP, Yinchang New Materials Co., Ltd., Shanghai, China). To enhance printing resolution, 1 mM Cur-Na was incorporated as a photoinhibiting additive. To ensure a homogeneous distribution of cells within the fabricated scaffold, 0.1 wt% xanthan gum (G810381, Macklin) was also added.

### Cell culture

HepG2 cells were obtained from Procell Life Science & Technology Co., Ltd., China (CL-0103), while HUVECs were sourced from the National Collection of Authenticated Cell Cultures, China (SCSP-5535). To support both endothelial and tumour cells in the culture system in a unified medium, all cells were cultured and maintained in high-glucose Dulbecco’s modified Eagle’s medium (DMEM, Gibco), supplemented with 10% foetal bovine serum (FBS, Gibco) and 1% antibiotic solution (streptomycin, 100 μg mL^−1^, and penicillin, 100 units mL^−1^, Sigma-Aldrich Corp.). The culture medium was refreshed every two days. Cells were incubated in a humidified atmosphere at 37 °C with 5% CO_2_ and 95% air, and were passaged every 3–4 days. Cell morphology was visualised using the DinoCapture 2.0 software of the Dino-Lite microscope (Dunwell Tech, Inc., USA). HUVECs between passages 3 and 8 were used throughout the study in order to maintain high viability and preserve endothelial marker expression. HepG2 between passages 7 and 18 was used to ensure high metabolic activity.

### 3D bioprinting of cell-laden scaffolds

For the fabrication of vascularised scaffolds, HUVECs were incorporated into the photocurable hydrogels to create a bioink with a cell density of 1 × 10^7^ cells mL^−1^. A range of cell concentrations, including 5 × 10^6^, 1 × 10^7^, 1.5 × 10^7^, and 2 × 10^7^ cells mL^−1^, was tested. It was observed that HUVECs exhibited optimal proliferation and blood vessel formation at a concentration of 1 × 10^7^ cells mL^−1^. For the liver tissue model, HepG2 cells (1.6 × 10^7^ cells mL^−1^) and HUVECs (4 × 10^6^ cells mL^−1^) were incorporated into the hydrogels. The cell concentrations were optimised based on cell viability and proliferation assays, with the HepG2 to HUVEC ratio of 4:1 (also used by Fang et al.^51^) being selected. All scaffolds were fabricated using a projection micro stereolithography (PμSL) printer (NanoArch S140, BMF Material Technology Inc., Shenzhen, China) at approximately 37 °C. The printer was equipped with a 405 nm projector, providing an x-y resolution of 10 μm. Sample slicing was performed using the BMF 3D Slice 1.6.2 software. A light intensity of 13 mW cm^−2^ was applied for each layer (with a layer thickness of 100 μm). 2.5 mL^−1^ bioink was used for each printing, allowing the production of 6 scaffolds (3 gyroid scaffolds and 3 lattice scaffolds) per time. After printing, the scaffolds were washed with PBS for 3 min to remove any unreacted hydrogel. All printing procedures were performed under aseptic conditions in a cleanroom environment. Prior to printing, all equipment and materials were sterilised using ultraviolet irradiation and disinfected with 70% ethanol. Components were handled using sterile techniques wherever applicable. The printer was equipped with a protective chamber in which photocrosslinking occurred, minimising exposure to the external environment. All handling steps were conducted in a controlled manner to maintain sterility.

### Mechanical properties

Compressive stress-strain testing of the printed structures was performed using a tensile-compressive tester (Shimadzu-AGS-X with a 20 N sensor). The samples were fabricated from the same batch of bioink, and three for each structure were used to calculate the statistical results. Due to the size limit of the bioink container of the printer (2.5 mL^−1^), a maximum of 6 samples were allowed to be fabricated per printing. Therefore, the samples might be fabricated using different printing. To assess batch-to-batch reproducibility, three samples of the gyroid scaffold with a relative density of 20% and unit cell size of 2.5 mm were fabricated from independent experiments, including separate bioink preparation and printing process (Supplementary Fig. 19). The hydrogels were composed of 10 wt% GelMA, 17 mM LAP, and 1 mM Cur-Na. The structures were fabricated using the PμSL printer with the same layer thickness and light intensity conditions as above. The printed structures were incubated in PBS for 4 h prior to testing. The compression rate was set to 1 mm min^−1^, and the data were recorded using Trapezium Lite X software of the tester. The elastic modulus of the printed structures was determined by calculating the slope of their stress-strain curves at a strain value of approximately 20%. All tests were conducted in triplicate to ensure consistency and reliability of the results.

### Live/dead assay

The cell-laden scaffolds were cultured in 24-well plates for 0, 1, 5, 10, and 14 days to observe and quantify cell proliferation. The culture medium was supplemented with 50 ng mL^−1^ of vascular endothelial growth factor (VEGF, 100-20-1MG, Thermo Fisher Scientific). The samples were stained using the Live/Dead Assay Kit and visualised with a confocal microscope (Zeiss LSM 980, Germany). Confocal images were captured using ZEN 2012 software. Cell proliferation was quantitatively assessed using the Cell Counting Kit-8 (CCK-8, Meilunbio, Dalian, China) according to the manufacturer’s instructions. Cell viability was calculated by determining the ratio of live cells to total cells, analysed using ImageJ software 1.53. At least three samples per group were tested to ensure statistical validity.

### Genomic DNA extraction and quantification

Genomic DNA was individually extracted from each sample scaffold using the Genomic DNA Extraction Kit (G3633-50T, Servicebio) according to the manufacturer’s instructions. The purified DNA was eluted and dissolved in 100 μL of TE buffer. DNA concentration and purity were assessed using a spectrophotometer (NanoDrop Lite Plus, Thermo Fisher Scientific). The absorbance ratios at A260/A280 for all samples ranged between 1.8 and 2.0.

### Cell membrane fluorescent staining

To visualise the distribution and proliferation of the two distinct cell types within the liver tissue scaffolds, HepG2 cells and HUVECs were stained with different fluorescent dyes prior to printing. HepG2 cells were labelled with a green cell membrane fluorescent probe (DiO, 1:1000, Yeasen Biotechnology, China), while HUVECs were stained with a red cell membrane fluorescent probe (DiR, 1:1000, Yeasen Biotechnology, China). Working solutions of DiO and DiR were prepared in PBS separately. Each cell type was incubated for 20–30 min at 37 °C, followed by three centrifuge-assisted washes in PBS to remove unbound dye. The distribution of the stained cells within the scaffolds was visualised using the ZEN 2012 software of a confocal microscope (Zeiss LSM 980, Germany).

### Immunfluorescence Staining

For the hypoxic cell staining, the cell-laden scaffolds were removed from the culture medium on days 5 and 10 and washed three times with PBS. The samples were then incubated with the culture medium containing pimonidazole hydrochloride (200 μM, hypoxyprobe-1, USA) in a humidified atmosphere incubator at 37 °C for 2 h. The samples were fixed in 4% formaldehyde solution for 30 min and rinsed in PBS three times. Then the samples were soaked in 0.1% triton-X 100 for 30 min and blocked with 10% FBS in PBST solution for 1 h. Subsequently, the samples were incubated with anti-pimonidazole rat IgG1 monoclonal antibody (1:50, MAb1, USA) at 4 °C for 1 h. After washing three times with PBS, the samples were counterstained with 4′,6-diamidino-2-phenylindole (DAPI, 1:1, GTX30920, GeneTex) for 20 min. Confocal images were visualised using the ZEN 2012 software of a confocal microscope (Zeiss LSM 980, Germany).

For the functional protein staining, the samples were washed three times with PBS, fixed in 4% formaldehyde solution for 30 min, and rinsed three times with PBS. The samples were then permeabilised in 0.1% triton-X 100 for 30 min and blocked with 10% FBS in PBST solution for 30 min. The samples were incubated overnight at 4 °C with primary antibodies in antibody diluent solution, followed by washing with PBS. Then, the samples were treated with the corresponding secondary antibody solution and phalloidin. Finally, the samples were counterstained with DAPI (1:1, GTX30920, GeneTex) for 20 min and visualised by confocal microscope (Zeiss LSM 980, Germany). Primary antibodies were used as follows: anti-CD31 (1:100, Cat: 66065-2-Ig, clone: 3F8E2, Proteintech), anti-VE-cadherin (1:200, Cat: D87F2, Cell Signaling), anti-albumin (1:500, Cat: ab207327, clone: EPR20195, Abcam). ABflo 594-conjugated Goat Anti-Mouse IgG (H+L) secondary antibody (1:200, Cat: AS054, ABclonal), Alexa Fluor 488 Goat Anti-Rabbit IgG (1:1000, Cat: ab150077, Abcam), and secondary antibody (1:200, Cat: AS073, ABclonal) were used for counterstain. Additionally, Alexa Fluor 488 phalloidin (1:1000, Cat: AC18L032, Life-iLab) was applied to visualise the F-actin.

### Endothelial networks analysis

The morphology of HUVECs on day 5 in gyroid and lattice scaffolds was recorded by confocal microscopy. The Angiogenesis Analyzer Plugin in ImageJ was employed to quantify vascular networks using a default setting. For each scaffold type, three images from independent samples were analysed, and one region of interest per image was selected for statistical evaluation. Among the generated angiogenic statistical parameters, the number of junctions, the number of segments, the total segment length, and the alignment index were chosen for quantitative analysis.

### Measurement of albumin secretion and urea synthesis

To assess hepatic function, cell culture supernatants from each group were collected for 4, 7, 10, and 14 days and stored at −20 °C before use. The culture medium was changed daily. Three samples for each group were grown in one well of 24-well plates. The albumin was evaluated using the human albumin ELISA (Enzyme Linked Immunosorbent Assay) kit (SEKH-0081, Solarbio) according to the manufacturer’s instructions. The concentration of albumin per day was calculated from the standard curve of the experiment.

### Western blotting

The cell-laden scaffolds were placed in a pre-cooled mortar and homogenised on ice following the addition of RIPA lysis buffer (CW2333S, Cwbio) supplemented with protease inhibitors. The extracted protein was subsequently quantified using the BCA Protein Assay Kit (P0011-1, Beyotime) in accordance with the manufacturer’s protocol. Protein samples were then separated via sodium dodecyl sulfate-polyacrylamide gel electrophoresis (SDS-PAGE) and transferred onto polyvinylidene difluoride (PVDF) membranes (Millipore, USA). For each comparison, all samples from independent experiments were placed onto the same membrane to reduce technical variance. The membranes were incubated overnight at 4 °C with the respective primary antibodies. Following incubation, the membranes were washed with TPST and further incubated with secondary antibodies for 2 h at room temperature. Protein expression levels were visualised using the Omega Lum G Capture Software of the Aplegen imaging system (Omega Fluor, USA) and analysed with ImageJ software. The primary antibodies utilised included anti-HIF-1α (1:200, Cat: ab51608, clone: EP1215Y, Abcam), anti-CD31 (1:10000, Cat: ab76533, clone: EPR3094, Abcam), anti-MRP2 (1:1000, Cat: ab172630, clone: EPR10998, Abcam), anti-albumin (1:2000, Cat: ab207327, clone: EPR20195, Abcam), anti-β-actin (1:1000, Cat: 4970, Cell Signaling), anti-YAP1 (1:5000, Cat: ab52771, clone: EP1674Y, Abcam), anti-YAP1(phospho S127) (1:10,000, Cat: ab76252, clone: EP1675Y, Abcam), and anti-Integrin-β1 (1:10,000, Cat: A23497, clone: ARC52470, ABclonal). The secondary antibodies employed were goat anti-rabbit IgG (H+L) Cross-Absorbed secondary antibody (1:2000, Cat: ab6721, Abcam). The raw blots are available in Supplementary Figs. 9, 12, 15 and 16.

### Real-time PCR (qPCR)

Total RNA was extracted from samples using TRIzol reagent (15596026, Invitrogen, Thermo Fisher Scientific). Complementary DNA (cDNA) was synthesised by reverse transcription using the EasyScript R All-in-One First-Strand cDNA Synthesis SuperMix for qPCR (One-Step gDNA Removal) (AE341-02, TransGen Biotech, China). Quantitative real-time PCR was performed using the Applied Biosystems 7500 system (Thermo Fisher Scientific, Waltham, MA, USA) with 2× PerfectStart R Green gPCR SuperMix (+Dye II), and the data were collected using the HMR software 2.0 of the system. The amplification protocol consisted of an initial preheating step at 50 °C for 2 min, followed by enzyme activation at 95 °C for 15 s, denaturation at 95 °C for 15 s, and annealing at 60 °C for 60 s per cycle (40 cycles in total). The relative expression levels of target genes were determined using the 2^−ΔΔCt^ method, with β-actin serving as the internal reference gene for normalisation.

Primers for target genes were designed based on coding sequences obtained from the NCBI GenBank database. The sequences were submitted to Primer3 Plus with the following parameters: primer length of 18–22 bp, melting temperature of 55–65 °C, maximum Tm difference of 2 °C between forward and reverse primers, GC content of 40–60%, and amplicon length of 100–300 bp. Complementarity at the 3′ ends and self-complementary sequences were avoided to minimise primer dimer and hairpin formation. To reduce amplification of residual genomic DNA, primers were designed to span at least one exon–exon junction. Candidate primer pairs were further checked for specificity using NCBI Primer-BLAST against the human reference genome GRCh38 RefSeq database. The final primer sequences are provided in Supplementary Table 3. All primers were synthesised by a commercial supplier (Tsingke Biotechnology) and provided in lyophilised (dry powder) form.

### In vivo experiments

The animal studies were approved by Central South University (approval number: CSU-2023-0377). Male severely immunodeficient (NOD/ShiLtJGpt, NCG) mice, aged five weeks, were used for the experiments and were purchased from Chengdu Dossy Experimental Animals Co., Ltd. The mice were provided with feed sourced from Hunan Slack Jingda Experimental Animal Co., Ltd. and housed under controlled environmental conditions (22 °C, 50% relative humidity, and 12-h light/dark cycle). Following a one-week acclimatisation period, the mice were randomly assigned to two experimental groups: the gyroid group and the lattice group. Prior to scaffold implantation, the mice were anaesthetised using 2–3% isoflurane. A subcutaneous incision was made on the back of each mouse, separating the skin and muscle to allow implantation of either the lattice or gyroid scaffolds (*n* ≥ 5) into the incision. Tumour volume was measured daily post-transplantation, and specimens were collected before the ethical endpoint was reached. By day 24 post-implantation, tumours in the gyroid group approached the ethical limit (tumour volume <2000 mm^3^ and maximum diameter <20 mm). Finally, liver tumour tissues from both the gyroid and lattice groups were excised for histological analysis.

### Immunohistochemistry

Immunohistochemical analysis was performed on paraffin-embedded tissue sections, which underwent gradient dehydration using ethanol. Antigen retrieval was conducted, followed by incubation with primary antibodies, including anti-CD31 (1:100, Cat: 66065-2-Ig, clone: 3F8E2, Proteintech) and anti-albumin (1:500, Cat: ab207327, clone: EPR20195, Abcam). Immunofluorescence staining was carried out using ABflo 594-conjugated goat anti-mouse IgG (H+L) secondary antibody (1:200, Cat: AS054, ABclonal) and Alexa Fluor 488-conjugated goat anti-rabbit IgG (1:1000, Cat: ab150077, Abcam), as well as secondary antibody (1:200, Cat: AS073, ABclonal). For vascular statistical evaluation, eight randomly selected 20× field images per group were analysed to quantify both the number and area of blood vessels using ImageJ software 1.53. Vascular maturity was assessed by measuring the short diameter of each vessel. The mouse organs, including heart, lung, kidney, spleen and liver, were under healthy conditions after the in vivo study of scaffold transplantation (Supplementary Fig. 18).

### Statistical analysis

All the experiments were performed no less than three times. All data were presented as mean ± standard deviation. Statistical significance was determined by a two-tailed *t*-test using the PSPP 2.0 software. Prior to analysis, data normality was assessed using the Shapiro–Wilk test to ensure the validity of the *t*-test. For datasets that did not meet the normality assumption, non-parametric analysis using the two-tailed Mann–Whitney U test was applied. Differences were considered statistically significant at *p < 0.05, **p < 0.01 and ***p < 0.001, while *p* > 0.05 was considered no significant difference.

### Reporting summary

Further information on research design is available in the Nature Portfolio Reporting Summary linked to this article.

## Supplementary information

Supplementary Information

Reporting Summary

Transparent Peer Review file

## Source data

Source data

## Data Availability

The data supporting the findings of this study are available in the paper and the Supplementary Information. Source data are provided together with this paper. Source data is available for Figs. 1–6, and Supplementary Figs. 2–4, 8, 11, 13, 14 and 19 in the associated source data file. Source data are provided with this paper. The STL files used to generate the scaffolds are available via 10.6084/m9.figshare.31970064.
