# Supplementary material for: 3D triply periodic minimal surface gyroid hydrogel scaffolds for soft tissue engineering
Source: Nat Commun. 2026 May 19;17:7283. doi: 10.1038/s41467-026-73452-y (PMC13402795; doi:10.1038/s41467-026-73452-y)
Supplement: Supplementary file 1 — Supplementary Information [file 41467_2026_73452_MOESM1_ESM.pdf]

Supplementary Information

3D triply periodic minimal surface gyroid hydrogel scaffolds for soft tissue engineering

This file contains additional figures and results that support the findings stated in the main manuscript.

Supplementary Note 1 | Design and generation of gyroid geometry

The gyroid scaffold geometry was created using the MSLattice software (1) developed for generating uniform and functionally graded lattice structures based on triply periodic minimal surfaces (TPMS). The software employs a level-set approximation approach to describe the gyroid minimal surface that is defined mathematically using the following trigonometric equation:

$$\phi(x,y,z) = cos(\omega x)sin(\omega y) + cos(\omega y)sin(\omega z) + cos(\omega z)sin(\omega x) = c \quad (1)$$

where the function $\phi(x,y,z)$ is an iso-surface evaluated at the level offset value c and $c = 0$ stands for the zero-level offset of the function (the true minimal surface); $\omega = \frac{2\pi}{L}$ and L is the length of the unit cell. The software uses implicit surface modelling to represent, manipulate, and propagate unit cells within a given volume. By offsetting the minimal surface along and against its normal direction to create a double surface, the volume is generated by solving $-c \leq \phi(x,y,z) \leq c$. By providing the following input parameters: relative density (0–100%), size of the unit cell, sample length, sample width, sample height, and mesh density points, the software can generate both sheet (surface) and solid networks. In this study, all gyroid structures were created using sheet networks.

Supplementary Note 2 | Design and generation of lattice scaffold

To provide a meaningful comparison with the optimised gyroid scaffold, we designed a lattice scaffold control with closely matched volume fraction, cell parameters (cell density and total cell number), and bulk mechanical properties. Initially, we designed a lattice scaffold with identical cell parameters (density and cell number), scaffold size (7 × 7 mm), volume fraction (20%) and wall thickness (Supplementary Fig. 4a). However, fabrication of this lattice scaffold was limited by printability and structural stability: the printed walls deformed substantially and the construct collapsed during handling. To improve mechanical integrity, we increased the wall thickness to raise the compressive modulus. To maintain the same volume fraction and total cell number, the scaffold footprint was unavoidably reduced. The resulting lattice scaffold (5 × 5 mm) with a wall thickness of approximately ~350 μm achieved a comparable compressional modulus (~12.0 kPa) and maintained the structural stability required for handling and implantation (Supplementary Fig. 4b). We next varied the number of unit cells to assess its effects on mechanical properties and diffusion. Increasing the unit cell number from 3 × 3 to 5 × 5 produced a similar modulus (Supplementary Fig. 4c–d) and a modest increase in diffusion (Supplementary Fig. 4e–f). However, the associated reduction in wall thickness for the 5 × 5 design (to ~280 μm) remained prone to deformation during handling and in vitro culture. Therefore, the 5 × 5 mm lattice scaffold with a 3 × 3 unit-cell design was selected as the control condition.

Supplementary Figures

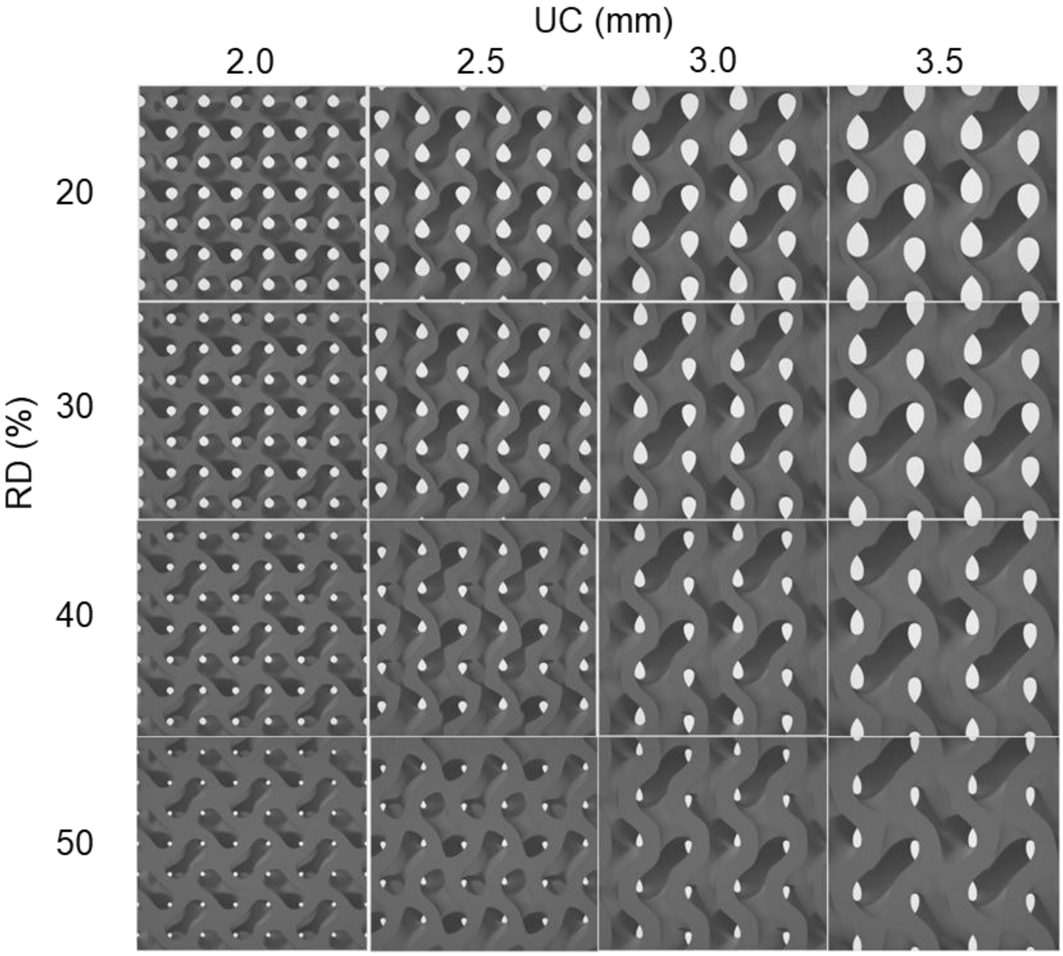

Supplementary Fig. 1 Geometries of the gyroid structure with varying relative density (RD) and the size of the unit cell (UC).

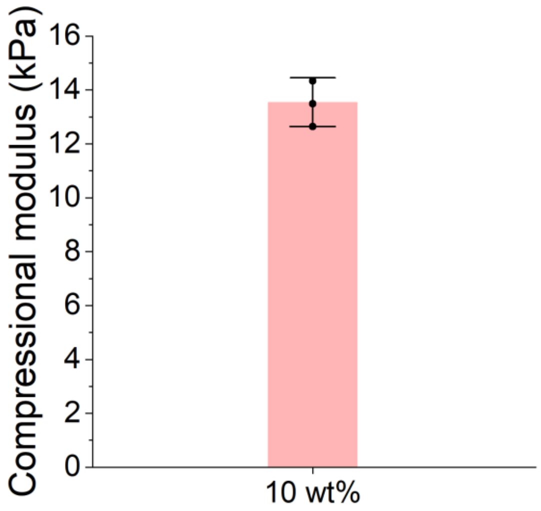

Supplementary Fig. 2 Compressional modulus of 10 wt% GelMA Hydrogel material (n = 3 independent experiments). Data are presented as mean ± standard deviation. Source data are provided as a source data file.

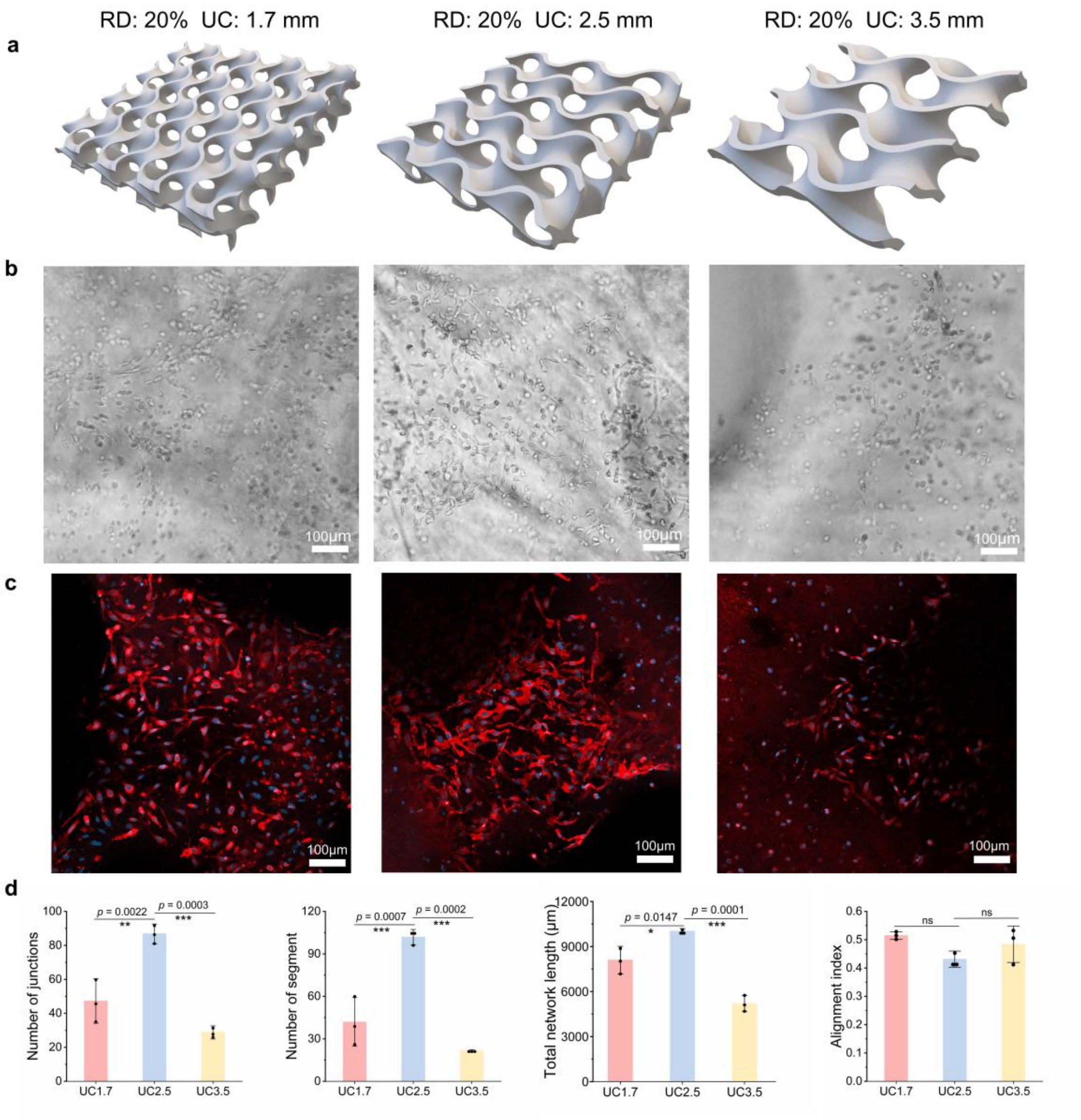

Supplementary Fig. 3 The effect of curvatures on capillary network formation. **a** Schematic diagrams showing the gyroid scaffolds with different curvatures. RD indicate the relative density and UC is the unit cell. **b** Representative bright-field images and **c** CD31 immunofluorescence staining of endothelial cells after 5-day culture, from three independent experiments. **d** Capillary network formation statistics including the number of junctions, the number of segments, the total network length, and the alignment index (n = 3 independent experiments). Data in **d** are presented as mean ± standard deviation. Statistical significance was determined by two-tailed t-test following the Shapiro-Wilk normality test: *$p < 0.05$, **$p < 0.01$, ***$p < 0.001$, and $p > 0.05$ (not significant). Source data are provided as a source data file.

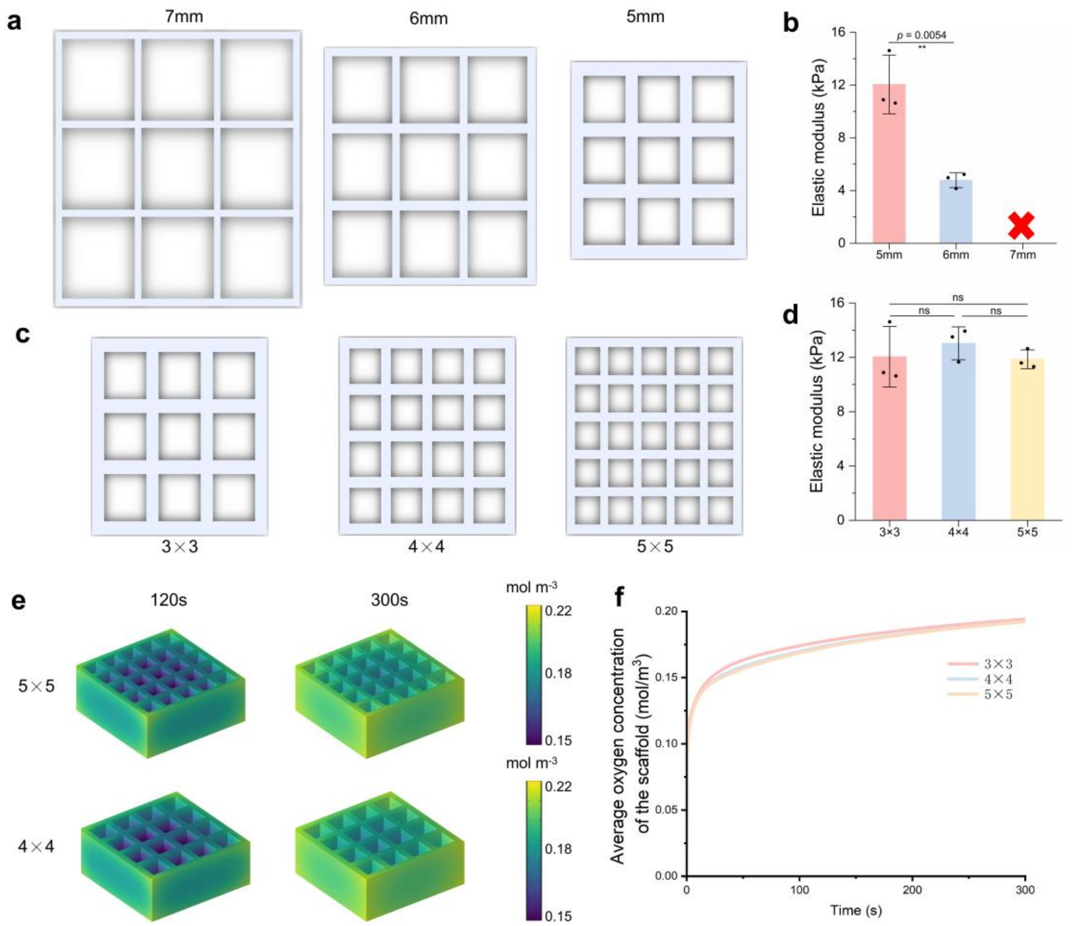

Supplementary Fig. 4 Design process of lattice scaffolds. **a** Schematic diagrams showing lattice scaffolds with different sizes and identical volume fraction. **b** Elastic modulus of **a** (n = 3 independent experiments). **c** Schematic illustration of lattice scaffolds with different number of unit cell. The volume fraction and size (5 × 5 mm) are identical. **d** Elastic modulus of **c** (n = 3 independent experiments). Note that the modulus data for the scaffold with a size of 5 × 5 mm and a unit cell number of 3 × 3 was re-used for the convenience of comparison. **e** Modelled oxygen concentration in lattice scaffolds with different number of unit cell. **f** Temporal variation of the averaged oxygen concentration in the scaffolds. Data in **b** and **d** are presented as mean ± standard deviation. Statistical significance was determined by two-tailed t-test following the Shapiro-Wilk normality test: *$p < 0.05$, **$p < 0.01$, ***$p < 0.001$, and $p > 0.05$ (not significant). Source data are provided as a source data file.

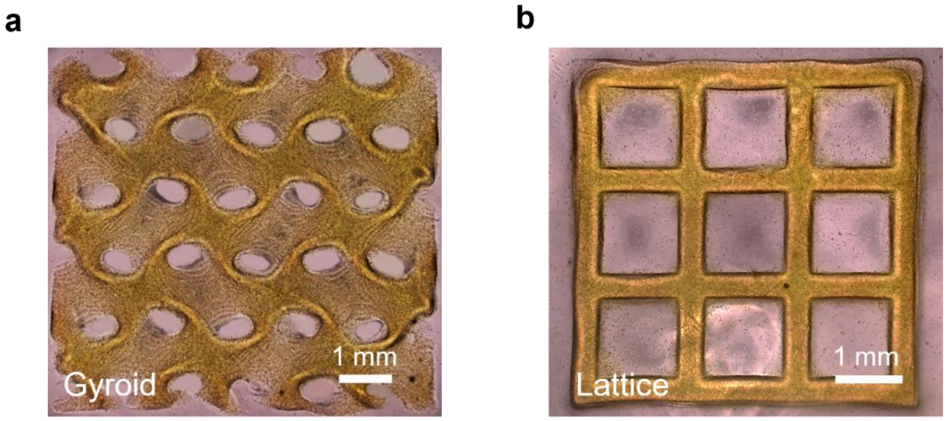

Supplementary Fig. 5 Representative images of printed **a** gyroid and **b** lattice scaffolds from three independent experiments. The 10 wt% GelMA hydrogel bioink contains HUVECs at a high density of 1×10^7 cells ml^{-1}.

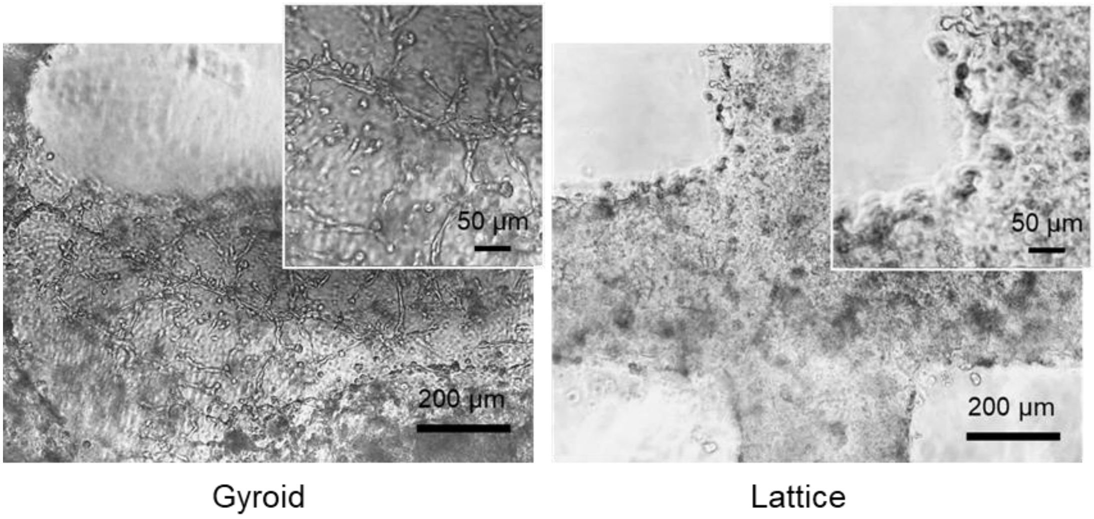

Supplementary Fig. 6 Representative bright field images showing cell spreading and morphology in the scaffolds laden with HUVEC on day 2, from three independent experiments.

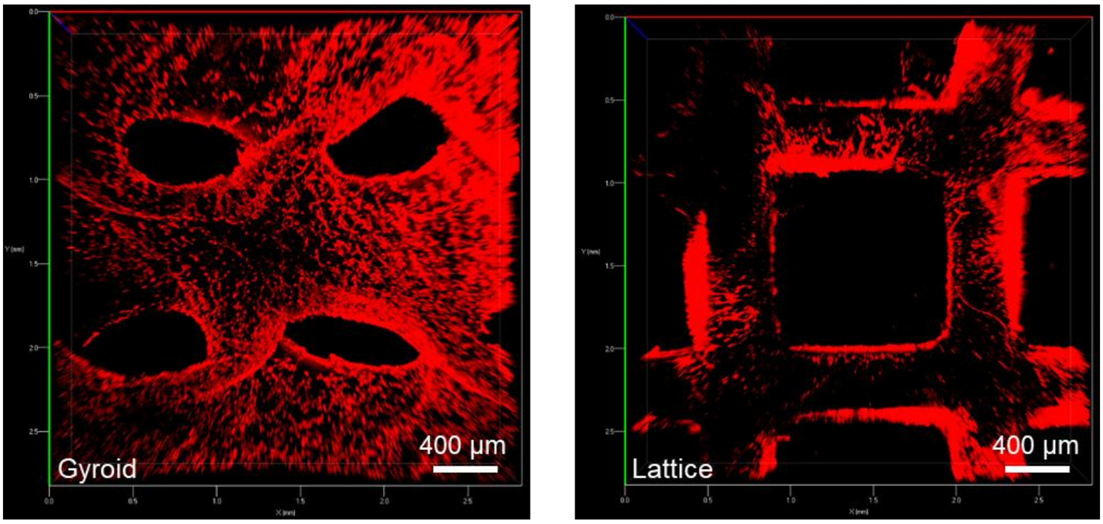

Supplementary Fig. 7 Representative CD31 immunofluorescence staining showing the endothelial network formation after a 5-day culture in gyroid and lattice scaffolds, from three independent experiments.

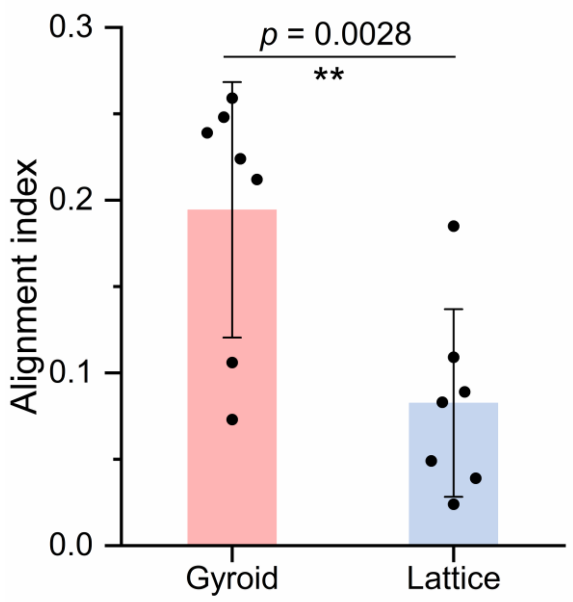

Supplementary Fig. 8 Alignment index of capillary network formation in the scaffold (*n* = 7 independent experiments). Data are presented as mean ± standard deviation. Statistical significance was determined by two-tailed t-test following the Shapiro-Wilk normality test: *$p < 0.05$, **$p < 0.01$, ***$p < 0.001$, and $p > 0.05$ (not significant). Source data are provided as a source data file.

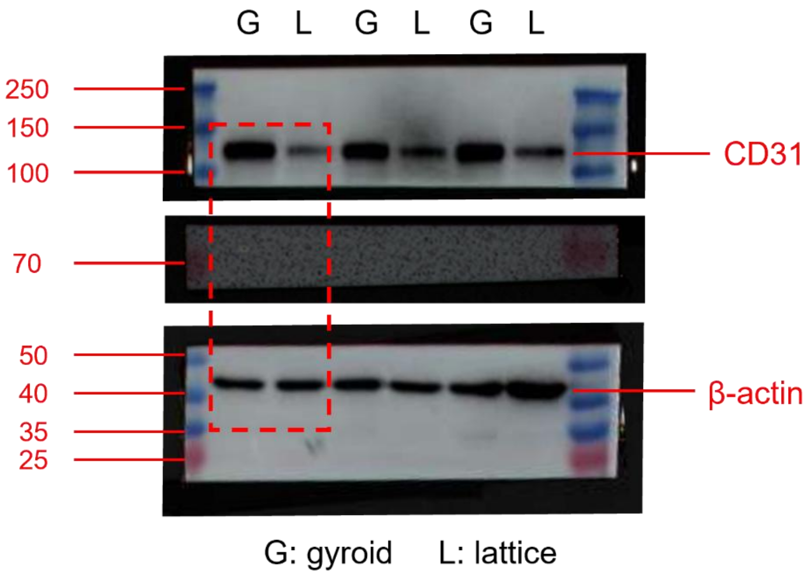

Supplementary Fig. 9 Raw bands of western blot analysis for CD31 protein expression in Fig. 2k. The molecular weight is expressed in kilodaltons (kDa). The dashed rectangular box highlights the representative results shown in the main manuscript.

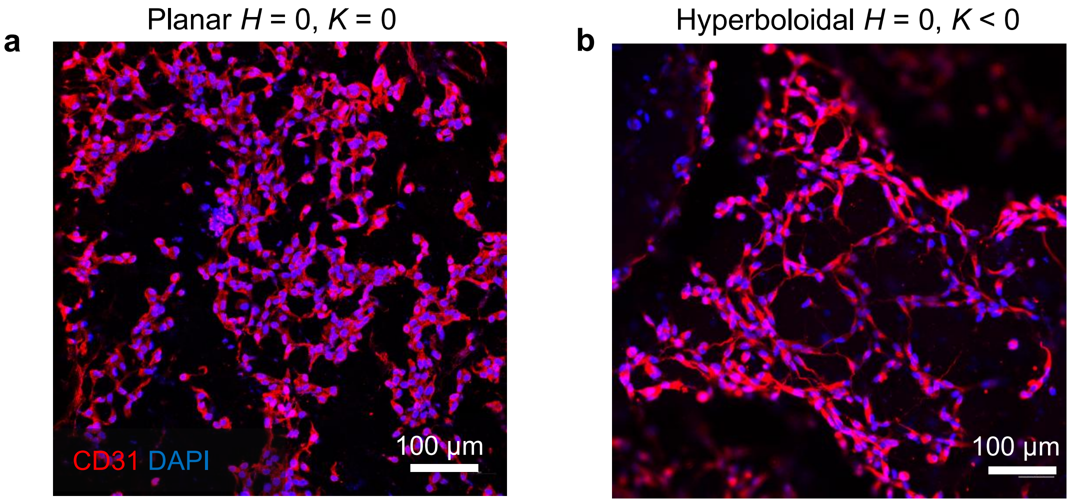

Supplementary Fig. 10 Representative images of CD31 expression (red, CD31; blue, DAPI) of HUVEC in different scaffolds featuring different 3D curvatures, including **a** planar and **b** hyperboloidal, from three independent experiments. *H* is the mean curvature, while *K* indicates the Gaussian curvature. They are defined by $(k_1 + k_2)/2$ and $k_1 \times k_2$, respectively, where k_1 and k_2 are the principal curvatures at a point on the curved surface.

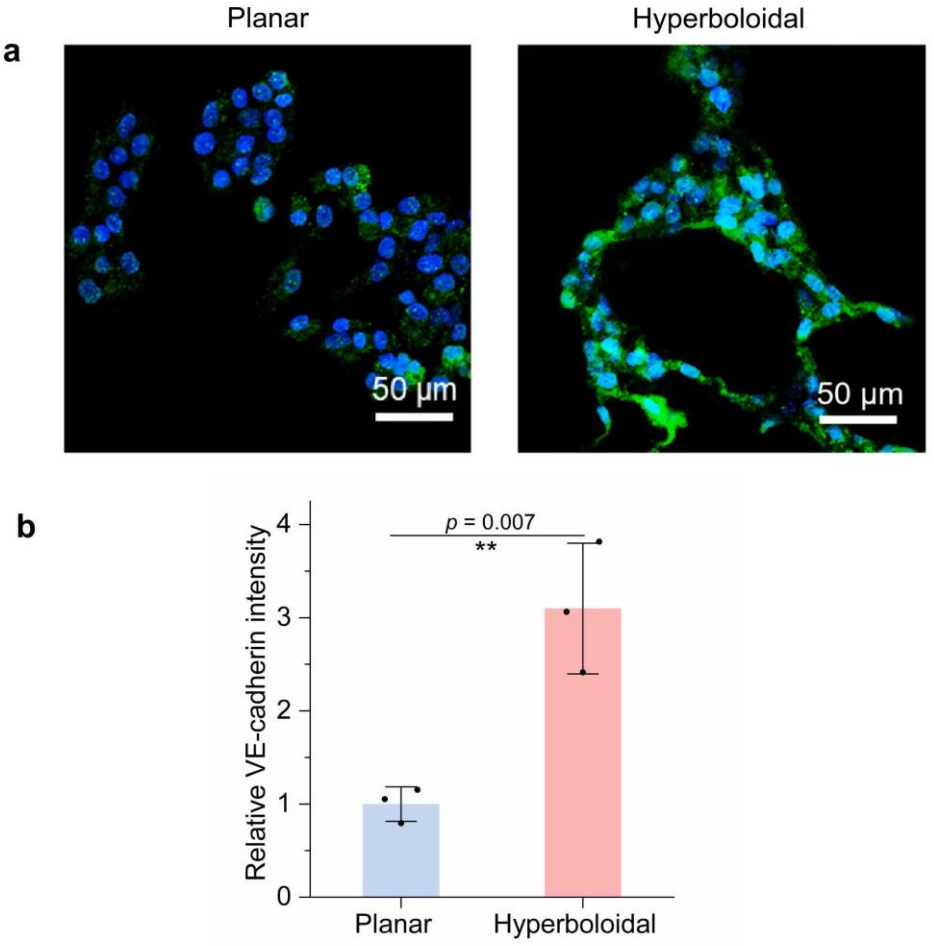

Supplementary Fig. 11 Comparison of VE-cadherin expression levels. **a** Representative immunofluorescence staining images showing VE-cadherin expression of endothelial cells in planar and gyroid geometries, from three independent experiments. **b** Statistical analysis corresponding to the staining images (n = 3 independent experiments). Data in **b** are presented as mean ± standard deviation. Statistical significance was determined by two-tailed t-test following the Shapiro-Wilk normality test: *$p < 0.05$, **$p < 0.01$, ***$p < 0.001$, and $p > 0.05$ (not significant). Source data are provided as a source data file.

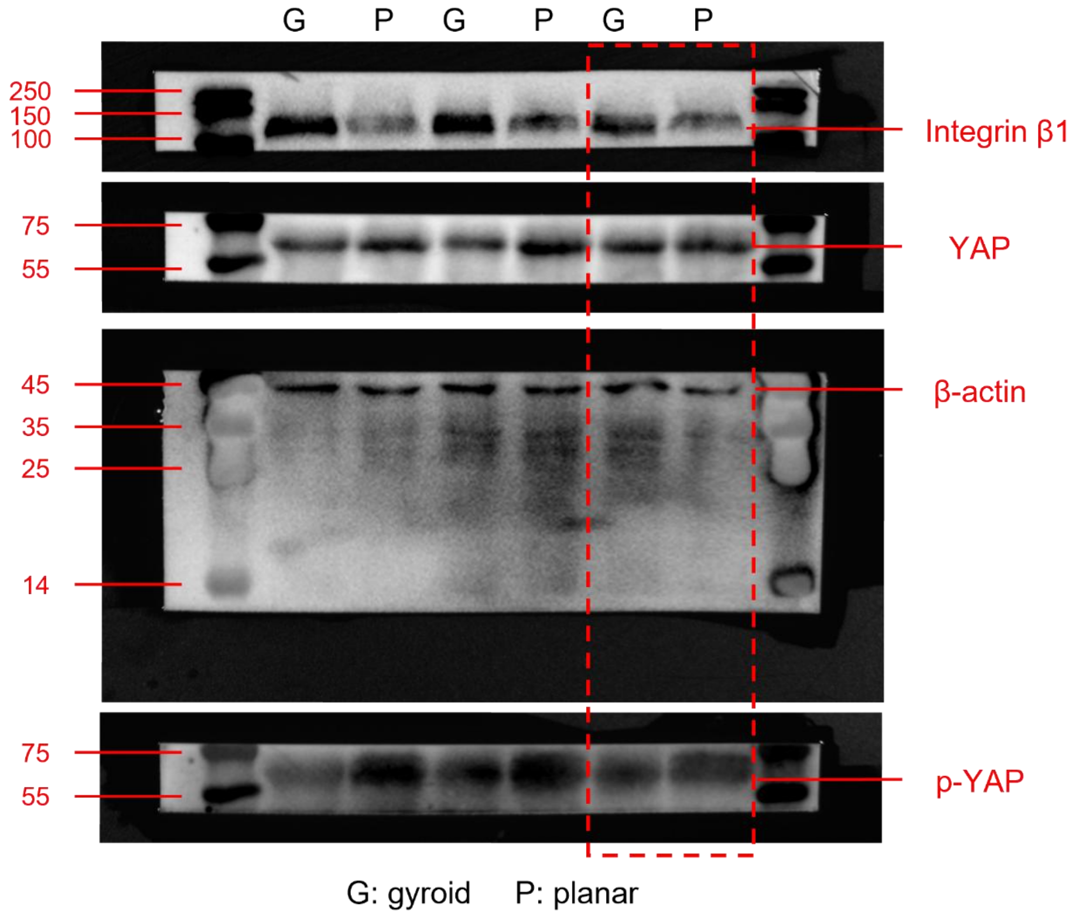

Supplementary Fig. 12 Raw bands of western blot analysis for integrin β1, Yes-associated protein (YAP) and phosphorylated YAP (p-YAP) proteins expression in Fig. 3j. The molecular weight is expressed in kilodaltons (kDa). The dashed rectangular box highlights the representative results shown in the main manuscript.

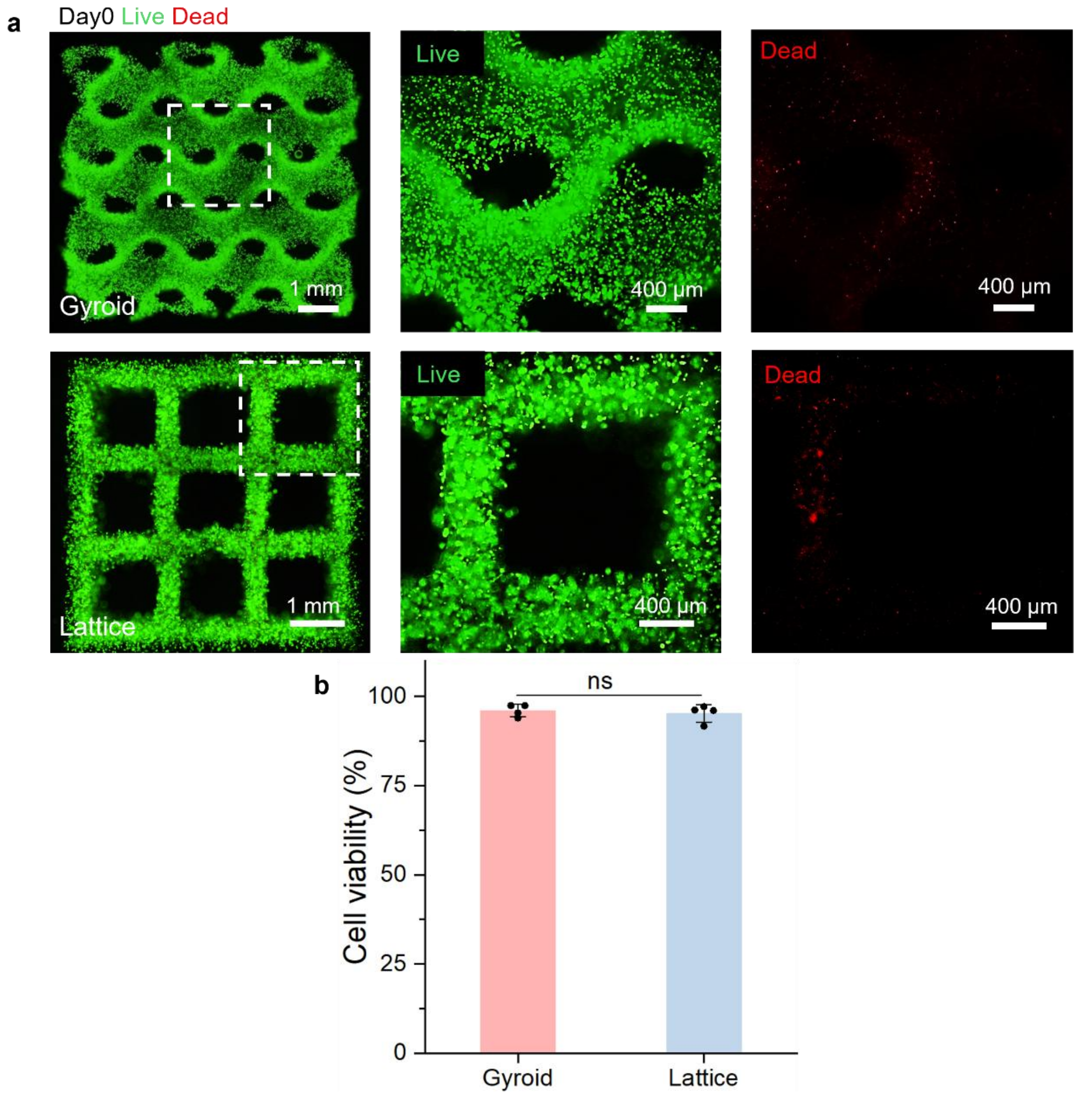

Supplementary Fig. 13 Live/dead assay of the cell-laden scaffolds after printing using a high cell density containing HepG2 (1.6×10^7 cells mL^{-1}) and HUVEC (4×10^6 cells mL^{-1}). **a** Representative confocal images showing the Live/dead assay on day 0 from three independent experiments**.** The cell viability and geometric fidelity were preserved. **b** Quantitative comparison of cell viability on day 0 (n = 3 independent experiments). Data in **b** are presented as mean ± standard deviation. Statistical significance was determined by two-tailed t-test following the Shapiro-Wilk normality test: *$p < 0.05$, **$p < 0.01$, ***$p < 0.001$, and $p > 0.05$ (not significant). Source data are provided as a source data file.

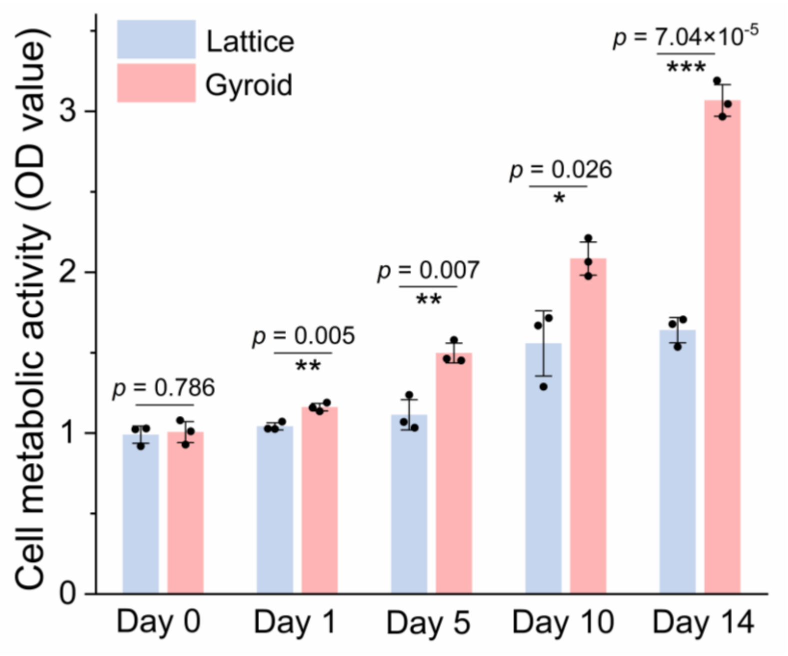

Supplementary Fig. 14 CCK-8 assay showing the cell metabolic activity in gyroid and lattice scaffolds (n = 3 independent experiments). Data are presented as mean ± standard deviation. Statistical significance was determined by two-tailed t-test following the Shapiro-Wilk normality test: *p < 0.05, **p < 0.01, ***p < 0.001, and p > 0.05 (not significant). Source data are provided as a source data file.

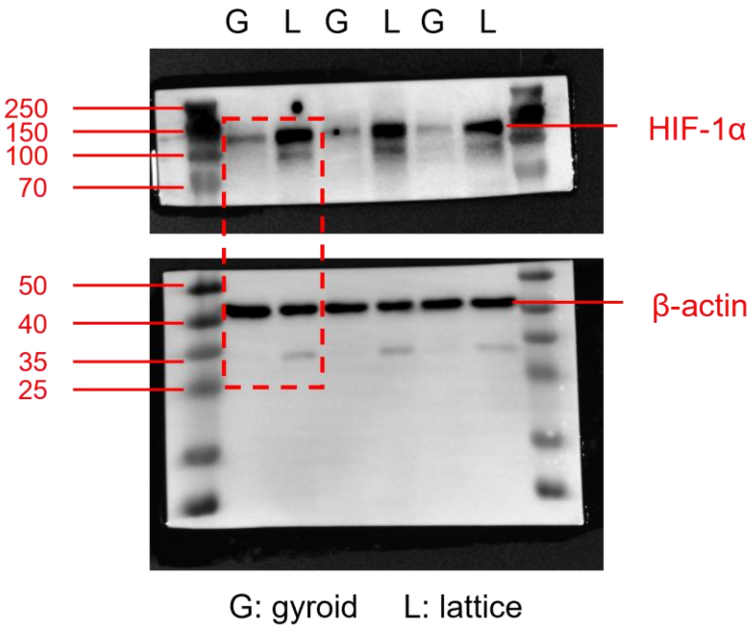

Supplementary Fig. 15 Raw bands of western blot analysis for HIF-1α protein expression in Fig. 4f. The molecular weight is expressed in kilodaltons (kDa). The dashed rectangular box highlights the representative results shown in the main manuscript.

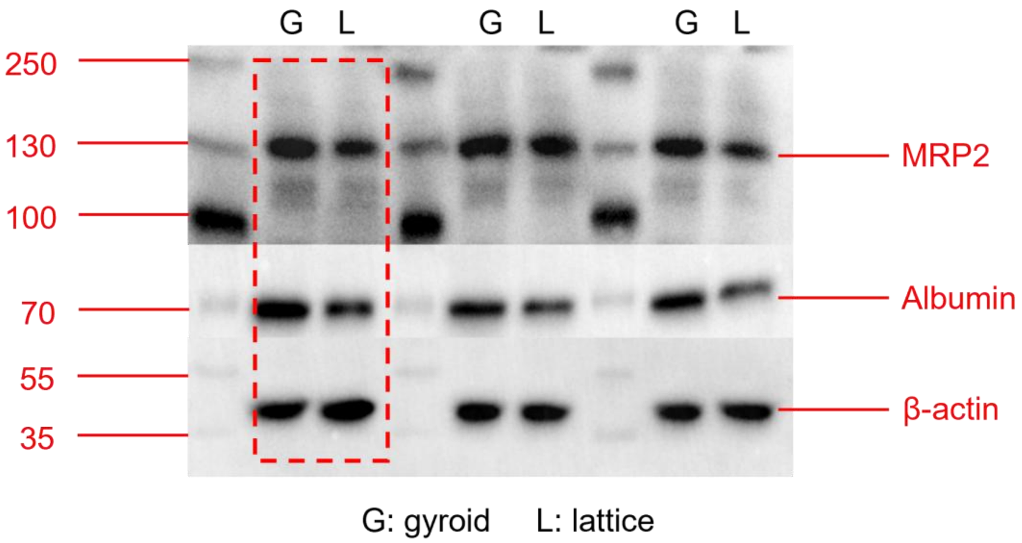

Supplementary Fig. 16 Raw bands of western blot analysis for MPR2 and Albumin proteins expression in Fig. 5f. The molecular weight is expressed in kilodaltons (kDa). The dashed rectangular box highlights the representative results shown in the main manuscript.

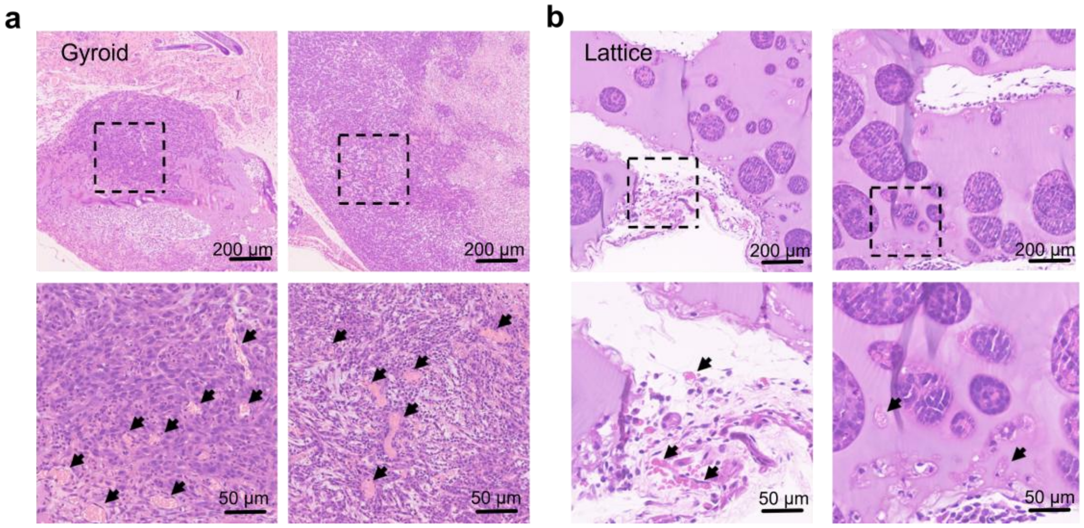

Supplementary Fig. 17 Representative immunohistochemistry images of tumour tissues generated by **a** gyroid and **b** lattice scaffolds, from four independent experiments. Black arrows indicate the location of vessels. The dashed box indicates the magnified area.

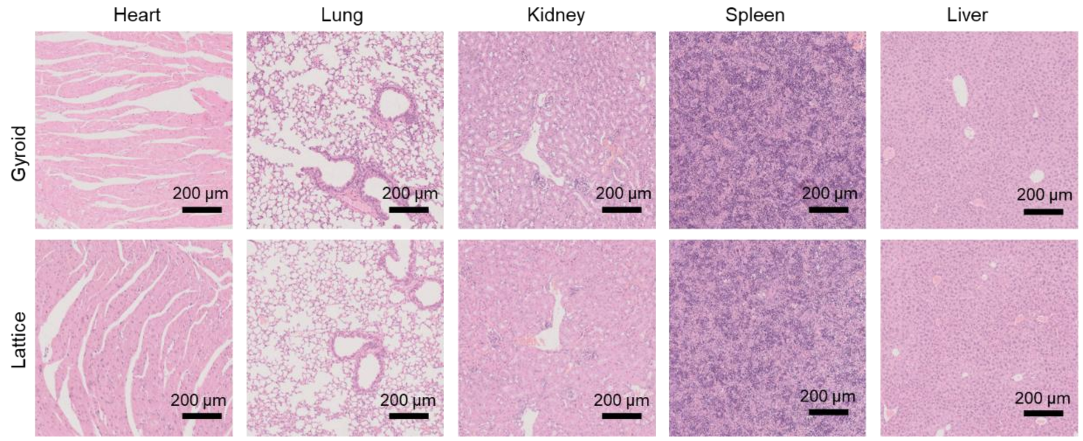

Supplementary Fig. 18 Representative immunohistochemistry images of mice organs, including heart, lung, kidney, spleen and liver, from four independent experiments. The host organs were under healthy conditions after the *in vivo* study of scaffold transplantation.

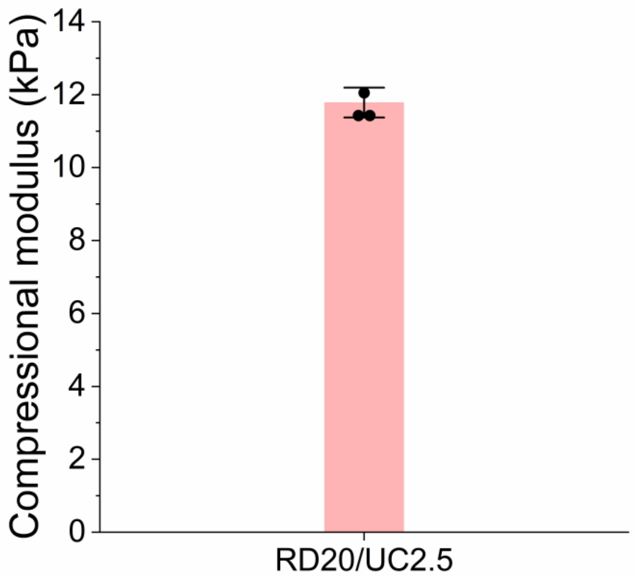

Supplementary Fig. 19 Compressive modulus of the gyroid scaffold with a relative density of 20% and unit cell size of 2.5 mm (*n* = 3 independent experiments). The three samples were fabricated from independent experiments, including separate bioink preparation and printing, to assess batch-to-batch reproducibility. Data are presented as mean ± standard deviation. Source data are provided as a source data file.

Supplementary Table 1 Geometric parameters of the gyroid scaffolds for curvature analysis

Size (mm) L×W×H	Unit cell (mm)	Relative density (%)	Mean Gaussian curvature (mm^{-2})
7×7×1.5	1.7	20%	-5.00
	2.5		-2.30
	3.5		-1.25

Supplementary Table 2 Comparison between lattice and gyroid scaffolds

Feature	Lattice scaffold	Gyroid scaffold
Geometry	Planar strut-based	Continuous TPMS / hyperboloidal
Fabrication stability	Lower at thin wall thickness	Higher structural stability
Printing fidelity	More prone to collapse/deformation	Better fidelity in soft GelMA
Curvature cue	Limited / planar	Continuous 3D curvature
Endothelial response	Weaker morphogenesis/networking	Enhanced filopodia and network formation
In vivo outcome	Lower tumour maturation/vascularisation	Improved tumour tissue formation/vascularisation

Supplementary Table 3 Primers sequences in the qPCR study

**Target gene**	**Forward (5'-3')**	**Reverse (5'-3')**
CD31	ACGTGCAGTACACGGAAGTT	GGAGCCTTCCGTTCTAGAGT
ALB	CAAAGGCAATCAACACC	TCGGCTTATTCCAGG
AFP	TGTTTCATCCACCACC	GAGCGGCTGACATTAT
β-actin	CACCATTGGCAATGAGCGGTTC	AGGTCTTTGCGGATGTCCACGT

Reference:

1. Al-Ketan O, Abu Al-Rub RK. MSLattice: A free software for generating uniform and graded lattices based on triply periodic minimal surfaces. Mat Design Process Comm., 3(6): e205 (2021).
