# Supplementary material for: 3D triply periodic minimal surface gyroid hydrogel scaffolds for soft tissue engineering
Source: Nat Commun. 2026 May 19;17:7283. doi: 10.1038/s41467-026-73452-y (PMC13402795; doi:10.1038/s41467-026-73452-y)
Supplement: Supplementary file 2 — Reporting Summary [file 41467_2026_73452_MOESM2_ESM.pdf]

nature portfolio

Corresponding author(s): Feng Chen

Last updated by author(s): May 1, 2026

Reporting Summary

Nature Portfolio wishes to improve the reproducibility of the work that we publish. This form provides structure for consistency and transparency in reporting. For further information on Nature Portfolio policies, see our Editorial Policies and the Editorial Policy Checklist.

Statistics

For all statistical analyses, confirm that the following items are present in the figure legend, table legend, main text, or Methods section.

n/a	Confirmed	
☐	☒	The exact sample size (*n*) for each experimental group/condition, given as a discrete number and unit of measurement
☐	☒	A statement on whether measurements were taken from distinct samples or whether the same sample was measured repeatedly
☐	☒	The statistical test(s) used AND whether they are one- or two-sided<br>*Only common tests should be described solely by name; describe more complex techniques in the Methods section.*
☒	☐	A description of all covariates tested
☐	☒	A description of any assumptions or corrections, such as tests of normality and adjustment for multiple comparisons
☐	☒	A full description of the statistical parameters including central tendency (e.g. means) or other basic estimates (e.g. regression coefficient) AND variation (e.g. standard deviation) or associated estimates of uncertainty (e.g. confidence intervals)
☐	☒	For null hypothesis testing, the test statistic (e.g. *F*, *t*, *r*) with confidence intervals, effect sizes, degrees of freedom and *P* value noted<br>*Give P values as exact values whenever suitable.*
☒	☐	For Bayesian analysis, information on the choice of priors and Markov chain Monte Carlo settings
☒	☐	For hierarchical and complex designs, identification of the appropriate level for tests and full reporting of outcomes
☒	☐	Estimates of effect sizes (e.g. Cohen's *d*, Pearson's *r*), indicating how they were calculated

Our web collection on statistics for biologists contains articles on many of the points above.

Software and code

Policy information about availability of computer code

Data collection	Compression modulus data were collected using Trapezium Lite X software.<br>Microscopy images were obtained using the Dino-Lite microscope (Dunwell Tech, Inc., USA) with the DinoCapture 2.0 software.<br>Confocal images for were captured using ZEN 2012 software.<br>Oxygen diffusion modelling was conducted using COMSOL Multiphysics software 6.<br>Aplegen imaging system was controlled by Omega Lum G Capture Software.<br>Applied Biosystems 7500 system was controlled by HMR software 2.0.
Data analysis	The 3D model to be printed was sliced by using a BMF 3D slicer software (1.6.2).<br>Origin 2018 software was used for plotting.<br>Significant differences between the means of parameters were calculated using the PSPP 2.0 software.<br>The Angiogenesis Analyze Plugin in ImageJ was employed to quantify vascular networks using a default setting.<br>ImageJ software 1.53 was used to analyse protein expression levels of WB analysis and relative fluorescence intensity analysis.<br>Gyroid scaffolds were generated using MSLattice software.<br>The lattice scaffold was designed with Computer-Aided Design (CAD) 2021 software.

For manuscripts utilizing custom algorithms or software that are central to the research but not yet described in published literature, software must be made available to editors and reviewers. We strongly encourage code deposition in a community repository (e.g. GitHub). See the Nature Portfolio guidelines for submitting code & software for further information.

Data

Policy information about availability of data

All manuscripts must include a data availability statement. This statement should provide the following information, where applicable:

- Accession codes, unique identifiers, or web links for publicly available datasets
- A description of any restrictions on data availability
- For clinical datasets or third party data, please ensure that the statement adheres to our policy

The data supporting the findings of this study are available in the paper and the Supplementary Information. Source data are provided together with this paper. Source data is available for Fig.1–6, and Supplementary Fig.2–4, 8, 11, 13, 14 and 19 in the associated source data file.

Research involving human participants, their data, or biological material

Policy information about studies with human participants or human data. See also policy information about sex, gender (identity/presentation), and sexual orientation and race, ethnicity and racism.

Reporting on sex and gender	Human research participants were not involved in this study.
Reporting on race, ethnicity, or other socially relevant groupings	Human research participants were not involved in this study.
Population characteristics	Human research participants were not involved in this study.
Recruitment	Human research participants were not involved in this study.
Ethics oversight	Human research participants were not involved in this study.

Note that full information on the approval of the study protocol must also be provided in the manuscript.

Field-specific reporting

Please select the one below that is the best fit for your research. If you are not sure, read the appropriate sections before making your selection.

☒ Life sciences ☐ Behavioural & social sciences ☐ Ecological, evolutionary & environmental sciences

For a reference copy of the document with all sections, see nature.com/documents/nr-reporting-summary-flat.pdf

Life sciences study design

All studies must disclose on these points even when the disclosure is negative.

Sample size	All experiments were conducted with at least three biological replicates. The sample sizes are described in the figure legends.
Data exclusions	No data were excluded from the analysis.
Replication	All the experiment replication was not less than three. All attempts were successful, except that 90%-100% tumour formation was achieved, depending on the physiological conditions of animals.
Randomization	The cells cultured under the same condition were mixed homogeneously, and randomly divided into three groups for further study. Mice housed under identical conditions were randomly assigned to different experimental groups.
Blinding	The cell samples were allocated to hydrogel materials in a single-blind approach. The persons performing sample preparation were aware of the identity of hydrogel materials, but unaware of the identity of cell groups.

Reporting for specific materials, systems and methods

We require information from authors about some types of materials, experimental systems and methods used in many studies. Here, indicate whether each material, system or method listed is relevant to your study. If you are not sure if a list item applies to your research, read the appropriate section before selecting a response.

Materials & experimental systems

n/a	Involved in the study	
☐	☒	Antibodies
☐	☐	Eukaryotic cell lines
☒	☐	Palaeontology and archaeology
☐	☒	Animals and other organisms
☒	☐	Clinical data
☒	☐	Dual use research of concern
☒	☐	Plants

Methods

n/a	Involved in the study	
☒	☐	ChIP-seq
☒	☐	Flow cytometry
☒	☐	MRI-based neuroimaging

Antibodies

Antibodies used

Anti-CD31 (1:100, Cat: 66065-2-Ig, clone: 3F8E2, Proteintech) for immunfluorescence and immunohistochemical

Anti-VE-cadherin (1:200, Cat: D87F2, Cell signaling) for immunfluorescence

Anti-albumin (1:500, Cat: ab207327, clone: EPR20195, Abcam) for immunfluorescence and immunohistochemical

Alexa Fluor 488 phalloidin (1:1000, Cat: AC18L032, Life-iLab) for immunfluorescence

Anti-HIF-1α (1:200, Cat: ab51608, clone: EP1215Y, Abcam) for western blotting

Anti-CD31 (1:10000, Cat: ab76533, clone: EPR3094, Abcam) for western blotting

Anti-MRP2 (1:1000, Cat: ab172630, clone: EPR10998, Abcam) for western blotting

Anti-albumin (1:2000, Cat: ab207327, clone: EPR20195, Abcam) for western blotting

Anti-β-actin (1:1000, Cat: 4970, Cell Signaling) for western blotting

Anti-YAP1 (1:5000, Cat: ab52771, clone: EP1674Y, Abcam) for western blotting and immunfluorescence

Anti-YAP1(phospho S127) (1:10000, Cat: ab76252, clone: EP1675Y, Abcam) for western blotting

Anti-Integrin-β1 (1:10000, Cat: A23497, clone: ARC52470, ABclonal) for western blotting and immunfluorescence

ABflo 594-conjugated Goat Anti-Mouse IgG (H+L) secondary antibody (1:200, Cat: AS054, ABclonal) for immunfluorescence

Alexa Fluor 488 Goat Anti-Rabbit IgG (1:1000, Cat: ab150077, Abcam) for immunfluorescence

Goat anti-rabbit IgG (H+L) Cross-Absorbed secondary antibody (1:2000, Cat: ab6721, Abcam) for western blotting

Secondary antibody (1:200, Cat: AS073, ABclonal) for immunfluorescence

Validation

All antibodies used in this study were commercially obtained and validated by the manufacturers using western blots, IF and IHC:

anti-CD31 (1:100, 66065-2-Ig, clone: 3F8E2, Proteintech) for immunfluorescence and immunohistochemical (https://www.ptglab.com/products/CD31-Antibody-66065-2-Ig.htm?srsltid=AfmBOoofl2gnxD1s_vOBvaF2xrmWG7bd48mHjoz9hjTTFcHwJKfyY6zd)

anti-VE-cadherin (1:200, D87F2, Cell signaling) for immunfluorescence (https://www.cellsignal.com/products/primary-antibodies/ve-cadherin-d87f2-rabbit-monoclonal-antibody/2500)

anti-albumin (1:500, ab207327, cloned EPR20195, Abcam) for immunfluorescence and immunohistochemical (https://www.abcam.cn/products/primary-antibodies/albumin-antibody-epr20195-ab207327)

Alexa Fluor 488 phalloidin (1:1000, AC18L032, Life-iLab) for immunfluorescence (https://www.life-ilab.com/products/show-309.html)

anti-HIF-1α (1:200, ab51608, cloned EP1215Y, Abcam) for western blotting (https://www.abcam.cn/products/primary-antibodies/hif-1-alpha-antibody-ep1215y-ab51608)

anti-CD31 (1:10000, ab76533, cloned EPR3094, Abcam) for western blotting (https://www.abcam.cn/products/primary-antibodies/cd31-antibody-epr3094-ab76533)

anti-MRP2 (1:1000, ab172630, cloned EPR10998, Abcam) for western blotting (https://www.abcam.cn/products/primary-antibodies/mrp2-antibody-epr10998-ab172630)

anti-albumin (1:2000, ab207327, cloned EPR20195, Abcam) for western blotting (https://www.abcam.cn/products/primary-antibodies/albumin-antibody-epr20195-ab207327)

anti-β-actin (1:1000, 4970, Cell Signaling) for western blotting (https://www.cellsignal.com/products/primary-antibodies/beta-actin-13e5-rabbit-monoclonal-antibody/4970)

anti-YAP1 (1:5000, ab52771, cloned EP1674Y, Abcam) for western blotting and immunfluorescence (https://www.abcam.cn/products/primary-antibodies/yap1-antibody-ep1674y-ab52771)

anti-YAP1(phospho S127) (1:10000, ab76252, cloned EP1675Y, Abcam) for western blotting (https://www.abcam.cn/products/primary-antibodies/yap1-phospho-s127-antibody-ep1675y-ab76252)

anti-Integrin-β1 (1:10000, A23497, ABclonal) for western blotting and immunfluorescence (https://abclonal.com.cn/catalog/A23497)

ABflo 594-conjugated Goat Anti-Mouse IgG (H+L) secondary antibody (1:200, AS054, ABclonal) for immunfluorescence (https://abclonal.com.cn/catalog/AS054)

Alexa Fluor 488 Goat Anti-Rabbit IgG (1:1000, ab150077, Abcam) for immunfluorescence (https://www.abcam.cn/products/secondary-antibodies/goat-rabbit-igg-h-l-alexa-fluor-488-ab150077)

goat anti-rabbit IgG (H+L) Cross-Absorbed secondary antibody (1:2000, ab6721, Abcam) for western blotting (https://www.abcam.cn/products/secondary-antibodies/goat-rabbit-igg-h-l-hrp-ab6721)

secondary antibody (1:200, AS073, ABclonal) for immunfluorescence (https://abclonal.com.cn/catalog/AS073)

The experiments also validated the antibodies with microscopy and western blot analysis that are present in the manuscript and Supplementary Information. For Western blot analysis, bands were detected at the expected molecular weights, and no significant non-specific bands were observed. For immunofluorescence and immunohistochemical staining, the observed signal localisation was consistent with the known subcellular distribution of the target proteins.

Eukaryotic cell lines

Policy information about cell lines and Sex and Gender in Research

Cell line source(s)	HepG2 (Cat No.: CL-0103) cells were obtained from Procell Life Science & Technology Co., Ltd, China. HUVECs (Cat No.: SCSP-5535) were sourced from the National Collection of Authenticated Cell Cultures, China.
Authentication	The HUVECs were authenticated by CD31 expression via qPCR analysis. The HepG2 cells were authenticated by ALP and AFP expression via qPCR analysis. The primers are listed in Supplementary Table 3.
Mycoplasma contamination	The cell line has been tested negative for mycoplasma contamination.
Commonly misidentified lines (See ICLAC register)	Misidentified lines were not used.

Animals and other research organisms

Policy information about studies involving animals; ARRIVE guidelines recommended for reporting animal research, and Sex and Gender in Research

Laboratory animals	Male severely immunodeficient (NOD/ShiLtJGpt, NCG) mice, aged five weeks, were used for the experiments and were purchased from Chengdu Dossy Experimental Animals Co., Ltd.
Wild animals	This study did not involve wild animals.
Reporting on sex	Sex was not considered in this study.
Field-collected samples	This study did not involve any sample collected from the field.
Ethics oversight	The animal studies were approved by Central South University (approval number: CSU-2023-0377).

Note that full information on the approval of the study protocol must also be provided in the manuscript.

Plants

Seed stocks	None
Novel plant genotypes	None
Authentication	None
