# Supplementary material for: 3D triply periodic minimal surface gyroid hydrogel scaffolds for soft tissue engineering
Source: Nat Commun. 2026 May 19;17:7283. doi: 10.1038/s41467-026-73452-y (PMC13402795; doi:10.1038/s41467-026-73452-y)
Supplement: Supplementary file 3 — Transparent Peer Review file [file 41467_2026_73452_MOESM3_ESM.pdf]

nature portfolio

Peer Review File

3D triply periodic minimal surface gyroid hydrogel scaffolds for soft tissue engineering

Corresponding Author: Professor Feng Chen

This file contains all reviewer reports in order by version, followed by all author rebuttals in order by version.

Version 0:

Reviewer comments:

Reviewer #1

(Remarks to the Author)
This study proposes a triply periodic minimal surface (TPMS) scaffold with bioinspired and mechanically optimized potential, demonstrating significance in soft tissue engineering. While the conclusions are data-supported, addressing the following issues (particularly mechanistic validation and statistical rigor) will enhance manuscript quality. After major revisions, it is suitable for publication.

1. Although TPMS scaffolds have been explored for hard tissues (e.g., bone), their application in soft tissues remains underexplored. The authors appropriately cite prior work but should clarify the differences from recent studies (e.g., 10.1016/j.mtbio.2023.100667; 10.1073/pnas.2206684119).

2. The conclusion that 3D curvature promotes vascular self-assembly lacks molecular evidence (e.g., YAP/TAZ signaling). Incorporate Western blot (WB) or qPCR data to analyze curvature-sensitive genes (e.g., YAP/TAZ-related proteins) and clarify whether curvature regulates endothelial cell behavior through specific pathways.

3. While Cur-Na improves printing precision, its long-term biocompatibility requires comprehensive validation.

4. The scaffold’s modulus matches hepatic tissue (~12 kPa) under static conditions but neglects physiological dynamic loads (e.g., organ pulsation, shear stress).

5. Current evaluation focuses on structural metrics (CD31 expression, network length). Supplement with functional validation:
- Expression of tight junction proteins (e.g., VE-cadherin, ZO-1) to assess vascular maturity.
- Barrier functionality in co-culture models.

6. Define abbreviations upon first mention (e.g., *RD: radial distance; UC: umbilical cord*).

7. Correct grammatical errors (e.g., tense consistency, article usage).

Reviewer #2

(Remarks to the Author)
Comments for the Author

This manuscript presents a compelling study on the development of implantable, vascularized 3D tissue constructs using hydrogel-based triply periodic minimal surface (TPMS) architectures, specifically gyroid scaffolds. The authors successfully utilize a cell-laden, photocurable hydrogel and DLP-based 3D printing to fabricate high-resolution, curved structures with high cell density. The work investigates how 3D curvature modulates endothelial alignment and vascular morphogenesis through co-culture of HepG2 and HUVECs, followed by subcutaneous implantation. The study holds promise for liver tissue engineering and tumor modeling applications. However, several major revisions are necessary to enhance the clarity, reproducibility, and interpretability of the findings.

Major Comments

Gyroid Design Details
The gyroid architecture is a central feature of the study, yet the manuscript lacks essential information on its generation. Details such as the mathematical definition, generation algorithm, and design parameters (e.g., periodicity, amplitude, spacing) are not provided. This omission limits reproducibility. Including such information in the Supplementary Materials—along with representative STL files—would significantly improve the manuscript's utility and translational value.

Design Rationale for UC = 2.5 mm
The optimization of relative density (RD) and unit cell (UC) size is well supported by simulations and mechanical testing. However, the rationale for selecting UC = 2.5 mm remains unclear, especially given that Figure 1j suggests UC = 3.0 mm more closely aligns with physiological liver stiffness. Further clarification of this design choice beyond compressive strength would help justify the scaffold parameters in the context of hepatic applications.

Comparison with Prior Curvature-Based Models
The manuscript would benefit from a direct comparison with existing curvature-driven vascular models. Recent studies have demonstrated that curvature influences endothelial behavior and cancer cell dynamics. A critical comparison would contextualize the gyroid scaffold's novelty and relevance.
Suggested reference: Park et al., Nature Communications (2023).

Geometric Parameter Matching in Scaffold Comparison
When comparing gyroid and lattice scaffolds to assess curvature-specific effects, it is crucial to control for confounding factors such as volume fraction and wall thickness. Without such controls, observed cellular differences may arise from structural heterogeneity rather than curvature per se. Ensuring matched mechanical and geometric parameters would enhance the validity of biological comparisons.
Suggested reference: Kim et al., Advanced Healthcare Materials (2019).

Clarifying Geometry Effects in Figure 3
Figure 3 employs four different geometries to study curvature effects; however, variations in other geometric features (e.g., symmetry, surface connectivity) may confound interpretation. A more rigorous design—e.g., sequential curvature variation within a single base geometry—could provide clearer insights into curvature-induced endothelial responses.

Quantification of Endothelial Alignment
The manuscript reports improved endothelial alignment within gyroid scaffolds based on qualitative imaging. However, quantitative metrics such as alignment index or orientation histograms are not provided. Including such analyses would provide stronger support for curvature-induced alignment.

Clarifying Spheroid Interpretation in Figure 5a
The cellular clusters observed in gyroid scaffolds are described as spheroids, but fluorescence imaging alone does not distinguish between multicellular aggregates and enlarged single cells. Incorporating nuclear staining (e.g., DAPI) would improve interpretation and confirm spheroid formation.

Tumor Volume Data and Visual Evidence in Figure 6b
While tumor growth differences between scaffold types are quantitatively shown, representative images of explants would enhance interpretation. Including visual documentation alongside growth curves would provide more immediate context, as recommended in prior vascularization studies.
Suggested references: Gao et al., Advanced Functional Materials (2017); Shukla et al., International Journal of Bioprinting (2024).

Minor Comments

Figure Panel Labeling
In Figure 1, the panel labels (e.g., f–g) are not in sequential order, which may hinder reader comprehension. Consider reordering the panels or adjusting the labeling for improved clarity.

Comparative Design Summary
A brief comparison of the gyroid and lattice scaffold systems—including aspects like fabrication complexity, resolution, and biological outcomes—would clarify the rationale for selecting gyroid architecture. A summary table or concise comparison would aid reader understanding.

Marker Labels in Figure 5a
Adding staining marker names directly onto the fluorescence images in Figure 5a would facilitate easier interpretation.

Terminology Consistency
Terms such as "gyroid scaffold" and "gyroid structure" are used interchangeably across the manuscript. Consistent terminology throughout the text, figures, and captions is recommended.

Reviewer #3

(Remarks to the Author)
I co-reviewed this manuscript with one of the reviewers who provided the listed reports. This is part of the Nature Communications initiative to facilitate training in peer review and to provide appropriate recognition for Early Career Researchers who co-review manuscripts.

Reviewer #4

(Remarks to the Author)
Review manuscript NCOMMS-25-23043
"Translating 3D architectures of hydrogel triply periodic minimal surface (TPMS) scaffold to the functions of volumetric tissue constructs"
English needs to be edited: long sentences, not always with proper grammar.
Typos: Line 71: (aka cell printing), please avoid colloquialisms. Uniform the use of italics throughout the text (e.g. in vivo and in vitro).

Abstract
Recapitulate the study but could be more precise

Introduction
Introduction well structured that provides the required information, context and rationale but that is a little too long. It would be sharper and more straight to the point if it would be condensed a little.
It is a detail but I wouldn't mention experimental details like cell number in the introduction. The last paragraph seems more like an abstract with appreciative words than an introduction (for example, successfully, improved, etc.).
A comment about the possible structures in the organism that are indeed gyroids is advised to highlight the interest of changing from a lattice structure to the one proposed.
It is not clear why a cancerous cell line (HepG2) has been used in the context of the study (Tissue engineering) and why tumor formation is considered a success by the authors. I would rather use more relevant cell types (i.e. iPSC, hepatocyte progenitors, MSC) and focus on cell functionality as it is the key parameter for the proof-of-concept. If not, I would rather present the construct as a tumor model rather than an engineered tissue.
Results and Discussion
The authors have elected to have one results and discussion part. But sometimes, the discussion should provide more links and positioning with and toward the existing literature. It would help to assess the impact of the work.
Optimal design, Figure 1
- It is not clear how the different UC were selected, based on which rational and why the values are different between Fig1 and S1.
- When changing RD and UC, does this change the global amount of material in the scaffold or does it stay constant?
- What is the impact of RD and UC changes on the total porosity?
- If the scaffold is 1.5 mm high, it is not very thick. So, the diffusion of oxygen and nutriment is not as much a challenge as when the scaffold is bigger. The justification (ethical requirement) is not very clear, especially when it is not very complicated to implant bigger structures subcutaneously in mice.
- - I don't understand the sentence starting in line 174 to 177. What is the relevance of the C2C12 cells here? The information is confusing as the authors reference the figure 1c and then describe results from another paper (Ref. 41). It can be misleading as the reader might think that that these cells were used in this study.
- Fig1e: I guess that you don't really need modelling to anticipate that a scaffold with the same mass of polymer (it seems the case from S1 even if the information is not provided) but with smaller pores and thicker walls will support a lower diffusion of oxygen and then will have a lower cell viability. I don't think that result required the
- Why pore size does not increase oxygen diffusion and thus concentration in the scaffold (Fig1f)? I am not convinced by the authors. For a same wall thickness, bigger pores should increase diffusion.
- Fig1g and f: it is clear that the percentage of living cells decreased when the RD increased (quite logically), but what about the total number of living cells in the scaffold?
- The authors then state that since the cell viability is higher, RD20 was selected for further analysis but, one might consider it acceptable to lose some of the cells if the total number of living cells is higher, with RD50, especially since it is as low as 15%. But again, it will depend on whether the mass of polymer in the scaffold is the same or not and how many cells in total are incorporated in the scaffold. I would have balanced cell viability with mechanical properties to make that choice. It would then have been interesting to provide the elastic moduli for the different RD and UC.
- Fig1j: the authors state that the scaffold RD20-2.5 mm has the same modulus than the liver at 12kDa, which is 2 to 4 time higher than the liver. Is it really correct then? While the impact on the cell. Viability can be minimized, what would be the impact of implanting a stiffer scaffold in the liver or what would be the impact on hepatic cell function?
- Fig1k: what is the pore size of the lattice? It looks like over 1 mm when it is smaller for the gyroid. I don't think they could be compared then, especially as the wall thickness is different too. By increasing the number of pores, it should have been possible to get closer to the same wall thickness and pore size, shouldn't it?
Also, when I see the geometry of the lattice, I can not understand how oxygen diffusion can be reduced, especially it is the general concentration of oxygen that is measured. Wasn't it because there were more cells in the lattice? How many cells per scaffold were there?
Lines 232-234; avoid introducing ahead "future" results.

Vascularisation, Figures 2 &3
- I didn't understand why the authors stated that "to eliminate the impact of other supporting cells … ", lines 258-262. Wouldn't be more relevant to have pericytes also?

- Lines 276-277; the authors state that an active perfusing culture is difficult to translate to in vivo applications. Wouldn't in vivo have an active diffusion of nutrients in a physiological way? How can a static culture be more easily translated to the in vivo state?
- I don't understand the sentence from line 288-289. How the authors made this conclusion based only on Figure 2c, which is just a live and dead assay? Same with the following sentence (lines 289-292): first, there is no proof here that cell death is due to hypoxia and second, the images presented here do not support the conclusion as it is impossible to see a red signal. But I agree that in figure 2d then, the hypoxia seemed more pronounced. This part probably needs some editing in the way it is written.
- Again, I don't think Figure 2c supports the claim made lines 295-299.
- Figure 3: why in figure 3f the data are the result of n=30 samples and in Figures 3g and h, the data are the result of n=3 experiments? It would be better to homogenize the way the data are presented.
- Line 325: Is it enhanced vascularization or is it reduced in the lattice condition? Do the authors have a control sample of a typical angiogenesis assay such as collagen I or even GelMA to evaluate the HUVEC intrinsic potential of vascularization? As the culture conditions are not specific for HUVECs and the passage (which is key for maintaining their vascularization potential) is not disclosed it would be interesting to have a positive control.
Generation of vascularized liver tissue
- Figure 4c relates cell proliferation but the authors used a metabolic assay. So if no quantification of cell number (via DNA or protein quantification for instance) has been done, the authors should rather use "cell metabolic acivity" rather than "cell proliferation". Indeed, cells number could be the same with a higher metabolic activity in the gyroid compared to the lattice, or cell number could be lower in the lattice but with a higher activity than in the gyroid (as it could be the case, since viability is lower but the metabolic activity is still stable over time in the lattice). There is by the way no discussion making a link between data in figure 4b and 4c while both parameters are linked.
- Why in Figure 4d, Pimonidazole, is there no signal in the xy view while a signal is visible in the xz and yz views?
- Figure 4e: no "s" at the Hypoxic cells ratio.
- Figure 4h&i: before comparing the relative intensity of both cell types, did the authors check that the 2 cell types present the same intensity? Why they are no SD bars on the graph and no mention of the number of replicates.
- Figure 4d: same comment: why no SD bars and no info on replicates?
- It is not clear for me why, as the title of this part specifically relates to "vascularized liver tissue construct with promoted hepatic function", the authors seemed to relate the formation of tumor-like structures (and later on, of tumors in vivo) as a success or a desirable thing. I don't believe tumor-like structures are normal tissue constructs and are desirable in the scope of tissue engineering. I think the authors should reorient their paper toward a hepatic tumor model rather than normal hepatic tissue and tissue engineering.
- On which basis the authors claim that increased expression of ALB and MRP2 is a sign of enhanced tissue function and maturation and not a sign of tumorigenicity?
- I don't understand the link between "few studies (line 546)… and … due to its known advantages in bone tissue engineering (line 548)".

Enhanced tumor tissue formation and neovascularization in vivo
- Again, I don't see the link between tissue engineering, in vivo functionality, engineered tissue constructs, and tumor formation. Figure 6 legend states 'in vivo functions of the engineered tumor tissue constructs" but the data relates only to vascularization and not function.
- I don't think enhancing tumor growth is as difficult as producing a well-vascularized normal functional tissue, so the authors should tone down their statements, as it is a oversold as it is.
- Lines 612-615: here the authors mentioned tumor models, which indeed is more in line with the data. They should adapt the whole paper to this goal rather than tissue engineering.
- As a general comment on these results; cancer tumor models (such as matrigel based) allow the incorporation of cells from the animal (such as Tumor associated macrophages (TAM) or cancer associated fibroblasts (CAFs)) for the formation of the tumor. How is this 3D structure comparable? Is there formation of tissue in the pore areas of the material that were empty? Could the authors analyze the other cells forming the tumor besides the HepG2 and HUVECs to explain the differential in growth between conditions?
- Fig. S8 shows histological images of the tumors, scales are missing. What is the aspect of the holes of the 3D structure? From the images, the lattice sample seems like there is no tissue in the porous structure.
- Do the authors assess if the methacrylated gelatin was degraded in vivo? Do the authors expect the printed material to disappear at some point?
- Do the authors expect the encapsulated cells to "escape" the scaffold in vivo?
Materials and methods
- The resources like the mathematical model and the .slt files should be available for reproducibility.
- Line 698: Indicate origin, passage and cell culture conditions as for HUVECs response is important. Add information on how the cells were incorporated, dissolved in medium/PBS? how much volume? Also why you didn't use a specific medium for HUVECs (line 750)?
- Line 707: explain how sterility was ensured during the printing process.
- Line 727: Indicate number of gels per test and if they were from the same batch
- Line 736: Did the authors tested hydrogels from different fabrication batches?
- Cell culture: indicate cell passage used for both cell types.
- Cell membrane fluorescent staining: Add information about time of labelling and how the excess was removed
- Endothelial networks analysis: Add information in how many images per sample and areas quantified in total.
- Statistical analysis: number of samples, replicas and independent experiments is too ambiguous. It should be clearly stated in the manuscript. To use a t-test, a normality test of the results should be run. Also, the authors use t-test when more than two conditions are compared, therefore an ANOVA should be used, either one way or two way, when different times are also

included. Please redo all statistics considering all of this.

Reviewer #5

(Remarks to the Author)
I co-reviewed this manuscript with one of the reviewers who provided the listed reports. This is part of the Nature Communications initiative to facilitate training in peer review and to provide appropriate recognition for Early Career Researchers who co-review manuscripts.

Version 1:

Reviewer comments:

Reviewer #1

(Remarks to the Author)
No further comments.

Reviewer #2

(Remarks to the Author)
This manuscript, for which a major revision was recommended in April 2025, develops hydrogel-based triply periodic minimal surface (TPMS) gyroid scaffolds fabricated by DLP 3D printing of cell-laden GelMA, and extends them into HepG2–HUVEC co-cultured vascularised liver tumour constructs and subcutaneous implantation models in mice. The three structural concerns raised in the first round—(i) reproducibility limitations due to insufficient gyroid design information, (ii) insufficient control of confounding variables between lattice and gyroid, and between planar and hyperboloidal regions, and (iii) lack of quantification in endothelial alignment and multicellular aggregate interpretation—have been addressed satisfactorily in most respects.
Specifically, the level-set generation algorithm used in MSLattice and the main design parameters (unit cell size, relative density, and sheet-network selection) are now given in Supplementary Note 1, and eight STL files (Gyroid UC1.7/2.5/3.5 at RD20% and four lattice geometries) have been deposited on a Figshare repository, substantially resolving the reproducibility concern. The multi-step rationale for the RD–UC selection (printability → mechanical modulus → oxygen transport → cell response) has been reconstructed across Fig. 1 and Supplementary Fig. 3; within-family isolation of curvature effects is now provided by contrasting planar and hyperboloidal regions within a single gyroid geometry, with molecular-level mechanotransduction evidence for integrin β1 upregulation and YAP nuclear translocation (Fig. 3j–l and Supplementary Fig. 12). The endothelial alignment index has been quantified in Supplementary Fig. 8; the CD31/Albumin/DAPI triple-channel imaging in Fig. 5c now supports the multicellular nature of the HepG2 aggregates; and day-24 explant photographs of in vivo tumours are added to Fig. 6c. Minor items (Comment 9 panel ordering, Comment 10 comparison summary in Supplementary Table 2, Comment 11 HepG2 marker label in Fig. 5a, and Comment 12 terminology consistency) have also been appropriately resolved.
Two items, however, remain unresolved. First, the Fig. 5c caption still lists only "albumin (ALB) for HepG2 (green) and CD31 for HUVEC (red)" and does not reflect the DAPI channel that is present and labelled in the image itself—a caption–image mismatch that may cause readers to miss the visual evidence supporting the multicellular interpretation. Second, the four references recommended in the first round in support of Comment 3 (curvature-driven vasculature), Comment 4 (geometry- and curvature-based parameter matching for cell-fate guidance), and Comment 8 (in vivo vascularization and hepatic tissue engineering documentation) have not been cited or discussed in this revision. These include two of our own group's publications [1, 4] together with two closely related studies from other groups, namely Werner et al. 2017 Advanced Science [2] on parameter-matched curvature control of stem cell migration and differentiation, and Moon et al. 2025 Materials Today Bio [3] on gyroid-architecture vascularized hepatic constructs. Our group's only citation in both the original and the revised manuscript remains D. W. Cho, Biofabrication 8, 015007 (2016). A minor revision is therefore recommended with the comments below.

Minor comments
1. The Fig. 5c image clearly shows a CD31/Albumin/DAPI triple-channel overlay, with the DAPI channel visible in blue and labelled in the panel, which fully resolves the previous concern regarding the distinction between multicellular aggregates and enlarged single cells. However, the current Fig. 5c caption lists only "albumin (ALB) for HepG2 (green) and CD31 for HUVEC (red)" and does not mention the DAPI channel that is already present in the image. We would kindly ask the authors to update the Fig. 5c caption to include the DAPI channel and to note, in one short sentence, that the nuclear staining supports the multicellular nature of the aggregates. If a simple quantitative descriptor (for example the average number of nuclei per aggregate in representative regions) can be extracted from the existing confocal data, adding it would further solidify the spheroid interpretation; however, this is optional and need not require additional experiments. The Fig. 6f caption already lists CD31/AFP/DAPI explicitly and requires no change.
2. In the first round, four references [1–4] were recommended as closely related prior work on curvature-driven vasculature, curvature/geometry-based cell-fate guidance, and in vivo vascularisation and hepatic tissue engineering documentation. As these references were not cited or discussed in the revised manuscript, we would kindly ask the authors to revisit them in the Discussion with one or two sentences each, briefly comparing matrix, fabrication strategy, geometric control, and biological readout relative to the present study. Reference [1] (Park et al., Nature Communications 2023) is most directly relevant to Comment 3, as it demonstrates how vascular geometry controls endothelial behaviour and cancer-cell dynamics; reference

[2] (Werner et al., Advanced Science 2017) is highly relevant to Comment 4, as it isolates surface curvature as an independent geometric variable under parameter-matched conditions and links it to stem-cell migration and differentiation via attachment morphology and nuclear deformation; reference [3] (Moon et al., Materials Today Bio 2025) is closely aligned with the present work and with Comment 8, as it reports gyroid-architecture thick vascularised hepatic constructs; and reference [4] (Shukla et al., International Journal of Bioprinting 2024) provides a broader vascularisation-strategy context for Comment 8. Including these together would place the gyroid-based liver tumour platform more clearly within the existing soft-tissue curvature and vascularisation literature and provide appropriate context for the biological claims made in the manuscript.

Reviewer #3

(Remarks to the Author)
I co-reviewed this manuscript with one of the reviewers who provided the listed reports. This is part of the Nature Communications initiative to facilitate training in peer review and to provide appropriate recognition for Early Career Researchers who co-review manuscripts.

Reviewer #4

(Remarks to the Author)
Thank you for taking into account the remarks and recommendations, and for addressing them. I have no further request.

Reviewer #5

(Remarks to the Author)
I co-reviewed this manuscript with one of the reviewers who provided the listed reports. This is part of the Nature Communications initiative to facilitate training in peer review and to provide appropriate recognition for Early Career Researchers who co-review manuscripts.

Open Access This Peer Review File is licensed under a Creative Commons Attribution 4.0 International License, which permits use, sharing, adaptation, distribution and reproduction in any medium or format, as long as you give appropriate credit to the original author(s) and the source, provide a link to the Creative Commons license, and indicate if changes were made.
In cases where reviewers are anonymous, credit should be given to 'Anonymous Referee' and the source.
The images or other third party material in this Peer Review File are included in the article's Creative Commons license, unless indicated otherwise in a credit line to the material. If material is not included in the article's Creative Commons license and your intended use is not permitted by statutory regulation or exceeds the permitted use, you will need to obtain permission directly from the copyright holder.
To view a copy of this license, visit https://creativecommons.org/licenses/by/4.0/

Responses to Reviewers' Comments

Ref. No.: NCOMMS-25-23043

Title: Translating 3D architectures of hydrogel triply periodic minimal surface (TPMS) scaffold to the functions of volumetric tissue constructs

Authors: Xiaoxiao Han, Ning He, Dandan Yuan, Na Li, Wei Zhu, Xiaolong Zhu, Jing Li, Xun Yuan, Wenxin Wang, Xingshi Gu, Feng Chen, Wei Wang

Journal: Nature Communications

The authors are very grateful to the reviewers for their valuable comments. All the comments have been considered and addressed very carefully. The changes, where necessary, have also been incorporated into the revised manuscript/supplementary document and highlighted in yellow. Hopefully, the corrections would meet editor's and reviewers' requirements.

The comments and corresponding responses (highlighted in blue in this document) are listed point by point below.

<u>Comments from reviewer #1 and replies:</u>

We appreciate your valuable comments, which have significantly improved the quality of this manuscript. Our detailed responses are provided below.

C1: Although TPMS scaffolds have been explored for hard tissues (e.g., bone), their application in soft tissues remains under explored. The authors appropriately cite prior work but should clarify the differences from recent studies. (e.g.,10.1016/j.mtbio.2023.100667; 10.1073/pnas.2206684119).

Response: We have clarified the difference of TPMS scaffold between hard and soft tissue applications, in terms of matrix materials, fabrication strategy and cell-matrix interactions.

The following has been added to the Discussion section of the revised manuscript:

"Although gyroid scaffolding systems have shown promise in bone tissue engineering (38,55), their translation to soft tissue models is nontrivial due to fundamental differences in the ECM microenvironment, cellular properties, and cell-matrix interactions. Gyroid scaffolds designed for bone regeneration are typically fabricated from stiff materials such as wollastonite bioceramics or β-tricalcium phosphate, which exhibit elastic moduli in the MPa range and provide load-bearing support suitable for osteogenesis (36). In contrast, soft tissues require a compliant 3D matrix environment. To mimic this setting, we employed 10 wt% GelMA hydrogel, achieving an elastic modulus of approximately ~13.1 kPa, comparable to that of native liver tumour tissue. As matrix stiffness is a key mechanical cue regulating cell behaviour, such differences in material properties are expected to result in distinct biological responses (33). In addition to mechanical properties, cell incorporation strategies differ between bone and soft tissue scaffolds. In most bone tissue engineering studies (37,38,55), cells are seeded onto the scaffold surface after fabrication, resulting in relatively low cell densities and limited infiltration into the scaffold interior. In contrast, soft tissues are characterised by a dense and homogeneous 3D cellular distribution. To better recapitulate this physiological architecture, we encapsulated cells directly within the scaffold, generating a bulk, cellularised construct with a high cell density (2×10^{7} cells mL^{-1}). A major challenge in this context is that soft, cell-laden bioinks often suffer from poor printability due to their dilute polymer

content and cell-induced light scattering, particularly in complex 3D architectures with fine features (5). By leveraging the light-scattering inhibition strategy established in our previous work (32), we successfully fabricated cell-laden gyroid scaffolds with high structural fidelity. Furthermore, cell-matrix interactions differ between cell-seeded and cell-laden scaffolds. In cell-seeded curved scaffolds, cells mainly adhere to the scaffold surface and sense matrix properties through integrin-mediated adhesion and actomyosin-generated traction forces, which promote focal adhesion maturation and downstream signalling. Consistent with this, Yang et al. showed that the hyperboloidal curvature of gyroid scaffolds enhanced focal adhesion organisation and stress fibre formation in surface-seeded human mesenchymal stem cells (38). In contrast, in our cell-laden 3D system, cells were confined within a curved matrix, where mechanosensing could be governed by integrin binding and clustering, spatial confinement, and dynamic cell-matrix remodelling (33). Under these conditions, cells exhibited distinct cytoskeletal organisation, with more pronounced actin assembly and filopodia/lamellipodia formation. For mechanosensing behaviour, it has been demonstrated that increasing the curvature of gyroid scaffolds could promote osteogenic differentiation of human mesenchymal stem cells via focal adhesion kinase (FAK) activation (38). Compared with lattice scaffolds composed of planar struts, we observed that the cell-laden gyroid scaffold upregulated integrin β1 expression in ECs, which was accompanied by enhanced morphogenesis and network formation.”

C2: The conclusion that 3D curvature promotes vascular self-assembly lacks molecular evidence (e.g., YAP/TAZ signaling). Incorporate Western blot (WB) or qPCR data to analyze curvature-sensitive genes (e.g., YAP/TAZ-related proteins) and clarify whether curvature regulates endothelial cell behavior through specific pathways.

Response: Thank you for raising this important concern. In this study, the major differences in endothelial cell behaviour between the lattice and gyroid scaffolds were observed in actin cytoskeletal organisation and endothelial network formation. To explore the potential mechanism underlying these differences, we measured several mechanotransduction-related proteins (see figure below), including integrin β1, which is associated with actin cytoskeletal organisation, as well as YAP and phosphorylated YAP (p-YAP), which are involved in regulating endothelial cell migration, proliferation, vascular remodelling, and vessel formation. The 3D curvature of the gyroid scaffold was associated with upregulation of integrin β1, which is essential for organising actin filaments into protrusive structures such as filopodia. In addition, nuclear YAP levels were higher and YAP phosphorylation was reduced in the gyroid scaffold, suggesting enhanced YAP nuclear translocation.

The data has been added to the Results section and the following content has been added to the Discussion of the revised manuscript:

"*Geometric curvatures present in ECM have only recently been recognised as a physical cue regulating cellular behaviours, with most studies focusing on cells cultured in 2D systems, such as on top of curved substrates (33,34). However, in vivo, the ECM typically constrains cells within a 3D microenvironment, and the effects of 3D curvature on EC behaviour remain poorly understood. In natural vasculature, ECs, which form the inner lining of blood vessels, are exposed to curvature cues of various geometry (e.g. cylindrical curvature of vessels and hyperbolic curvature at vascular bifurcation), and extents ranging from 0.1 to 200 mm^{-1} (35). In this study, we demonstrated that a 3D curved volumetric architecture was an essential geometric cue capable of directing endothelial behaviour and function via mechanotransduction. Integrins, serving as physical signal receptors, have been identified as central regulators of mechanotransduction in 3D, mediating cytoskeletal organisation and the formation of protrusive actin structures such*

as filopodia. We found that the 3D curved gyroid architecture, with an moderate mean Gaussian curvature of -2.30 mm^{-2}, could upregulate integrin β1. Elevated integrin dynamics enable cells to better sense and respond to the physical properties of the ECM, which can lead to activation of various downstream pathways and transcription regulators (33). Among these, the YAP signalling has been shown to play an important role in regulating endothelial cell migration, proliferation, vascular remodelling and vessel formation (60). Li et al. (55) reported that gyroid scaffolds could suppress YAP/TAZ phosphorylation and enhance YAP/TAZ nuclear translocation of HUVEC seeded on scaffold surface, resulting in enhanced proliferation, migration, tube formation, and high expression of CD31. Consistent with this, in our soft cell-laden gyroid scaffold, we observed a significant increase in nuclear YAP levels accompanied by reduced YAP phosphorylation, indicating enhanced YAP nuclear translocation. It is noteworthy that the promotive effect on network formation was weakened significantly at a higher curvature (e.g. -5.00 mm^{-2}), likely because of increased local geometric confinement. Curvature-dependent changes in focal adhesion organisation, actin stress fibers, and traction force have been reported across curved and confined microenvironments, suggesting that excessive curvature may limit the spreading and force transmission needed for efficient cell migration and network formation (61,62). Collectively, these results identify 3D curvature as a critical biophysical cue regulating endothelial behaviour and highlight its potential to be harnessed in the rational design of bulk tissue-engineered constructs.”

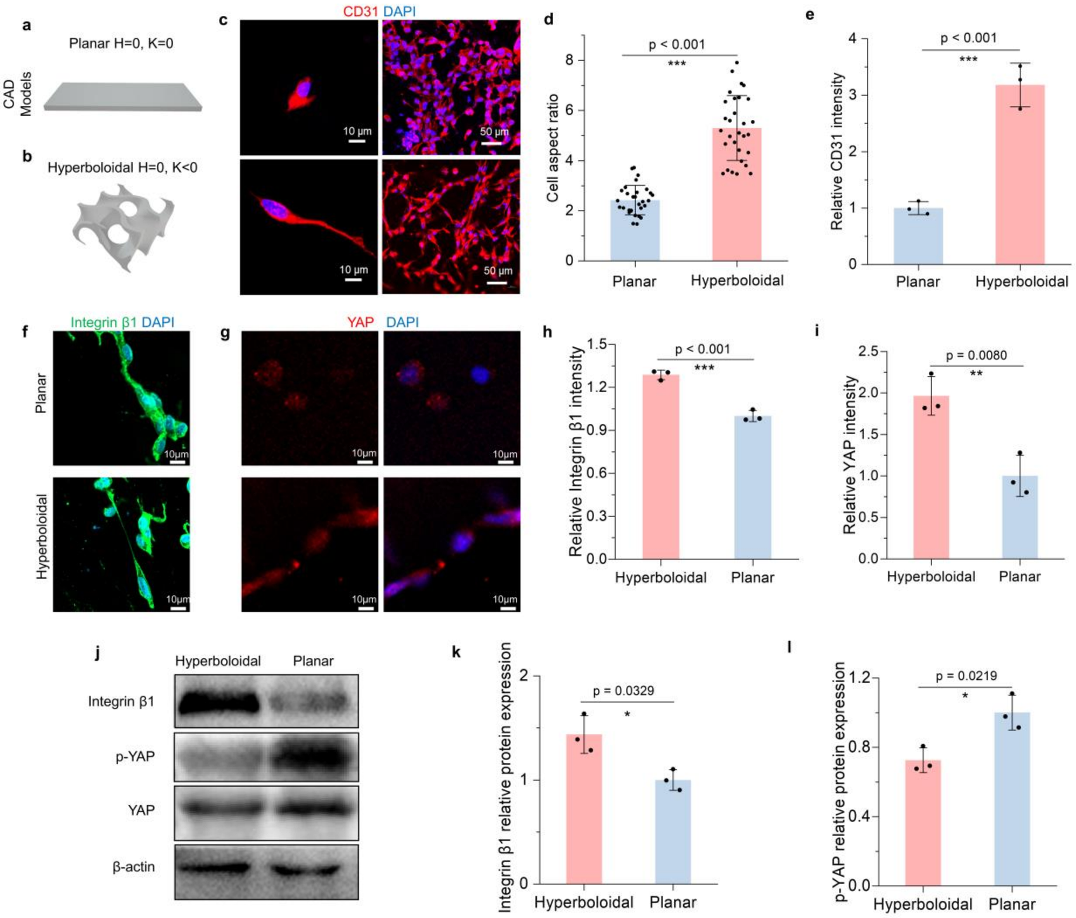

Note that the data has been added to Fig. 3.

C3: While Cur-Na improves printing precision, its long-term biocompatibility requires comprehensive validation.

Response: Cur-Na is derived from curcumin, a natural phenolic antioxidant widely used for antioxidant, anti-inflammatory, antimicrobial, and so on. In our previous study (1), we demonstrated its biocompatibility for cell proliferation and differentiation. The PEG-GelMA/Cur-Na hydrogel exhibited the uppermost cell viability, and the cell proliferation was approximately tripled after 14 days in culture, even higher than that of the pure PEG-GelMA bioink.

In this study, both HUVECs and HepG2 cells maintained high viability, robust proliferation, and preserved cellular functions, such as endothelial network formation and liver-specific activities, after 14 days of *in vitro* culture. The resulting pre-vascularised *in vitro* model was subsequently implanted subcutaneously into male severely immunodeficient mice. Following 24-day *in vivo* cultivation, no fibrotic encapsulation was observed around the scaffold, nor was there any evidence of non-specific inflammatory responses in the surrounding tissues. The mice organs, including heart, lung, kidney, spleen and liver, were under healthy conditions after the *in vivo* study of scaffold transplantation (Supplementary Fig. 18). These results demonstrate the biocompatibility of Cur-Na suitable for long-term applications.

The following has been added to the Results section in the revised manuscript:

"*Furthermore, after 24 days of in vivo cultivation, no fibrotic encapsulation was observed around the scaffold, nor was there any evidence of non-specific inflammatory responses in the surrounding tissues. In addition, major organs, including the heart, lung, kidney, spleen, and liver, exhibited normal histological features (Supplementary Fig. 18), demonstrating the good biocompatibility of the scaffolding system.*"

References:

1. N. He, X. N. Wang, L. Y. Shi, J. Li, L. Mo, F. Chen, Y. T. Huang, H. R. Liu, X. L. Zhu, W. Zhu, Y. Q. Mao, X. X. Han, Photoinhibiting via simultaneous photoabsorption and free-radical reaction for high-fidelity light-based bioprinting. Nature Communications 14, 3063 (2023).

C4: The scaffold's modulus matches hepatic tissue (~12 kPa) under static conditions but neglects physiological dynamic loads (e.g., organ pulsation, shear stress).

Response: GelMA is widely used in 3D bioprinting of soft tissue models owing to its excellent biocompatibility and inherent cell-adhesive properties. However, pure GelMA hydrogels generally exhibit limited mechanical strength due to their dilute polymer networks and are therefore primarily applied in non-load-bearing tissues (1), including the present study.

To evaluate the mechanical behaviour of the soft gyroid scaffolds under dynamic loading, we examined the stress-strain response under cyclic compression (see the figure below). At a compressive strain of 20%, the scaffold exhibited excellent elastic recovery, attributable to the combined effects of the intrinsic elasticity of the hydrogel matrix and the geometrical resilience of the gyroid architecture, which together provide effective resistance to dynamic loading (2). Consequently, the compressive modulus remained largely stable over successive loading cycles. A slight increase in modulus was observed during repeated compression, which can be attributed to the gradual expulsion of water from the scaffold. Such dehydration increases the polymer volume fraction and network density, leading to scaffold stiffening. In contrast, when the compressive strain was increased to 50%, the scaffold became mechanical unstable quickly, and collapsed after a few loading cycle.

20% compressive strain

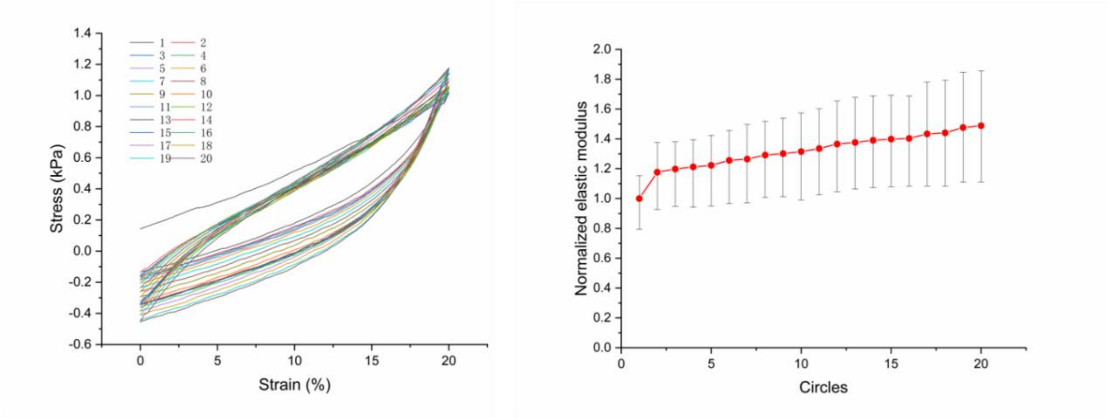

50% compressive strain

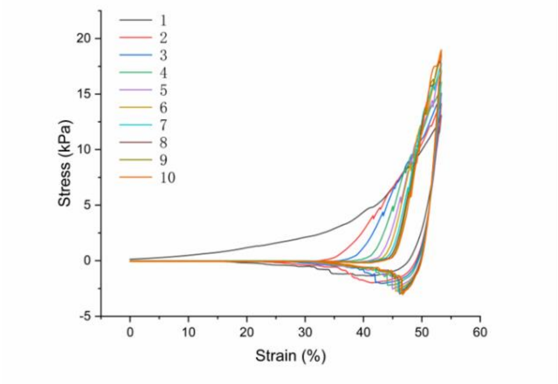

The following has been added to the Discussion section in the revised manuscript:

"we selected 10 wt% GelMA that supported high cell viability and provided a mechanically relevant matrix for liver cancer cell culture. Liver tumour stiffness is generally higher than that of normal liver (1–7 kPa) and varies with disease stage, increasing from fibrosis to cirrhosis and ultimately hepatocellular carcinoma, with representative ranges of 4–13 kPa, 9–25 kPa, and 14–72 kPa, respectively (41). The material stiffness of our 10 wt% GelMA was approximately 13.1 kPa, which lies at the lower end of the tumour-associated stiffness ranges. We then optimised the scaffold parameters to achieve adequate mass transport and mechanical stability, and appropriate curvature cues. This strategy produced a thin-walled gyroid architecture (~170 – 210 μm) with a moderate curvature that supported the formation of a dense and uniformly distributed vascular network throughout the entire 3D volume. This is important because one of the major challenges in engineering large tissue constructs is the development of hypoxic microenvironments in the scaffold core, which can lead to cellular dysfunction and necrosis (7). In contrast, the

lattice scaffold with an equivalent wall thickness exhibited severe structural instability because of the limited intrinsic mechanical strength of 10 wt% GelMA. The gyroid architecture could compensate for this weakness through its structural resilience, providing sufficient mechanical stability for both in vitro and in vivo studies. Nevertheless, the construct remained subject to relatively low compressive load tolerance and was therefore unsuitable for load-bearing applications such as bone and cartilage. For such applications, GelMA could be combined with reinforcing materials (e.g., alginate/methylcellulose or PEGDA) to enhance the mechanical performance of printed constructs (57,58).”

References:

1. M. Sun, X. Sun, Z. Wang, S. Guo, G. Yu, H. Yang. Synthesis and Properties of Gelatin Methacryloyl (GelMA) Hydrogels and Their Recent Applications in Load-Bearing Tissue. Polymers (Basel) 10(11), 1290 (2018).

2. X. Zhu, F. Chen, H. Cao, L. Li, N. He, X. Han. Design and fused deposition modeling of triply periodic minimal surface scaffolds with channels and hydrogel for breast reconstruction. Int J Bioprint. 9(2):685 (2023).

C5: Current evaluation focuses on structural metrics (CD31 expression, network length). Supplement with functional validation:

1) Expression of tight junction proteins (e.g., VE-cadherin, ZO-1) to assess vascular maturity.

Response: VE-cadherin is a crucial component of adherens junctions, where it mediates strong cell-cell adhesion through homophilic interactions. We have measured the difference in the expression level of VE-cadherin between lattice (planar) and gyroid (hyperboloidal curvature) geometries (see the figure below). The VE-cadherin expression of the gyroid was approximately 3.0-fold higher than that of flat geometry, indicating higher maturity.

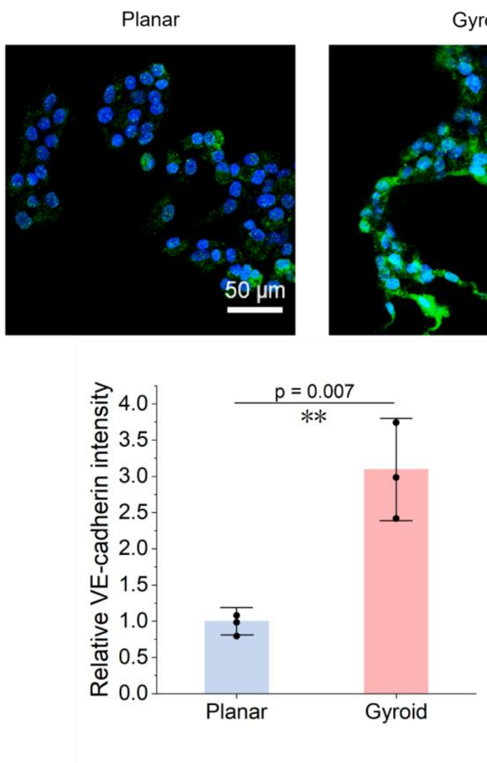

Note that the data has been included in the Supplementary Information.

2) Barrier functionality in co-culture models.

For barrier functionality, the vessels generated *in vitro* were not fully mature in this study because endothelial cells (ECs) were cultured without stromal/perivascular support cells (e.g., fibroblasts, MSCs, pericyte-like cells), which are known to promote vessel stabilisation and maturation. Pre-vascularisation has been widely reported as an effective strategy to enhance *in vivo* graft performance, including faster host-graft anastomosis, improved

survival of parenchymal cells, and increased vessel density and functional integration (1-4).

Several approaches have been used to promote pre-vascularisation prior to implantation, including incorporation of angiogenic growth factors, co-culture of ECs with stromal/perivascular support cells, immobilisation of bioactive molecules, and scaffold-based strategies (5). The aim of the present study was to demonstrate that the 3D architecture of the gyroid scaffold can facilitate rapid vascularisation and improve the viability and function of co-cultured parenchymal cells *in vivo* by supporting the formation of preliminary vascular structures *in vitro*. We intentionally used ECs alone *in vitro* to eliminate confounding contributions from supportive cells, particularly their effects on vessel stability and maturation, so that any differences in *in vitro* and *in vivo* outcomes could be attributed primarily to scaffold geometry.

Following implantation, maturation of EC-lined structures still occurs through recruitment of host support cells, provided that the construct offers a permissive microenvironment for host-graft integration (6). We demonstrated that the gyroid scaffold promoted capillary network formation *in vitro* by supporting adequate oxygen diffusion and providing a curvature-induced mechanical cue. Translation of the gyroid scaffold to the *in vivo* setting significantly enhanced both tumour tissue formation and vascularisation (Fig. 6). We did not observe edema or evidence of extracellular leakage during the *in vivo* study, and the capillary network architecture remained intact.

The following discussion has been added to the revised manuscript:

"*Several approaches have been used to enhance pre-vascularisation before implantation, including the incorporation of angiogenic growth factors, co-culture of ECs with stromal or perivascular support cells, immobilisation of bioactive molecules, and scaffold-based strategies (63). In the present study, our aim was to demonstrate that the 3D architecture of the gyroid scaffold can accelerate vascularisation and improve the viability and function of co-cultured parenchymal cells in vivo by supporting the formation of preliminary vascular structures in vitro. We used ECs alone to eliminate confounding contributions from support cells, particularly their effects on vessel stability and maturation, so that differences in in vitro and in vivo outcomes could be attributed more directly to scaffold geometry. Following implantation, maturation of EC-lined*

structures can still occur through recruitment of host support cells, provided that the construct offers a permissive microenvironment for host-graft integration (3).”

References:

1. Mazio C, Casale C, Imparato G, Urciuolo F, Attanasio C, De Gregorio M, Rescigno F and Netti P A, Pre-vascularized dermis model for fast and functional anastomosis with host vasculature. Biomaterials. 2019, 192:159-70.

2. Bang S, Tahk D, Choi Y H, Lee S, Lim J, Lee S R, Kim B S, Kim H N, Hwang N S and Jeon N L, 3D microphysiological system-inspired scalable vascularized tissue constructs for regenerative medicine. Adv. Funct. Mater. 2022, 32:2105475.

3. Song W, Chiu A, Wang LH, Schwartz RE, Li B, Bouklas N, Bowers DT, An D, Cheong SH and Flanders JA, Engineering transferrable microvascular meshes for subcutaneous islet transplantation. Nat. Commun. 2019, 10 1-12.

4. Liew AWL, Zhang AY. In vitro pre-vascularization strategies for tissue engineered constructs-Bioprinting and others. Int J Bioprint. 2017, 3(1):008.

5. Soraia V. Lopes, Maurice N. Collins, Rui L. Reis, Joaquim M. Oliveira, and Joana Silva-Correia, Vascularization Approaches in Tissue Engineering: Recent Developments on Evaluation Tests and Modulation. ACS Applied Bio Materials, 2021, 4 (4):2941-2956.

6. Gimble JM, Marolt D, Oreffo ROC, Wolbank S, Redl H, (Eds.), Reference Series in Biomedical Engineering-Tissue Engineering and Regeneration. Cell Engineering and Regeneration. Springer Cham, 2020.

C6: Define abbreviations upon first mention (e.g., *RD: radial distance; UC: umbilical cord*).

Response: We appreciate the reviewer’s suggestion. All abbreviations have been carefully reviewed and their definitions have been corrected or clarified where necessary throughout the manuscript.

C7: Correct grammatical errors (e.g., tense consistency, article usage).

Response: We have gone through the entire paper carefully, and corrected the errors, including grammar, typo, tense consistency, and etc.

Comments from reviewer #2 and replies:

We appreciate the reviewer's valuable comments, which have significantly improved the quality of this manuscript. Our detailed responses are provided below.

C1: Gyroid Design Details: The gyroid architecture is a central feature of the study, yet the manuscript lacks essential information on its generation. Details such as the mathematical definition, generation algorithm, and design parameters (e.g., periodicity, amplitude, spacing) are not provided. This omission limits reproducibility. Including such information in the Supplementary Materials—along with representative STL files—would significantly improve the manuscript's utility and translational value.

Response: We have provided the detailed information for generating the gyroid scaffold, including mathematical definition, generation algorithm, and design parameters (Supplementary Note 1). The STL files used in this study will be uploaded to an open-access repository during re-submission.

The following has been added to Supplementary Note 1:

"The gyroid scaffold geometry was created using the MSLattice software (1) developed for generating uniform and functionally graded lattice structures based on triply periodic minimal surfaces (TPMS). The software employs a level-set approximation approach to describe the gyroid minimal surface that is defined mathematically using the following trigonometric equation:

$$\phi(x, y, z) = cos(\omega x)sin(\omega y) + cos(\omega y)sin(\omega z) + cos(\omega z)sin(\omega x) = c \qquad (1)$$

where the function $\phi(x, y, z)$ is an iso-surface evaluated at the level offset value c and $c = 0$ stands for the zero level offset of the function (the true minimal surface); $\omega = \frac{2\pi}{L}$ and L is the length of the unit cell. The software uses implicit surface modelling to represent, manipulate, and propagate unit cells within a given volume. By offsetting the minimal surface along and against its normal direction to create a double surface, the volume is generated by solving $-c \leq \phi(x, y, z) \leq c$. By providing the following input parameters: relative density (0-100%), size of the unit cell, sample length, sample width, sample height, and mesh density points, the software can generate both sheet (surface) and solid networks. In this study, all gyroid structures were created using sheet networks."

C2: Design Rationale for UC = 2.5 mm: The optimization of relative density (RD) and unit cell (UC) size is well supported by simulations and mechanical testing. However, the rationale for selecting UC = 2.5 mm remains unclear, especially given that Figure 1j suggests UC = 3.0 mm more closely aligns with physiological liver stiffness. Further clarification of this design choice beyond compressive strength would help justify the scaffold parameters in the context of hepatic applications.

Response: We have reshaped the interpretation logic for clarifying the design process of the gyroid scaffold (Fig. 1).

We first determined the concentration of the GelMA material. A 10 wt% GelMA formulation is widely used for engineering 3D tissue constructs because it provides a practical balance between structural integrity and biological compatibility (1).

Regarding matrix stiffness, in the originally submitted manuscript we conflated normal liver tissue and liver tumour tissue, treating “liver tumour” as representative of liver tissue in general. Because our model uses HepG2 hepatocellular carcinoma cells, we now explicitly refer to the modulus of liver tumour tissue when discussing physiological relevance, and we have clarified this distinction in the revised manuscript. Matrix stiffness is an important biomechanical cue in the tumour microenvironment. Increased stiffness can provide greater physical support and has been associated with altered cell adhesion, cytoskeletal organisation, and invasive/migratory behaviour (2). Reported moduli for liver tumours are generally higher than those of normal liver (1-7 kPa), and span a broad range depending on disease stage. During progression from fibrosis to cirrhosis and ultimately hepatocellular carcinoma, stiffness can increase substantially; representative reported ranges include approximately 4-13 kPa for fibrosis, 9-25 kPa for cirrhosis, and 14-72 kPa for hepatocellular carcinoma (2). In our study, the measured stiffness of 10 wt% GelMA was ~13.1 kPa (Supplementary Fig. 2), which falls within the lower end of tumour-associated stiffness ranges and provides a mechanically relevant matrix for liver cancer cell culture. Note that Fig. 1j presents the compressive structural modulus, which was used to characterise the structural stability of the scaffold architecture. In contrast, the material modulus is generally measured using standard samples (e.g., cylindrical specimens) and is typically used to

describe the intrinsic matrix stiffness of the biomaterial.

We then focused on the material exchange capacity because it is key to cell viability. We found that relative density (RD) was the dominant design parameter affecting diffusion, while the impact of unit cell (UC) was minor. RD was inversely proportional to matter diffusion, and smaller RD values (e.g. RD=10% or 20%) promoted diffusion significantly by offering higher surface specific area, thinner wall thickness and larger pore size.

We next added the constrain of structural stability. For soft hydrogel 3D printing, the conflict between structural stability and cell viability always exists. Theoretically, RD=10% offers the best material exchange performance and thus the highest cell viability. However, it gives rise to a gyroid with a extremely thin-walled structure with a wall thickness less than 90 μm. Significant deformation and distortion were observed after printing. So, RD=20% was adopted because it ensured adequate cell viability while maintaining structural stability.

We considered the mechanical property of the overall structure that impacts the *in vivo* structural stability. When RD (20%) was fixed, by varying UC from 2.0 to 3.5 mm, the compressional modulus were approximately 10.8, 12.0, 8.7 and 9.9 kPa, respectively. UC = 2.5 mm offered the highest modulus, providing better supporting capability.

Although UC showed minimum impact on oxygen diffusion, it could alter the curvature of the scaffold, which might deliver different geometric stimulation to cells. We finally investigated the impact of UC on capillary network formation. Varying UC size (1.7, 2.5 and 3.5 mm) produced different mean Gaussian curvatures (-5.00, -2.30 and -1.25 mm^{-2}), corresponding to high, moderate and low curvature magnitudes, respectively. Compared with the scaffold with low and high curvature magnitudes, cells cultured in the scaffold with moderate curvature (-2.30 mm^{-2} with UC = 2.5 mm) exhibited a more elongated morphology and significantly improved capillary network parameters (Supplementary Fig. 3). To this, the geometric parameters (RD = 20% and UC = 2.5 mm) not only provided adequate diffusion capacity and mechanical stability, but also delivered stronger geometric stimulation for capillary formation. Therefore, this optimised architecture was used in the subsequent studies.

References:

1. Mendoza-Cerezo L, Rodríguez-Rego JM, Macías-García A, Callejas-Marín A, Sánchez-Guardado L, Marcos-Romero AC. Three-Dimensional Bioprinting of GelMA Hydrogels with Culture Medium: Balancing Printability, Rheology and Cell Viability for Tissue Regeneration. Polymers (Basel). 2024, 16(10):1437

2. Min K, Karuppannan SK, Tae G. The impact of matrix stiffness on hepatic cell function, liver fibrosis, and hepatocellular carcinoma-Based on quantitative data. Biophys Rev (Melville). 2024, 5(2):021306.

C3: Comparison with Prior Curvature-Based Models: The manuscript would benefit from a direct comparison with existing curvature-driven vascular models. Recent studies have demonstrated that curvature influences endothelial behavior and cancer cell dynamics. A critical comparison would contextualize the gyroid scaffold's novelty and relevance. Suggested reference: Park et al., Nature Communications (2023).

Response: Thanks for raising this important question. We have compared this study with previous curvature-based models, with particular emphasis on gyroid-related systems. A corresponding discussion has been added to the revised manuscript, addressing differences and similarities in matrix materials, fabrication strategies, cell-matrix interactions, and the underlying molecular mechanisms. Note that the molecular evidence for 3D curvature-induced vascular self-assembly promotion has been added (see the figure below).

The following has been added to the Discussion section in the revised manuscript:

"*Although gyroid scaffolding systems have shown promise in bone tissue engineering (38,55), their translation to soft tissue models is nontrivial due to fundamental differences in the ECM microenvironment, cellular properties, and cell-matrix interactions. Gyroid scaffolds designed for bone regeneration are typically fabricated from stiff materials such as wollastonite bioceramics or β-tricalcium phosphate, which exhibit elastic moduli in the MPa range and provide load-bearing support suitable for osteogenesis (36). In contrast, soft tissues require a compliant 3D matrix environment. To mimic this setting, we employed 10 wt% GelMA hydrogel, achieving an elastic modulus of approximately ~13.1 kPa, comparable to that of native liver tumour tissue. As matrix stiffness is a key mechanical cue regulating cell behaviour, such differences in material properties are expected to result in distinct biological responses (33). In addition to mechanical properties, cell incorporation strategies differ between bone and soft tissue scaffolds. In most bone tissue engineering studies (37,38,55), cells are seeded onto the scaffold surface after fabrication, resulting in relatively low cell densities and limited infiltration into the scaffold interior. In contrast, soft tissues are characterised by a dense and homogeneous 3D cellular distribution. To better recapitulate this physiological architecture, we encapsulated cells directly within the scaffold, generating a bulk, cellularised construct with a high*

cell density (2×10^7 cells mL^{-1}). A major challenge in this context is that soft, cell-laden bioinks often suffer from poor printability due to their dilute polymer content and cell-induced light scattering, particularly in complex 3D architectures with fine features (5). By leveraging the light-scattering inhibition strategy established in our previous work (32), we successfully fabricated cell-laden gyroid scaffolds with high structural fidelity. Furthermore, cell-matrix interactions differ between cell-seeded and cell-laden scaffolds. In cell-seeded curved scaffolds, cells mainly adhere to the scaffold surface and sense matrix properties through integrin-mediated adhesion and actomyosin-generated traction forces, which promote focal adhesion maturation and downstream signalling. Consistent with this, Yang et al. showed that the hyperboloidal curvature of gyroid scaffolds enhanced focal adhesion organisation and stress fibre formation in surface-seeded human mesenchymal stem cells (38). In contrast, in our cell-laden system, cells were confined within a 3D curved matrix, where mechanosensing could be governed by integrin binding and clustering, spatial confinement, and dynamic cell-matrix remodelling (33). Under these conditions, cells exhibited distinct cytoskeletal organisation, with more pronounced actin assembly and filopodia/lamellipodia formation. For mechanosensing behaviour, it has been demonstrated that increasing the curvature of gyroid scaffolds could promote osteogenic differentiation of human mesenchymal stem cells via focal adhesion kinase (FAK) activation (38). Compared with lattice scaffolds composed of planar struts, we observed that the cell-laden gyroid scaffold upregulated integrin β1 expression in ECs, which was accompanied by enhanced morphogenesis and network formation."

"*Geometric curvatures present in ECM have only recently been recognised as a physical cue regulating cellular behaviours, with most studies focusing on cells cultured in 2D systems, such as on top of curved substrates (33,34). However, in vivo, the ECM typically constrains cells within a 3D microenvironment, and the effects of 3D curvature on EC behaviour remain poorly understood. In natural vasculature, ECs, which form the inner lining of blood vessels, are exposed to curvature cues of various geometry (e.g. cylindrical curvature of vessels and hyperbolic curvature at vascular bifurcation), and extents ranging from 0.1 to 200 mm^{-1} (35). In this study, we demonstrated that a 3D curved volumetric architecture was an essential geometric cue capable of directing endothelial behaviour and function via*

mechanotransduction. Integrins, serving as physical signal receptors, have been identified as central regulators of mechanotransduction in 3D, mediating cytoskeletal organisation and the formation of protrusive actin structures such as filopodia. We found that the 3D curved architecture in gyroid, with an moderate mean Gaussian curvature of -2.30 mm^{-2}, could upregulate integrin β1. Elevated integrin dynamics enable cells to better sense and respond to the physical properties of the ECM, which can lead to activation of various downstream pathways and transcription regulators (33). Among these, the YAP signalling has been shown to play an important role in regulating endothelial cell migration, proliferation, vascular remodelling and vessel formation (60). Li et al. (55) reported that gyroid scaffolds could suppress YAP/TAZ phosphorylation and enhance YAP/TAZ nuclear translocation of HUVEC seeded on scaffold surface, resulting in enhanced proliferation, migration, tube formation, and high expression of CD31. Consistent with this, in our soft cell-laden gyroid scaffold, we observed a significant increase in nuclear YAP levels accompanied by reduced YAP phosphorylation, indicating enhanced YAP nuclear translocation. It is noteworthy that the promotive effect on network formation was weakened significantly at a higher curvature (e.g. -5.00 mm^{-2}), likely because of increased local geometric confinement. Curvature-dependent changes in focal adhesion organisation, actin stress fibers, and traction force have been reported across curved and confined microenvironments, suggesting that excessive curvature may limit the spreading and force transmission needed for efficient cell migration and network formation (61,62). Collectively, these results identify 3D curvature as a critical biophysical cue regulating endothelial behaviour and highlight its potential to be harnessed in the rational design of bulk tissue-engineered constructs."

C4: Geometric Parameter Matching in Scaffold Comparison: When comparing gyroid and lattice scaffolds to assess curvature-specific effects, it is crucial to control for confounding factors such as volume fraction and wall thickness. Without such controls, observed cellular differences may arise from structural heterogeneity rather than curvature per se. Ensuring matched mechanical and geometric parameters would enhance the validity of biological comparisons. Suggested reference: Kim et al., Advanced Healthcare Materials (2019).

Response: Unlike hard tissue scaffolds, the design space and manufacturability of hydrogel maaterials are significantly constrained due to their soften and highly dilute nature. In 3D bioprinting, printability, cell viability, and structural stability are the three core pillars of the "biofabrication window" (1). Achieving success requires a delicate balance, as optimising one often compromises another.

In this study, the volume fraction, cell parameters (density and total number) and bulk mechanical properties of the lattice scaffold were adjusted to match with that of gyroid scaffold. The wall thickness for the gyroid scaffold was ~170-200 μm. However, attempting to fabricate a lattice scaffold with a comparable wall thickness led to severe printability and structural stability limitations: the printed walls deformed markedly and the construct collapsed during handling (see figure below). Because a key objective of this work was to promote rapid vascularisation *in vivo*, where sufficient mechanical integrity is required to provide a supportive environment after implantation, we increased the lattice wall thickness to approximately 350 μm to achieve comparable modulus while maintaining structural stability.

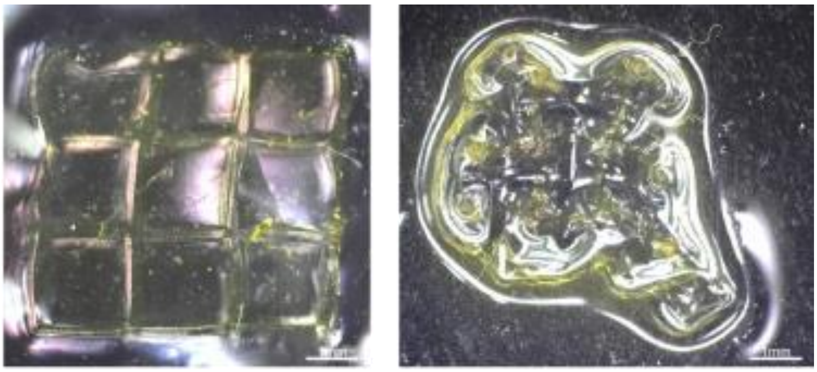

In addition, lattice scaffold is actually composed of multiple plates. To confirm

that the functional discrepancy was attributable to the 3D curvature, we designed planar plate and gyroid structures with comparable wall thickness and directly compared their vascularisation potential, provided with molecular evidence (Fig. 3 in the revised manuscript).

For clarity, the detailed design process for lattice has been added to Supplementary Note 2 in Supplementary Information:

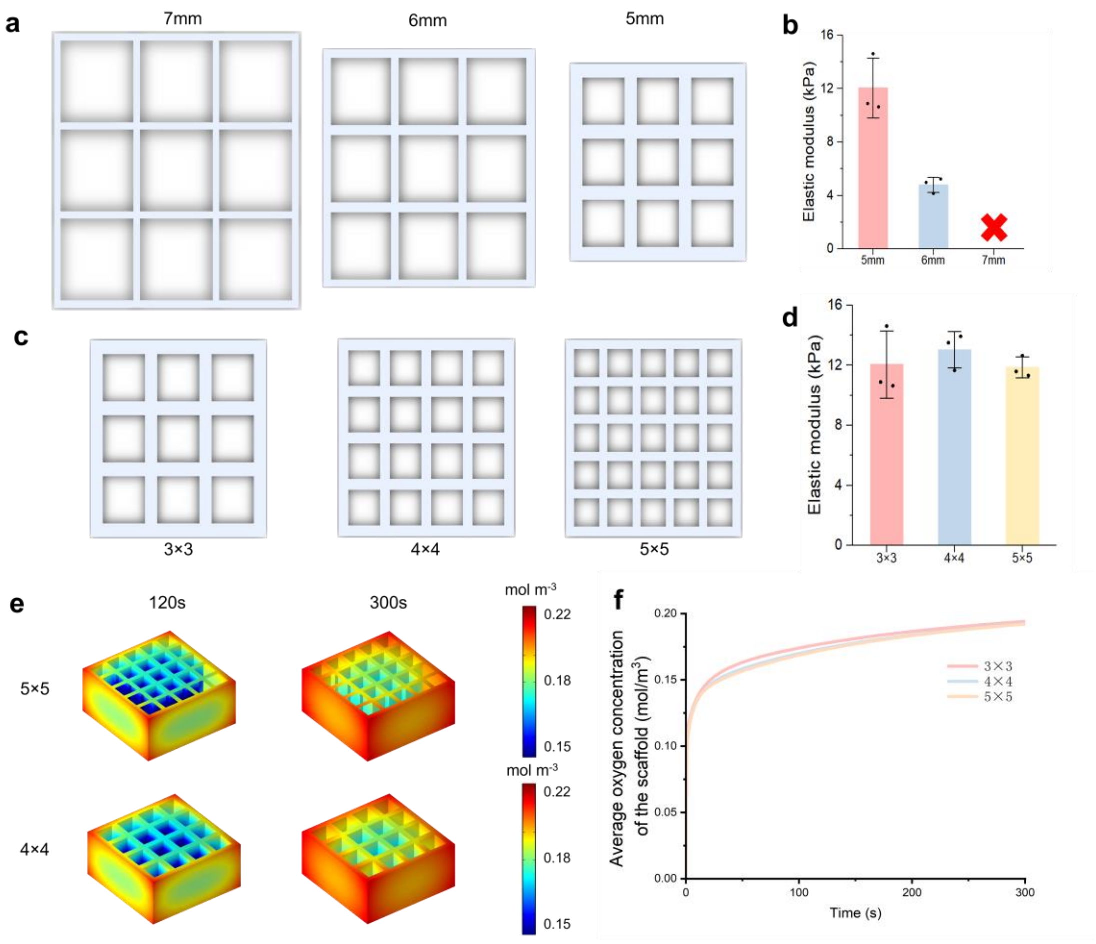

Note that the data has been added in the Supplementary Information.

References:

1. Ouyang L, Rao R, Zhao Y, Sun W, Effect of bioink properties on printability and cell viability for 3D bioplotting of embryonic stem cells. Biofabrication, 2016, 8:035020.

C5: Clarifying Geometry Effects in Figure 3: Figure 3 employs four different geometries to study curvature effects; however, variations in other geometric features (e.g., symmetry, surface connectivity) may confound interpretation. A more rigorous design—e.g., sequential curvature variation within a single base geometry—could provide clearer insights into curvature-induced endothelial responses.

Response: Thank you for this valuable comment. For the effect of curvature variation, we have conducted tube formation assay for three gyroid structures with varying mean Gaussian curvatures (-5.00, -2.30 and -1.25 mm^{-2}), representing high, moderate and low curvature magnitudes, respectively. The corresponding cell morphology and quantitative analyses of capillary network parameters are shown in the figure below, and the scaffold parameters are summarised in the table below. The relative density was kept constant at 20% to ensure comparable oxygen diffusion capacity and total cell number among the groups. Compared with the scaffold with lower curvature magnitude, cells cultured in the scaffold with moderate curvature exhibited a more elongated morphology and significantly improved capillary network parameters (number of junctions, number of segment and total network length), indicating enhanced endothelial network formation. However, this promotive effect diminished at the highest curvature magnitude, likely due to increased local geometric confinement. Curvature-dependent changes in focal adhesion organisation, actin stress fibre formation, and traction force have been reported in curved and spatially confined microenvironments, suggesting that excessive curvature may restrict the cell spreading and force transmission required for efficient cell migration and network formation (1-2). The impact of curvature on endothelial alignment was somewhat minor.

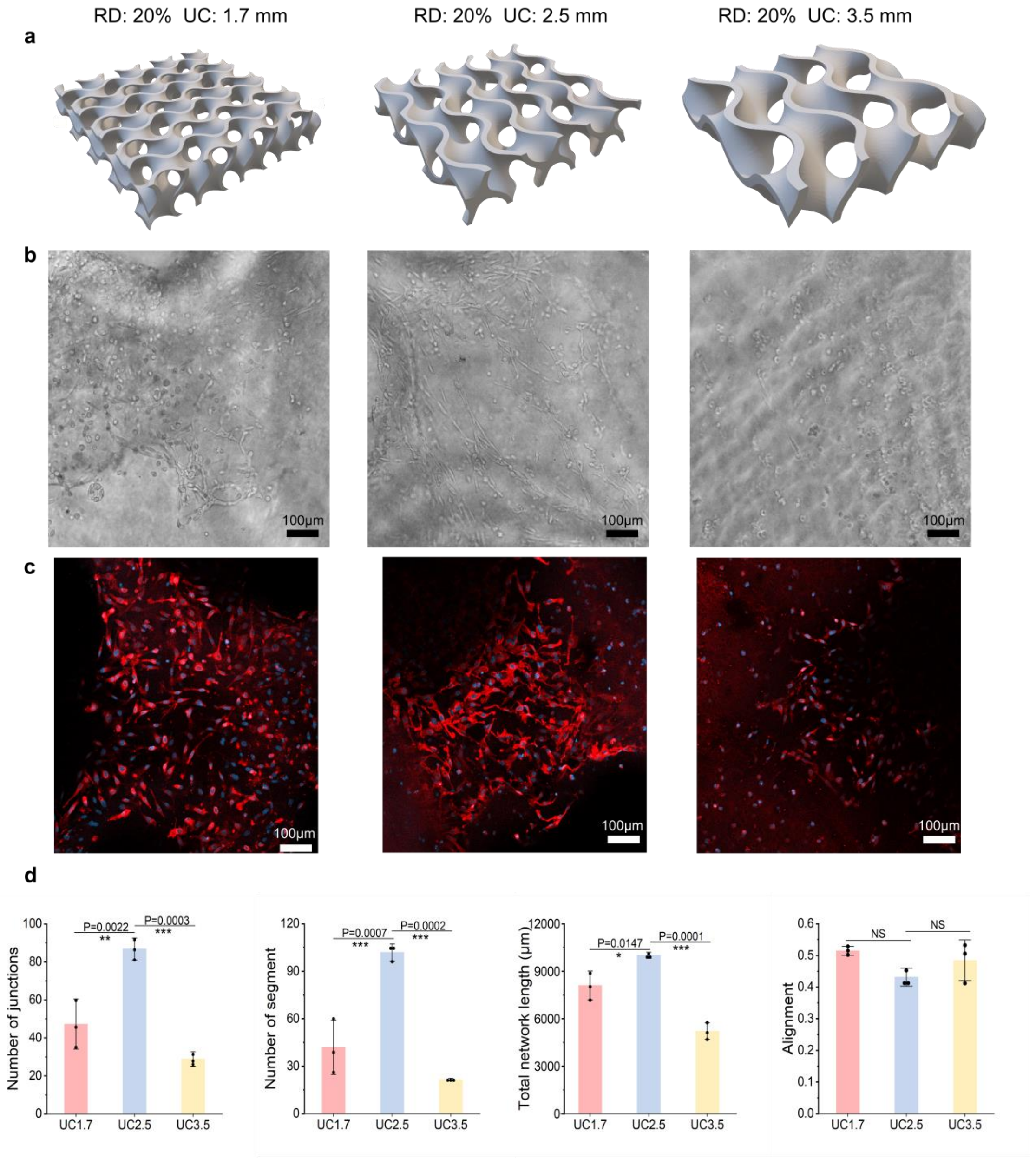

Size (mm) L×W×H	Unit cell (mm)	Relative density (%)	Mean Gaussian curvature (mm^{-2})
7×7×1.5	1.7	20%	-5.00
	2.5		-2.30
	3.5		-1.25

Note that the figure and table have been added to Supplementary Fig. 3 and Supplementary Table 1, respectively, in the Supplementary Information.

The above results motivated us to further investigate the molecular

mechanisms underlying the curvature effects. Therefore, we have revised Fig. 3 accordingly. Because the present study focuses on the gyroid scaffold with hyperboloidal curvature, and all major *in vitro* and *in vivo* results were derived from this geometry, we removed the data related to cylindrical and spherical curvatures for improved clarity and consistency. We instead added new results showing molecular evidence of curvature-induced endothelial responses and the influence of curvature variation on capillary formation. Specifically, we measured the biophysical cue-related proteins: integrin β1, YAP, and p-YAP (see the figure below). The gyroid scaffold increased integrin β1 expression, consistent with enhanced actin cytoskeleton organisation and filopodia formation. Moreover, nuclear YAP was increased and YAP phosphorylation was decreased, indicating enhanced YAP nuclear translocation in response to the 3D curved architecture.

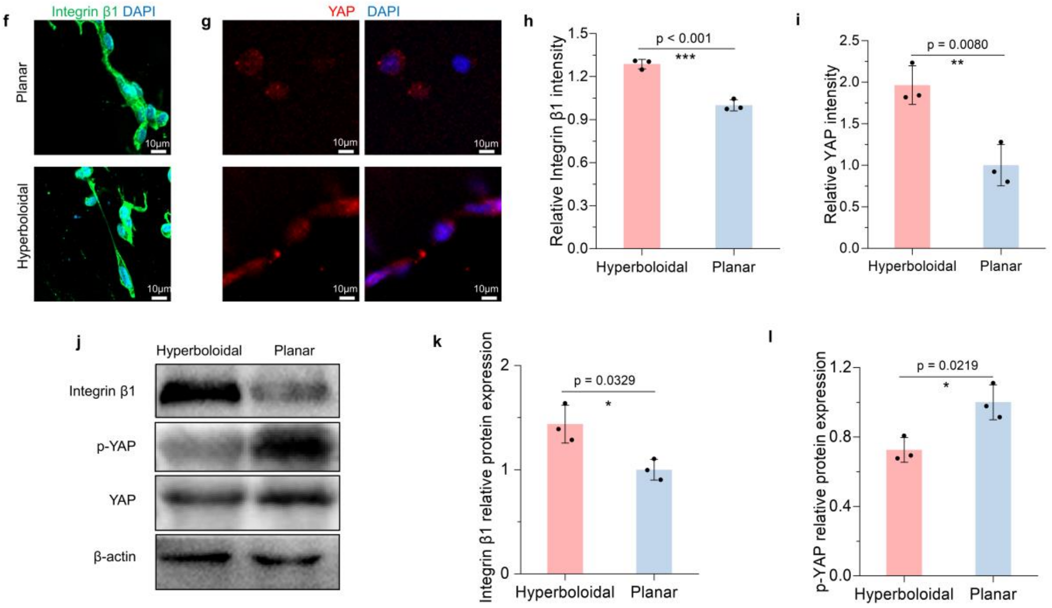

Note that the data has been added to Fig. 3.

References:

1. Dessalles CA, Leclech C, Castagnino A, Barakat AI. Integration of substrate- and flow-derived stresses in endothelial cell mechanobiology. Commun Biol. 2021 Jun 21;4(1):764.

2. Wenli Jiang, Xinghong Yao, Jian Zhong, Zhi Ouyang, Junyi Shen, Yan Qiu, Ye Zeng, Spatial confinement modulates endothelial cell behavior and traction force in 3D hydrogel microgrooves, Materials Today Bio, Volume 26, 2024,101074.

C6: Quantification of Endothelial Alignment: The manuscript reports improved endothelial alignment within gyroid scaffolds based on qualitative imaging. However, quantitative metrics such as alignment index or orientation histograms are not provided. Including such analyses would provide stronger support for curvature-induced alignment.

Response: We have added a quantitative alignment parameter to the analysis of capillary network formation (see the figure below). Endothelial alignment is known to be an adaptive response to mechanical stimulation, allowing cells to reduce the mechanical stress acting on them (1). In our study, endothelial alignment in the gyroid scaffold increased by approximately 2 times, suggesting that the curved architecture may impose mechanical cues on the cells and thereby act as a potential initiator of mechanotransductive signalling.

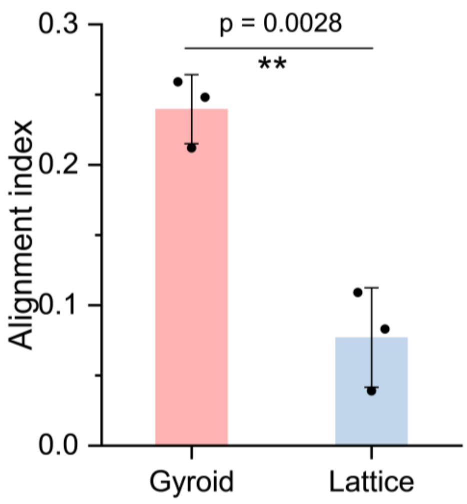

Note that the figure has been added in the Supplementary Information.

Reference:

1. Sinha, R, Le Gac, S, Verdonschot, N, Berg A, Koopman B, Rouwkema J, Endothelial cell alignment as a result of anisotropic strain and flow induced shear stress combinations. Sci Rep, 2016, 6:29510.

C7: Clarifying Spheroid Interpretation in Figure 5a: The cellular clusters observed in gyroid scaffolds are described as spheroids, but fluorescence imaging alone does not distinguish between multicellular aggregates and enlarged single cells. Incorporating nuclear staining (e.g., DAPI) would improve interpretation and confirm spheroid formation.

Response: Thank you for this clarification. We acknowledge that Fig. 5a, which presents only HepG2 staining, is not sufficient on its own to confirm spheroid formation. Rather, this panel was included to demonstrate the difference in tumour cell distribution between the two scaffold types after 14 days of culture. The formation of multicellular aggregates is more clearly shown in Fig. 5c, where HUVECs, HepG2 cells, and nuclei are stained separately in different colours. This point has now been clarified in the revised manuscript.

C8: Tumor Volume Data and Visual Evidence in Figure 6b: While tumor growth differences between scaffold types are quantitatively shown, representative images of explants would enhance interpretation. Including visual documentation alongside growth curves would provide more immediate context, as recommended in prior vascularization studies. Suggested references: Gao et al., Advanced Functional Materials (2017); Shukla et al., International Journal of Bioprinting (2024).

Response: Thank you for this useful suggestion. The purpose of the *in vivo* study was to assess whether the scaffolds could support the development of mature tumour tissue. Accordingly, the explants were collected at 24 days post-implantation, at which point the tissues had reached a mature stage suitable for evaluating morphology, composition, and vascularisation. These analyses provided clear evidence of the differences between the gyroid and lattice scaffolds.

We agree that additional intermediate time points could offer further information on tumour growth dynamics. However, such an approach would require a substantial increase in the number of animals, as independent cohorts would need to be sacrificed at each time point. In view of the principles of ethical animal use and the increasingly stringent standards of ethical approval, we considered this approach difficult to justify. We therefore focused on the terminal endpoint, which allowed us to obtain the most relevant biological information while minimising animal use.

C9: Figure Panel Labeling: In Figure 1, the panel labels (e.g., f–g) are not in sequential order, which may hinder reader comprehension. Consider reordering the panels or adjusting the labeling for improved clarity.

Response: Thank you for this helpful suggestion. We have rearranged the order of the sub-figures to ensure they are presented in a clear and sequential manner.

C10: Comparative Design Summary: A brief comparison of the gyroid and lattice scaffold systems—including aspects like fabrication complexity, resolution, and biological outcomes—would clarify the rationale for selecting gyroid architecture. A summary table or concise comparison would aid reader understanding.

Response: We agree that a direct comparison between the gyroid and lattice scaffold systems would help readers more clearly understand the rationale for selecting the gyroid architecture. We have therefore added a concise comparative summary in the revised manuscript (a summary table), covering fabrication complexity, printing resolution, structural and mechanical characteristics, and biological outcomes. This addition more clearly illustrates the distinct advantages of the gyroid scaffold over the lattice design in the context of soft, cell-laden tissue engineering.

The following content and table have been added to the revised manuscript and Supplementary Information, respectively:

"Compared with the lattice scaffold, the gyroid scaffold exhibited superior structural continuity, higher printing fidelity and stability at comparable RD, and enhanced biological performance, including improved endothelial morphogenesis, vascular network formation, and tumour tissue development."

Table 2 Comparison between lattice and gyroid scaffolds

Feature	Lattice scaffold	Gyroid scaffold
Geometry	Planar strut-based	Continuous TPMS / hyperboloidal
Fabrication stability	Lower at thin wall thickness	Higher structural stability
Printing fidelity	More prone to collapse/deformation	Better fidelity in soft GelMA
Curvature cue	Limited / planar	Continuous 3D curvature
Endothelial response	Weaker morphogenesis/networking	Enhanced filopodia and network formation
In vivo outcome	Lower tumour maturation/vascularisation	Improved tumour tissue formation/vascularisation

Note that the table has been added in Supplementary Information.

C11: Marker Labels in Figure 5a: Adding staining marker names directly onto the fluorescence images in Figure 5a would facilitate easier interpretation.

Response: Thank you for clarifying this point. Figure 5a shows only the staining of HepG2 cells, and we have added the corresponding label to the figure for clarity.

C12: Terminology Consistency: Terms such as "gyroid scaffold" and "gyroid structure" are used interchangeably across the manuscript. Consistent terminology throughout the text, figures, and captions is recommended.

Response: We have carefully reviewed the manuscript and standardised the terminology throughout the text, figures, and captions. Specifically, “gyroid scaffold” has now been used consistently to avoid confusion.

Comments from reviewer #4 and replies:

We sincerely thank the reviewer for the thoughtful, detailed, and constructive comments. These suggestions have significantly improved the quality, clarity, and overall presentation of the manuscript. We have carefully revised the manuscript accordingly, and our detailed, point-by-point responses are provided below.

C1: English needs to be edited: long sentences, not always with proper grammar. Typos: Line 71: (aka cell printing), please avoid colloquialisms. Uniform the use of italics throughout the text (e.g. in vivo and in vitro).

Response: Thank you for this helpful comment. We have carefully revised the manuscript to improve the English language, sentence structure, and grammar throughout. In particular, overly long sentences have been shortened or rewritten for clarity, and typographical and grammatical errors have been corrected. We have also removed colloquial expressions, including “aka cell printing”, and replaced them with more formal scientific wording. In addition, the use of italics has been standardised throughout the manuscript, including terms such as *in vivo* and *in vitro*.

C2: Abstract: Recapitulate the study but could be more precise

Response: Thank you for this valuable comment. We agree that the Abstract can be made more precise. Accordingly, we have revised the Abstract to enhance its clarity and specificity, providing a more concise summary of the study objectives, methodology, key results, and significance.

C3: Introduction: Introduction well structured that provides the required information, context and rationale but that is a little too long. It would be sharper and more straight to the point if it would be condensed a little. It is a detail but I wouldn't mention experimental details like cell number in the introduction. The last paragraph seems more like an abstract with appreciative words than an introduction (for example, successfully, improved, etc.).

Response: Thank you for this helpful comment. We agree that the Introduction was somewhat too long and included details that were more appropriate for later sections of the manuscript. In the revised version, we have condensed and streamlined the Introduction to make it more direct and focused. Experimental details, such as cell number, have been removed from this section and retained only where they are more appropriately presented. We have also revised the final paragraph of the Introduction to avoid abstract-like language and appreciative wording such as "successfully" and "improved", so that it now better reflects the purpose and scope of an Introduction.

C4: A comment about the possible structures in the organism that are indeed gyroids is advised to highlight the interest of changing from a lattice structure to the one proposed. It is not clear why a cancerous cell line (HepG2) has been used in the context of the study (Tissue engineering) and why tumor formation is considered a success by the authors. I would rather use more relevant cell types (i.e. iPSC, hepatocyte progenitors, MSC) and focus on cell functionality as it is the key parameter for the proof-of-concept. If not, I would rather present the construct as a tumor model rather than an engineered tissue.

Response: Thank you for these valuable comments. We agree that both points required further clarification in the manuscript.

First, in native vasculature, ECs are exposed to curvature cues of various geometries, including the cylindrical curvature of blood vessels and the hyperbolic curvature at vascular bifurcations, with curvature magnitudes ranging from 0.1 to 200 mm^{-1}. The gyroid architecture used in this study possesses tunable curvatures that fall within this physiologically relevant range. To further demonstrate its biological relevance, we conducted additional experiments to evaluate the effect of curvature magnitude on tube formation (Supplementary Fig. 3) and found that moderate curvature promoted the self-assembly of EC-mediated vascular networks. In addition, at the molecular level, we examined mechanotransduction-related proteins, including integrin β1, Yes-associated protein (YAP), and phosphorylated YAP (p-YAP). Immunofluorescence staining and western blot analysis showed that, compared with the planar plate, the gyroid scaffold upregulated integrin β1 and YAP expression (Fig. 3). Together, these results support the biological relevance of the gyroid architecture and help explain the rationale for moving from a conventional lattice structure to the proposed gyroid scaffold, whose continuous surface, high interconnectivity, and spatially varying curvature are advantageous for mass transport, structural stability, and cell guidance in 3D environments.

Second, we appreciate the concern regarding the use of HepG2 cells and the interpretation of tumour formation. Our intention was not to present this construct as a regenerative liver substitute, but rather as a vascularised 3D liver tumour model for evaluating the scaffold's ability to support long-term multicellular culture, endothelial network formation, and *in vivo* tissue

development. We agree that, in this context, the term “tumour model” is more appropriate than “engineered tissue” in sections specifically involving HepG2-based constructs. We have therefore revised the manuscript accordingly to avoid ambiguity.

We also agree that more physiologically relevant cell sources, such as iPSC-derived cells, hepatocyte progenitors, or MSC-based systems, would be highly valuable for future studies aimed at regenerative applications. Cell sources such as iPSCs or MSCs, although highly relevant for regenerative applications, are not stable cell lines and often exhibit greater biological variability, which makes it more difficult to control experimental consistency. In the present work, however, HepG2 cells were selected because they provide a robust and reproducible model for proof-of-concept evaluation of scaffold-supported vascularisation, multicellular organisation, and tissue-related functionality. We have clarified this rationale in the revised manuscript and emphasised that the present study should be interpreted primarily as the development of a vascularised tumour model, while also demonstrating a scaffold design strategy that may be extended to other soft tissue engineering applications using other cell sources.

C5: Results and Discussion: The authors have elected to have one results and discussion part. But sometimes, the discussion should provide more links and positioning with and toward the existing literature. It would help to assess the impact of the work.

Response: Thank you for this insightful comment. For clarity, we have separated the original Results and Discussion section into two distinct sections in the revised manuscript. In addition, we have expanded the Discussion to include further comparisons with prior studies and more explicit positioning of our findings within the current state of the field. In particular, we have incorporated additional relevant references, clarified the key differences between our system and previously reported curvature-based scaffold designs, and highlighted how our results advance current understanding of scaffold architecture, mechanotransduction, and vascularisation in cell-laden systems. These revisions improve the contextualisation of our work and better emphasise its novelty and significance.

C6: Optimal design, Figure 1

Response: We have reshaped the interpretation logic for clarifying the design process of the gyroid scaffold (Fig. 1).

We first determined the concentration of the GelMA material. A 10 wt% GelMA formulation is widely used for engineering 3D tissue constructs because it provides a practical balance between structural integrity and biological compatibility. In addition, reported moduli for liver tumours are generally higher than those of normal liver (1-7 kPa), and span a broad range depending on disease stage. During progression from fibrosis to cirrhosis and ultimately hepatocellular carcinoma, stiffness can increase substantially; representative reported ranges include approximately 4-13 kPa for fibrosis, 9-25 kPa for cirrhosis, and 14-72 kPa for hepatocellular carcinoma. In our study, the measured material stiffness of 10 wt% GelMA was ~13.1 kPa (Supplementary Fig. 2), which falls within the lower end of tumour-associated stiffness ranges and provides a mechanically relevant matrix for liver cancer cell culture.

We then focused on the material exchange capacity because it is key to cell viability. We found that relative density (RD) was the dominant design parameter affecting diffusion, while the impact of unit cell (UC) was minor. RD was inversely proportional to matter diffusion, and smaller RD values (e.g. RD=10% or 20%) promoted diffusion significantly by offering higher surface specific area, thinner wall thickness and larger pore size.

We next added the constrain of structural stability. For soft hydrogel 3D printing, the conflict between structural stability and cell viability always exists. Theoretically, RD=10% offers the best material exchange performance and thus the highest cell viability. However, it gives rise to a gyroid with a extremely thin-walled structure with a wall thickness less than 90 μm. Significant deformation and distortion were observed after printing. So, RD=20% was adopted because it ensured adequate cell viability while maintaining structural stability.

We then considered the mechanical property of the overall structure that impacts the *in vivo* structural stability. When RD (20%) was fixed, by varying UC from 2.0 to 3.5 mm, the compressional modulus were approximately 10.8, 12.0, 8.7 and 9.9 kPa, respectively. UC = 2.5 mm offered the highest modulus, providing better supporting capability.

Although UC showed minimum impact on oxygen diffusion, it could alter the curvature of the scaffold, which might deliver different geometric stimulation to cells. We finally investigated the impact of UC on capillary network formation. Varying UC size (1.7, 2.5 and 3.5 mm) produced different mean Gaussian curvatures (-5.00, -2.30 and -1.25 mm^{-2}), corresponding to high, moderate and low curvature magnitudes, respectively. Compared with the scaffold with low and high curvature magnitudes, cells cultured in the scaffold with moderate curvature (-2.30 mm^{-2} with UC = 2.5 mm) exhibited a more elongated morphology and significantly improved capillary network parameters (Supplementary Fig. 3). To this, the geometric parameters (RD = 20% and UC = 2.5 mm) not only provided adequate diffusion capacity and mechanical stability, but also delivered stronger geometric stimulation for capillary formation. Therefore, this optimised architecture was used in the subsequent studies.

1) It is not clear how the different UC were selected, based on which rational and why the values are different between Fig1 and S1.

Response: The UC values in Fig1 and S1 are the same. For how the different UCs were selected, please refer to the comment C6 above.

2) When changing RD and UC, does this change the global amount of material in the scaffold or does it stay constant?

Response: RD (%) defines the volume fraction of the entity. Changing RD will alter the global amount of material.

3) What is the impact of RD and UC changes on the total porosity?

Response: The total porosity, as the complement of volume fraction, is determined solely by RD and therefore remains unchanged when RD is kept constant. In contrast, the size of UC is not directly related to the total porosity or volume fraction. When RD is fixed, increasing UC simultaneously increases the wall thickness (Fig. 1c) and the pore diameter (Supplementary Fig. 1), while reducing the number of pores. As a result, the overall porosity remains constant.

4) If the scaffold is 1.5 mm high, it is not very thick. So, the diffusion of oxygen and nutriment is not as much a challenge as when the scaffold is bigger. The justification (ethical requirement) is not very clear, especially when it is not very complicated to implant bigger structures subcutaneously in mice.

Response: Thank you for this important comment. Oxygen transport in scaffold-based constructs is generally limited to approximately 100-200 μm, such that cells located farther from a perfused boundary are at risk of hypoxia; accordingly, constructs thicker than about 400 μm are prone to developing hypoxic regions (Ref1). In this context, a scaffold height of 1.5 mm is still relatively large for a cell-laden construct, compared with many previous studies that reported thicknesses of <1 mm (Ref2-Ref5). Therefore, although the present scaffold does not yet represent a very highly scaled tissue model, its height, together with the high cell density used in this study, provides a sufficiently diffusion-constrained system to evaluate whether the gyroid architecture can support uniform vascular network formation and multicellular function throughout a fully 3D cell-laden construct.

In addition, the selected height was also influenced by the layer-by-layer fabrication mode of DLP bioprinting. Increasing scaffold height requires longer cumulative light exposure, which prolongs cell exposure to free radicals generated during photocrosslinking and may compromise cell viability through damage to cell membranes and proteins. A height of 1.5 mm therefore allowed us to balance construct thickness with printing fidelity and cytocompatibility. We have revised the manuscript to clarify this rationale and to avoid implying that the current size was constrained primarily by ethical considerations.

Ref1: J. Rouwkema, N. Rivron, C. van Blitterswijk, Vascularization in tissue engineering, Trends in Biotechnology, 26(8):434-441 (2008).

Ref2: J. W. Lee, Y. J. Choi, W. J. Yong, F. Pati, J. H. Shim, K. S. Kang, I. H. Kang, J. Park, D. W. Cho, Development of a 3D cell printed construct considering angiogenesis for liver tissue engineering. Biofabrication 8, 015007 (2016).

Ref3: F. Maiullari, M. Costantini, M. Milan, V. Pace, M. Chirivì, S. Maiullari, A. Rainer, D. Baci, H. E. Marei, D. Seliktar, C. Gargioli, C. Bearzi, R. Rizzi, A multi-cellular 3D bioprinting approach for vascularized heart tissue engineering based on HUVECs and iPSC-derived cardiomyocytes. Scientific Reports 8, 13532 (2018).

Ref4: S. Lee, E. S. Sani, A. R. Spencer, Y. Guan, A. S. Weiss, N. Annabi, Human-Recombinant-Elastin-Based Bioinks for 3D Bioprinting of Vascularized Soft Tissues. Advanced Materials 32, e2003915 (2020).

Ref5: W. Zhu, X. Qu, J. Zhu, X. Y. Ma, S. Patel, J. Liu, P. R. Wang, C. S. E. Lai, M. L. Gou, Y. Xu, K. Zhang, S. C. Chen, Direct 3D bioprinting of prevascularized

tissue constructs with complex microarchitecture. Biomaterials 124, 106–115 (2017).

5) I don't understand the sentence starting in line 174 to 177. What is the relevance of the C2C12 cells here? The information is confusing as the authors reference the figure 1c and then describe results from another paper (Ref. 41). It can be misleading as the reader might think that that these cells were used in this study.

Response: Thank you for pointing out this issue. C2C12 cells were used in our previous study to validate a theoretical model that had been developed as a comprehensive multiphysics framework, integrating fluid dynamics, oxygen mass transport, cellular oxygen consumption, and cell growth kinetics to analyse cell behaviour within the scaffold. However, such a model is unnecessarily complex for the purpose of evaluating the diffusion capacity of the scaffold in the present study. For clarity and relevance, we have simplified the analysis to a pure diffusion model, which predicts the oxygen concentration distribution within cultured scaffolds with different geometric parameters. We believe this simplified model is sufficient to guide the selection of the optimal scaffold geometry in the current work.

6) Fig1e: I guess that you don't really need modelling to anticipate that a scaffold with the same mass of polymer (it seems the case from S1 even if the information is not provided) but with smaller pores and thicker walls will support a lower diffusion of oxygen and then will have a lower cell viability. I don't think that result required the model.

Response: Increasing the relative density (RD) increases the wall thickness (Fig. 1c) and reduces the pore size (Supplementary Fig. 1), both of which impair diffusion. These effects make diffusion monotonically decreasing with increased RD (Fig. 1g). So, the influence of RD on diffusion is relatively straightforward, making the selection of an optimal RD more direct. In contrast, increasing the unit-cell size (UC) enlarges the pores (Supplementary Fig. 1), which tends to enhance diffusion, but simultaneously increases the wall thickness (Fig. 1c), which can hinder diffusion by extending the diffusion path. Owing to these competing effects, the influence of UC on diffusion is less direct and cannot be determined intuitively. Therefore, modelling or experimental evaluation is required to identify the optimal UC.

7) Why pore size does not increase oxygen diffusion and thus concentration in the scaffold (Fig1f)? I am not convinced by the authors. For a same wall thickness, bigger pores should increase diffusion.

Response: In Fig. 1f (now Fig. 1g in the revised manuscript), the x-axis represents the unit-cell size (UC). As explained in Comment 7, increasing UC enlarges the pore size (Supplementary Fig. 1), which tends to improve diffusion, but simultaneously increases the wall thickness (Fig. 1c), which can hinder diffusion by lengthening the diffusion path. As a result, these two effects partially offset each other, leading to a relatively small overall impact of UC on diffusion. It should be noted that this balance may change for different material systems, depending on their intrinsic diffusion properties, which are generally characterised by the diffusion coefficient.

8) Fig1g and f: it is clear that the percentage of living cells decreased when the RD increased (quite logically), but what about the total number of living cells in the scaffold?

Response: This is an interesting question. The total number can be approximately calculated from the known parameters: cell density (1×10^7 cells ml^{-1}), the volume ($7\times7\times1.5\times20\%=14.7$ mm^3 for RD20 and $7\times7\times1.5\times50\%=36.75$ mm^3 for RD50), and cell viability (95% for RD20 and 84% for RD50 on day 7). So, the total cell number is 1.4×10^5 for RD20 and 3×10^5 for RD50, respectively. Indeed, RD50 contains a higher total number of live cells because of its larger volume fraction. However, the live cells are not uniformly distributed throughout the scaffold. Instead, cell survival is spatially heterogeneous, and dead cells are more likely to accumulate in regions farther from the channels, where mass transport is limited, thereby increasing the tendency for necrotic core formation. Importantly, As cell culture proceeds, hypoxia-induced cell death will become more pronounced (see Fig. 4).

9) The authors then state that since the cell viability is higher, RD20 was selected for further analysis but, one might consider it acceptable to lose some of the cells if the total number of living cells is higher, with RD50, especially since it is as low as 15%. But again, it will depend on whether the mass of polymer in the scaffold is the same or not and how many cells in total are incorporated in the scaffold. I would have balanced cell viability with mechanical properties to make that choice. It would then have been interesting to provide

the elastic moduli for the different RD and UC.

Response: This is an important point. In scaffold design, the relative importance of cell viability, mechanical performance, and total cell number depends on the intended application. For the present soft, cell-laden, prevascularised construct, we considered spatially uniform cell viability and sufficient mechanical stability to be the primary design criteria, while total cell number was treated as an important but secondary parameter. This is because a higher total cell number is not necessarily advantageous if it is accompanied by impaired mass transport, spatially heterogeneous viability, or local necrosis.

Local necrotic regions are generally undesirable in tissue-engineered constructs because they create a hostile microenvironment that compromises the overall success of the implant, primarily by inducing chronic inflammation, obstructing tissue regeneration, and risking structural failure. Unlike orderly apoptosis, necrotic cell death in the scaffold core, often caused by limited oxygen and nutrient diffusion, results in the release of cellular contents that trigger detrimental immune responses.

In this study, the choice of RD20 was made as a compromise between cell viability, spatially uniform cell survival, diffusion performance, and mechanical suitability for the current soft-tissue application, rather than on viability alone. We have reshaped the interpretation logic for clarifying the design process of the gyroid scaffold (Fig. 1), see comment 6).

10) Fig1j: the authors state that the scaffold RD20-2.5 mm has the same modulus than the liver at 12kDa, which is 2 to 4 time higher than the liver. Is it really correct then? While the impact on the cell. Viability can be minimized, what would be the impact of implanting a stiffer scaffold in the liver or what would be the impact on hepatic cell function?

Response: Thanks for clarifying this point. Regarding matrix stiffness, in the originally submitted manuscript we conflated normal liver tissue and liver tumour tissue, treating “liver tumour” as representative of liver tissue in general. Because our model uses HepG2 hepatocellular carcinoma cells, we now explicitly refer to the modulus of liver tumour tissue when discussing physiological relevance, and we have clarified this distinction in the revised manuscript. Matrix stiffness is an important biomechanical cue in the tumour

microenvironment. Increased stiffness can provide greater physical support and has been associated with altered cell adhesion, cytoskeletal organisation, and invasive/migratory behaviour (Ref1). Reported moduli for liver tumours are generally higher than those of normal liver (1-7 kPa), and span a broad range depending on disease stage. During progression from fibrosis to cirrhosis and ultimately hepatocellular carcinoma, stiffness can increase substantially; representative reported ranges include approximately 4-13 kPa for fibrosis, 9-25 kPa for cirrhosis, and 14-72 kPa for hepatocellular carcinoma (Ref1). In our study, the measured stiffness of 10 wt% GelMA was ~13.1 kPa (Supplementary Fig. 2), which falls within the lower end of tumour-associated stiffness ranges and provides a mechanically relevant matrix for liver cancer cell culture.

The second point clarified in the revised manuscript concerns the distinction between the different moduli reported in the study. Fig. 1j presents the compressive structural modulus, which was used to characterise the structural stability of the scaffold architecture. In contrast, the material modulus is generally measured using standard samples (e.g., cylindrical specimens) and is typically used to describe the intrinsic matrix stiffness of the biomaterial, particularly when assessing its biological relevance. In the present study, we also measured the stiffness of 10 wt% GelMA, which was approximately 13.1 kPa (Supplementary Fig. 2).

Ref1: Min K, Karuppannan SK, Tae G. The impact of matrix stiffness on hepatic cell function, liver fibrosis, and hepatocellular carcinoma-Based on quantitative data. Biophys Rev (Melville). 2024, 5(2):021306.

11) Fig1k: what is the pore size of the lattice? It looks like over 1 mm when it is smaller for the gyroid. I don’t think they could be compared then, especially as the wall thickness is different too. By increasing the number of pores, it should have been possible to get closer to the same wall thickness and pore size, shouldn’t it?

Response: Response: Unlike hard tissue scaffolds, the design space and manufacturability of hydrogel maaterials are significantly constrained due to their soften and highly dilute nature. In 3D bioprinting, printability, cell viability, and structural stability are the three core pillars of the “biofabrication window” (Ref1). Achieving success requires a delicate balance, as optimising one often compromises another.

In this study, the volume fraction, cell parameters (density and total number) and bulk mechanical properties of the lattice scaffold were adjusted to match with that of gyroid scaffold. The wall thickness for the gyroid scaffold was ~170-200 μm. However, attempting to fabricate a lattice scaffold with a comparable wall thickness led to severe printability and structural stability limitations: the printed walls deformed markedly and the construct collapsed during handling (see figure below). Because a key objective of this work was to promote rapid vascularisation *in vivo*, where sufficient mechanical integrity is required to provide a supportive environment after implantation, we increased the lattice wall thickness to approximately 350 μm to achieve comparable modulus while maintaining structural stability.

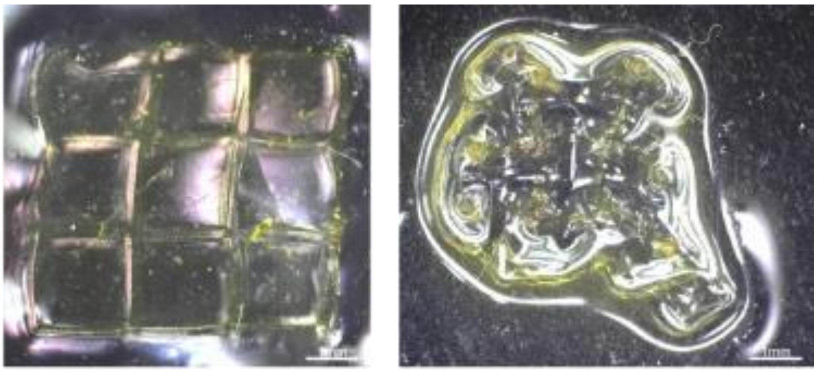

For clarity, the detailed design process for lattice has been added to Supplementary Note 2 in Supplementary Information.

Reference:

Ref1: Ouyang L, Rao R, Zhao Y, Sun W, Effect of bioink properties on printability and cell viability for 3D bioplotting of embryonic stem cells. Biofabrication, 2016, 8:035020.

12) Also, when I see the geometry of the lattice, I can not understand how oxygen diffusion can be reduced, especially it is the general concentration of oxygen that is measured. Wasn't it because there were more cells in the lattice? How many cells per scaffold were there?

Response: As mentioned earlier, the numerical model has been simplified to a diffusion-only model, and oxygen concentration was calculated using the built-in diffusion module in COMSOL. Fig. 1n presents the spatially averaged oxygen concentration for the lattice and gyroid scaffolds. During the diffusion process,

the oxygen concentration in the gyroid scaffold remained higher than that in the lattice scaffold until the system approached equilibrium.

If a cellular oxygen consumption term were introduced into the model, equilibrium within the scaffold would become more difficult to reach. In that case, the gyroid scaffold would be expected to maintain a higher oxygen concentration over a longer time scale, depending on the number and spatial distribution of viable cells.

The reduced diffusion observed in the lattice scaffold is mainly due to its greater wall thickness, which increases the effective diffusion path of oxygen molecules. We have explained this in the revised manuscript.

13) Lines 232-234; avoid introducing ahead “future” results.

Response: The “future results” from this section has been removed in the revised manuscript.

C7: Vascularisation, Figures 2 &3

1) I didn't understand why the authors stated that "to eliminate the impact of other supporting cells … ", lines 258-262. Wouldn't be more relevant to have pericytes also?

Response: Several approaches have been used to promote pre-vascularisation before implantation, including angiogenic growth factors, EC co-culture with stromal/perivascular support cells, immobilised bioactive factors, and scaffold-based strategies (Ref 1). In the present study, our aim was to determine whether the 3D gyroid architecture itself could facilitate rapid vascularisation and improve the viability and function of co-cultured tissue cells *in vivo* by supporting the formation of preliminary vascular structures *in vitro*. We therefore used ECs alone during *in vitro* culture to avoid confounding effects from supportive cells, especially with respect to vessel maturation and stabilisation, so that observed differences could be attributed primarily to scaffold geometry.

Following implantation, maturation of EC-lined structures still occurs through recruitment of host support cells, provided that the construct offers a permissive microenvironment for host-graft integration (Ref 2). We found that the gyroid scaffold promoted capillary network formation *in vitro* by improving oxygen transport and providing a curvature-related mechanical cue. Consistent with this, implantation of the gyroid scaffold significantly enhanced tumour tissue formation and vascularisation (Fig. 6). No oedema, extracellular leakage, or disruption of the vascular architecture was observed during the *in vivo* study. This point has now been clarified in the revised manuscript.

References:

Ref1: Soraia V. Lopes, Maurice N. Collins, Rui L. Reis, Joaquim M. Oliveira, and Joana Silva-Correia, Vascularization Approaches in Tissue Engineering: Recent Developments on Evaluation Tests and Modulation. ACS Applied Bio Materials, 2021, 4 (4):2941-2956.

Ref2: Gimble JM, Marolt D, Oreffo ROC, Wolbank S, Redl H, (Eds.), Reference Series in Biomedical Engineering-Tissue Engineering and Regeneration. Cell Engineering and Regeneration. Springer Cham, 2020.

2) Lines 276-277; the authors state that an active perfusing culture is difficult to translate to in vivo applications. Wouldn't in vivo have an active

diffusion of nutrients in a physiological way? How can a static culture be more easily translated to the in vivo state?

Response: Thank you for this important comment. We agree that the original wording was unclear. Our point was that active *in vivo* perfusion systems often involve increased technical complexity and may expose the scaffold to uncontrolled mechanical shear stress, which can potentially damage soft constructs (Ref 1). Therefore, if the implanted scaffold can form vascularised tumour tissue without relying on an external perfusion system, the overall preparation process can be substantially simplified. We have clarified this point in the revised manuscript.

Ref1: Zhu A, Reid E, Jain T, Mir A, Siddiqi U, Dunne O, Hibino N. Advancing Tissue Engineering Through a Portable Perfusion and Incubation System. Bioengineering (Basel). 2025, 12(5):554.

3) I don't understand the sentence from line 288-289. How the authors made this conclusion based only on Figure 2c, which is just a live and dead assay? Same with the following sentence (lines 289-292): first, there is no proof here that cell death is due to hypoxia and second, the images presented here do not support the conclusion as it is impossible to see a red signal. But I agree that in figure 2d then, the hypoxia seemed more pronounced. This part probably needs some editing in the way it is written.

Response: We thank the reviewer for this important comment. We agree that our original wording overstated the conclusion that could be drawn from Figure 2c. Accordingly, we have revised this section to avoid implying a causal relationship between cell death and hypoxia based solely on Figure 2c, and we have rewritten the corresponding sentences for greater clarity and accuracy.

4) Again, I don't think Figure 2c supports the claim made lines 295-299.

Response: Similar to comment 3), we have rewritten the corresponding sentences for greater clarity.

5) Figure 3: why in figure 3f the data are the result of n=30 samples and in Figures 3g and h, the data are the result of n=3 experiments? It would be better to homogenize the way the data are presented.

Response: Thank you for highlighting this issue. We agree that the different

definitions of n should be clarified. Figure 3f (now Fig. 3d) shows single-cell aspect ratio data; thus, 10 cells were randomly selected from each of 3 scaffold samples, resulting in n = 30 cells in total. This approach was used to provide a more representative and statistically robust evaluation of cell morphology. By contrast, Figures 3g and h (now Supplementary Fig. 11 and Fig. 3e) are based on measurements obtained from stained images of 3 independent scaffold samples, and are therefore reported as n = 3. We have revised the figure legends and main text to make these definitions explicit and improve clarity.

6) Line 325: Is it enhanced vascularization or is it reduced in the lattice condition? Do the authors have a control sample of a typical angiogenesis assay such as collagen I or even GelMA to evaluate the HUVEC intrinsic potential of vascularization? As the culture conditions are not specific for HUVECs and the passage (which is key for maintaining their vascularization potential) is not disclosed it would be interesting to have a positive control.

Response: Thank you for this valuable comment. We have clarified in the revised manuscript that the increased endothelial network-related parameters are indicative of enhanced vascularisation.

With respect to the positive control, GelMA is a photocrosslinkable liquid bioink, and in the absence of crosslinking it does not provide a structurally stable 3D environment for endothelial tube formation. As a result, culture in non-crosslinked GelMA is more comparable to a conventional 2D condition. Under these circumstances, HUVECs remain viable and proliferative, but capillary-like network formation is not observed, as illustrated in the figure below.

Regarding the passage number, HUVECs between passages 3 and 8 were used throughout the study in order to maintain high viability and preserve endothelial marker expression. This information has been added to Materials and Methods.

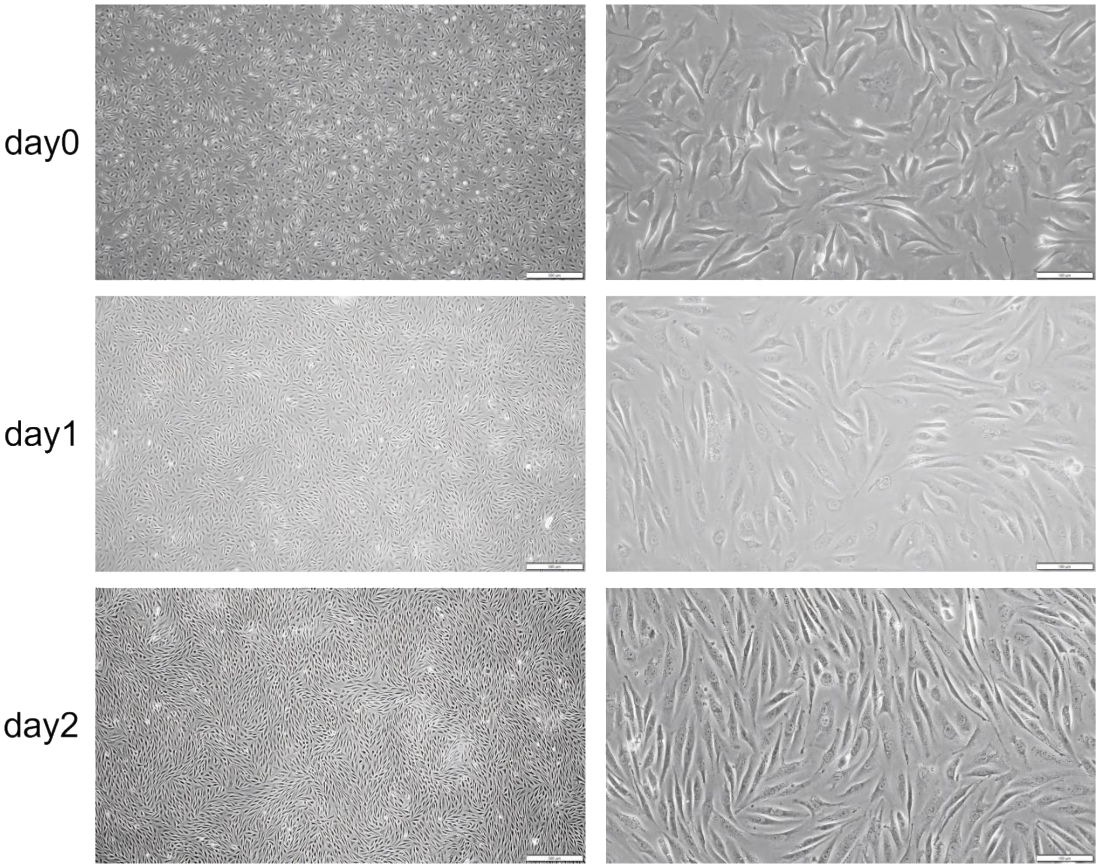
day0
day1
day2

C8: Generation of vascularized liver tissue

1) Figure 4c relates cell proliferation but the authors used a metabolic assay. So if no quantification of cell number (via DNA or protein quantification for instance) has been done, the authors should rather use “cell metabolic activity” rather than “cell proliferation”. Indeed, cells number could be the same with a higher metabolic activity in the gyroid compared to the lattice, or cell number could be lower in the lattice but with a higher activity than in the gyroid (as it could be the case, since viability is lower but the metabolic activity is still stable over time in the lattice). There is by the way no discussion making a link between data in figure 4b and 4c while both parameters are linked.

Response: Thank you for this insightful comment. We agree that the CCK-8 assay used in Figure 4c (now Supplementary Fig. 14 in the revised manuscript) reflects cell metabolic activity rather than direct cell proliferation, and that our original wording was therefore not sufficiently accurate. Accordingly, we have revised the text and figure labels throughout the manuscript to replace “cell proliferation” with “cell metabolic activity” where appropriate.

In addition, to directly assess cell proliferation, we performed genomic DNA quantification and included these data in the revised manuscript (Fig. 4c). The results showed that the cell number in the gyroid scaffold was approximately 1.28-fold higher than that in the lattice scaffold on day 14. Together with the viability results, these findings indicate that the enhanced metabolic activity observed in the gyroid scaffold can be attributed to both improved cell viability and increased cell proliferation.

2) Why in Figure 4d, Pimonidazole, is there no signal in the xy view while a signal is visible in the xz and yz views?

Response: Thank you for raising this point. We believe the reviewer is referring to the lack of an apparent signal in the zx view, while signals are observed in the xz and yz views. One possible explanation is that the zx plane is located closer to the scaffold surface, where nutrient and oxygen diffusion are more favourable. By contrast, the xz and yz planes extend into deeper regions of the scaffold, which are more susceptible to diffusion limitations and the development of hypoxia. This may explain why pimonidazole staining is less evident in the zx view.

3) Figure 4e: no “s” at the Hypoxic cells ratio.

Response: Thank you for identifying this error. We have corrected in the revised manuscript.

4) Figure 4h&i: before comparing the relative intensity of both cell types, did the authors check that the 2 cell types present the same intensity? Why they are no SD bars on the graph and no mention of the number of replicates.

Response: Thank you for this comment. The absolute fluorescence intensities of the two cell types are inherently different. In this analysis, our focus was not on directly comparing absolute fluorescence intensity between the two cell types, but rather on comparing the relative intensity of each cell type between the gyroid and lattice scaffolds. Therefore, the data were normalised to the fluorescence intensity measured on day 1 for each respective cell type, which is meaningful to reflect the increase rate of cell numbers. In addition, all images were acquired using identical staining and imaging settings to ensure comparability, and the quantification was performed using the same analysis workflow for all groups.

We agree that the absence of error bars and the lack of information regarding the number of replicates could lead to confusion. We have revised Fig. 4i to include SD error bars and have specified the number of biological replicates in the figure legend and main text. The data are now presented as mean ± SD from n = 3 independent samples.

5) Figure 4d: same comment: why no SD bars and no info on replicates?

Response: We apologise for the confusion. We believe the reviewer was referring to Fig. 5d rather than Fig. 4d. Fig. 5 focuses on hepatic function, and Fig. 5c was included only to illustrate the morphology of the co-cultured model. Because the image intensity in Fig. 5c does not directly reflect hepatic function, we have removed this subfigure from Fig. 5 in the revised manuscript. The assessment of hepatic function is instead supported by the protein measurements, western blot analysis, and qPCR results (Fig. 5d-g).

6) It is not clear for me why, as the title of this part specifically relates to “vascularized liver tissue construct with promoted hepatic function”, the authors seemed to relate the formation of tumor-like structures (and later on, of tumors in vivo) as a success or a desirable thing. I don’t believe tumor-like structures

are normal tissue constructs and are desirable in the scope of tissue engineering. I think the authors should reorient their paper toward a hepatic tumor model rather than normal hepatic tissue and tissue engineering.

Response: Thank you for this important comment. We agree that the wording in the original manuscript did not sufficiently distinguish between a tumour model and a normal hepatic tissue construct. Our goal in this study was to establish a vascularised 3D liver tumour model using HepG2 cells, in order to assess whether the gyroid scaffold could support multicellular organisation, endothelial network formation, and *in vivo* tissue development in a soft, cell-laden construct. Therefore, the formation of tumour-like structures *in vitro* and tumour growth *in vivo* were interpreted as successful establishment of the intended tumour model, rather than as evidence of normal liver tissue regeneration.

We agree with the reviewer that the manuscript should be oriented more clearly towards a hepatic tumour model. We have therefore revised the relevant sections of the manuscript to clarify this positioning and to avoid presenting the construct as a model of normal hepatic tissue in the context of regenerative tissue engineering.

7) On which basis the authors claim that increased expression of ALB and MRP2 is a sign of enhanced tissue function and maturation and not a sign of tumorigenicity?

Response: We agree that increased ALB and MRP2 expression should not be interpreted as definitive evidence of normal tissue maturation in a HepG2-based system. We have therefore revised the manuscript to avoid overstating this conclusion and now refer more cautiously to tumour formation with enhanced hepatic phenotype/function-related expression, rather than tissue maturation. We have also clarified throughout the manuscript that the construct represents a vascularised liver tumour model.

8) I don't understand the link between "few studies (line 546)… and … due to its known advantages in bone tissue engineering (line 548)".

Response: Thank you for pointing this out. We agree that the original wording was unclear and did not adequately express the intended logic. Our point was that gyroid scaffolds have been extensively explored in bone tissue engineering

because their architectural advantages, such as high interconnectivity, continuous curvature, and structural stability, are well suited to hard tissue applications. Because of these recognised advantages, a number of studies have attempted to translate gyroid architectures into cell-laden soft tissue engineering, where the requirements for material softness, printability, and long-term cell support are substantially different. We have revised this sentence in the manuscript to make this contrast clearer.

C9: Enhanced tumor tissue formation and neovascularization in vivo

1) Again, I don't see the link between tissue engineering, in vivo functionality, engineered tissue constructs, and tumor formation. Figure 6 legend states 'in vivo functions of the engineered tumor tissue constructs" but the data relates only to vascularization and not function.

Response: We agree that the original wording did not clearly distinguish between tumour model establishment, vascularisation, and tissue function, and therefore may have overstated the interpretation of Fig. 6. In the revised manuscript, we have therefore clarified that this part of the study concerns a vascularised 3D liver tumour model, rather than a normal regenerative tissue construct.

2) I don't think enhancing tumor growth is as difficult as producing a well-vascularized normal functional tissue, so the authors should tone down their statements, as it is a oversold as it is.

Response: we have toned down the relevant statements throughout the manuscript and revised the wording to avoid overinterpretation. In particular, we now position the work more clearly as the development of a vascularised 3D liver tumour model, rather than as a demonstration of functional normal tissue engineering.

3) Lines 612-615: here the authors mentioned tumor models, which indeed is more in line with the data. They should adapt the whole paper to this goal rather than tissue engineering.

Response: Please refer to the comment of 3) above.

4) As a general comment on these results; cancer tumor models (such as matrigel based) allow the incorporation of cells from the animal (such as Tumor associated macrophages (TAM) or cancer associated fibroblasts (CAFs)) for the formation of the tumor. How is this 3D structure comparable? Is there formation of tissue in the pore areas of the material that were empty? Could the authors analyze the other cells forming the tumor besides the HepG2 and HUVECs to explain the differential in growth between conditions?

Response: Regarding other cells besides the HepG2 and HUVECs, this is indeed a good question. However, it is out scope of this paper and will be

present in our another study. This study focuses the architectural advantages of the gyroid scaffold on vascularisation both *in vitro* and *in vivo*, rather than replicateing the full function of liver tumour.

In vivo, cells and tissue fill the hole of a scaffold through a complex process of cellular infiltration, migration, proliferation, and angiogenesis, guided by the scaffold's porous architecture and material properties (Ref1). The body uses the scaffold as a temporary 3D guide, replacing it with natural extracellular matrix as the scaffold degrades. So, the occupation of scaffold pores depend on cellular activities and the size of pores. From the immunohistochemistry and immunofluorescence images (Supplementary Fig. 17 and Fig. 6f), minimal empty space was observed in tumour tissue derived from the gyroid scaffold, whereas substantial void regions were present in tissue from the lattice scaffold, likely due to its larger pore size and reduced cellular activity.

Ref1: Chamorro CI, Zeiai S, Juul N, Willacy O, Huo J, Hilborn J, Fossum M. Exploring the Concept of In Vivo Guided Tissue Engineering by a Single-Stage Surgical Procedure in a Rodent Model. Int J Mol Sci. 2022, 23(20):12703.

5) Fig. S8 shows histological images of the tumors, scales are missing. What is the aspect of the holes of the 3D structure? From the images, the lattice sample seems like there is no tissue in the porous structure.

Response: We have added the scale bars to the figure (now Supplementary Fig. 17). Regarding the holes, please refer to the second paragraph of comment 4) above.

6) Do the authors assess if the methacrylated gelatin was degraded in vivo? Do the authors expect the printed material to disappear at some point?

Response: GelMA (gelatin methacryloyl) hydrogel degrades primarily through enzymatic action, specifically by matrix metalloproteinases like collagenase, which cleave the peptide chains. Its degradation rate is highly variable (from days to months) and dependent on concentrations (5–20% w/v), degree of substitution, crosslinking density, cells encapsulated, and etc. A 10 wt% GelMA can be degradaded completely when immersed in the collagenase-contained PBS at 37°C (Ref1). Encapsulated cells secrete enzymes, accelerating degradation, while simultaneously laying down new extracellular matrix, which can paradoxically slow down enzyme diffusion and slow down overall

degradation over time (Ref2).

In this study, at 24 days post-implantation, no residual hydrogel was detected in the gyroid scaffold by immunohistochemical or immunofluorescence analysis. In contrast, residual hydrogel was observed in a lattice scaffold sample in which tumour formation did not occur.

Ref1: Shie MY, Lee JJ, Ho CC, Yen SY, Ng HY, Chen YW. Effects of Gelatin Methacrylate Bio-ink Concentration on Mechano-Physical Properties and Human Dermal Fibroblast Behavior. Polymers (Basel). 2020, 12(9):1930.

Ref2: Krishnamoorthy S, Noorani B, Xu C. Effects of Encapsulated Cells on the Physical-Mechanical Properties and Microstructure of Gelatin Methacrylate Hydrogels. Int J Mol Sci. 2019, 20(20):5061.

7) Do the authors expect the encapsulated cells to "escape" the scaffold in vivo?

Response: Encapsulated cells can escape a scaffold *in vivo* primarily through the biodegradation of the carrier material or by active migration through the scaffold’s porous structure into the surrounding host tissue.

C10: Materials and methods

1) The resources like the mathematical model and the .slt files should be available for reproducibility.

Response: Thank you for this helpful suggestion. To improve reproducibility, the STL files used in this study will be uploaded to an open-access repository during re-submission process. The numerical model used in this study was simplified to a diffusion-only model, with oxygen concentration calculated using the built-in COMSOL diffusion module. We have also expanded the description of the model configuration, assumptions, and implementation details in the Materials and Methods section to facilitate reproduction of the simulation by others.

2) Line 698: Indicate origin, passage and cell culture conditions as for HUVECs response is important. Add information on how the cells were incorporated, dissolved in medium/PBS? how much volume? Also why you didn't use a specific medium for HUVECs (line 750)?

Response: Cell origin, passage and cell culture conditions has been included in the Cell culture section in Materials and Methods. Specifically, HepG2 cells were obtained from Procell Life Science & Technology Co., Ltd, China (CL-0103), while HUVECs were sourced from the National Collection of Authenticated Cell Cultures, China (SCSP-5535). All cells were cultured and maintained in high-glucose Dulbecco's modified Eagle's medium (DMEM, Gibco), supplemented with 10% fetal bovine serum (FBS, Gibco) and 1% antibiotic solution (streptomycin, 100 μg mL^{-1}, and penicillin, 100 units mL^{-1}, Sigma-Aldrich Corp.). The culture medium was refreshed every two days. Cells were incubated in a humidified atmosphere at 37° C with 5% CO_2 and 95% air, and were passaged every 3 to 4 days. HUVECs between passages 3 and 8 were used throughout the study in order to maintain high viability and preserve endothelial marker expression. HepG2 between passages 7-18 were used to ensure high metabolic activity.

Regarding cell incorporation for printing, cells were suspended in the GelMA precursor solution rather than in PBS or culture medium alone. 2.5 mL^{-1} bioink was used for each printing, allowing to produce 6 scaffolds (3 gyroid scaffolds and 3 lattice scaffolds) per time.This has been clarified in the revised manuscript.

For culture medium, HUVECs can be cultured in a variety of cell culture media, including high-glucose Dulbecco's modified Eagle's medium (DMEM, Gibco) supplemented with 10% fetal bovine serum (FBS, Gibco), which was used in this study and others (Ref1). We agree that endothelial-specific media are typically preferred for HUVEC culture. However, in this study, a co-culture system was required, and a unified medium was selected to support both endothelial and tumour cells. This represents a necessary compromise commonly adopted in co-culture models. To promote tube formation during scaffold culture, VEGF was also added in the medium. HUVECs maintained viability and endothelial characteristics under these conditions.

Ref1: Yifan Li, Jiafeng Li, Shuai Jiang, Cheng Zhong, Chenchen Zhao, Yang Jiao, Jian Shen, Huaizhi Chen, Meihan Ye, Jiayu Zhou, Xianyan Yang, Zhongru Gou, Sanzhong Xu, Miaoda Shen, The design of strut/TPMS-based pore geometries in bioceramic scaffolds guiding osteogenesis and angiogenesis in bone regeneration, Materials Today Bio. 2023, 20:100667.

3) Line 707: explain how sterility was ensured during the printing process.

Response: Thank you for this comment. All printing procedures were performed under aseptic conditions in a cleanroom environment. Prior to printing, all equipment and materials were sterilised using ultraviolet (UV) irradiation and disinfected with 70% ethanol. Components were handled using sterile techniques wherever applicable. The printer was equipped with a protective chamber in which photo-crosslinking occurred, minimising exposure to the external environment. All handling steps were conducted in a controlled manner to maintain sterility. We have clarified these points in the revised manuscript.

4) Line 727: Indicate number of gels per test and if they were from the same batch

Response: The samples were fabricated from the same batch of bioink, and three for each structure were used to calculate the statistical results. Due to the size limit of the bioink container of the printer (2.5 mL^{-1}), maximum 6 samples were allowed to fabricate per printing. Therefore, the samples might be fabricated from different printing. To assess batch-to-batch reproducibility, three samples of the gyroid scaffold with a relative density of 20% and unit cell size of 2.5 mm were fabricated from independent experiments, including separate

bioink preparation and printing (Supplementary Fig. 19). Variability between different batches was minor. We have clarified these points in the revised manuscript.

5) Line 736: Did the authors tested hydrogels from different fabrication batches?

Response: Please refer to comment 4) above.

6) Cell culture: indicate cell passage used for both cell types.

Response: HUVECs between passages 3 and 8 were used throughout the study in order to maintain high viability and preserve endothelial marker expression. HepG2 between passages 7-18 were used to ensure high metabolic activity. We have added this information in the revised manuscript.

7) Cell membrane fluorescent staining: Add information about time of labelling and how the excess was removed.

Response: Working solutions of DiO and DiR were prepared in PBS separately. Each cell type was incubated for 20 to 30 minutes at 37° C, followed by three centrifuge-assisted washes in PBS to remove unbound dye. We have added this information in the revised manuscript.

8) Endothelial networks analysis: Add information in how many images per sample and areas quantified in total.

Response: For each scaffold type, three images from independent samples were analysed, and one region of interest per image was selected for statistical evaluation. We have added this information in the revised manuscript.

9) Statistical analysis: number of samples, replicas and independent experiments is too ambiguous. It should be clearly stated in the manuscript. To use a t-test, a normality test of the results should be run. Also, the authors use t-test when more than two conditions are compared, therefore an ANOVA should be used, either one way or two way, when different times are also included. Please redo all statistics considering all of this.

Response: Thank you for this valuable comment. We agree that the statistical analysis required clarification and improvement. In the revised manuscript, the definitions of n, biological replicates, and independent experiments have been

clearly specified in both the Methods section and figure legends.

We have re-evaluated all statistical analyses. As this study primarily focuses on differences between the gyroid and lattice scaffolds, all comparisons were performed between these two groups using two-tailed t-tests where appropriate. Prior to analysis, data normality was assessed using the Shapiro-Wilk test to ensure the validity of the t-test. For datasets that did not meet this assumption (Figs. 6g and 6j in this study), non-parametric analysis using the Mann-Whitney U test was applied. All analyses have been updated and clarified in the revised manuscript.

Responses to Reviewer' Comments

Ref. No.: NCOMMS-25-23043B

Title: 3D triply periodic minimal surface gyroid hydrogel scaffolds for soft tissue engineering

Authors: Xiaoxiao Han, Ning He, Dandan Yuan, Na Li, Wei Zhu, Xiaolong Zhu, Jing Li, Xun Yuan, Wenxin Wang, Xingshi Gu, Feng Chen, Wei Wang

Journal: Nature Communications

The authors are very grateful to the reviewer for his/her valuable comments. All the comments have been considered and addressed very carefully. The changes, where necessary, have also been incorporated into the revised manuscript/supplementary document and highlighted in yellow.

The comments and corresponding responses (highlighted in blue in this document) are listed point by point below.

Comments from reviewer #2 and replies:

C1: The Fig. 5c image clearly shows a CD31/Albumin/DAPI triple-channel overlay, with the DAPI channel visible in blue and labelled in the panel, which fully resolves the previous concern regarding the distinction between multicellular aggregates and enlarged single cells. However, the current Fig. 5c caption lists only "albumin (ALB) for HepG2 (green) and CD31 for HUVEC (red)" and does not mention the DAPI channel that is already present in the image. We would kindly ask the authors to update the Fig. 5c caption to include the DAPI channel and to note, in one short sentence, that the nuclear staining supports the multicellular nature of the aggregates. If a simple quantitative descriptor (for example the average number of nuclei per aggregate in representative regions) can be extracted from the existing confocal data, adding it would further solidify the spheroid interpretation; however, this is optional and need not require additional experiments. The Fig. 6f caption already lists CD31/AFP/DAPI explicitly and requires no change.

Response: Thank you for this useful comment. The DAPI channel has been specified in the caption of Fig. 5c. The following statement has been added to Results section: to further characterise cellular heterogeneity and spatial distribution, the scaffolds were co-immunostained for albumin to identify HepG2 cells (green), CD31 to identify HUVECs (red), and DAPI to label nuclei (blue), demonstrating the multicellular nature of the aggregates (Fig. 5c).

C2: In the first round, four references [1 – 4] were recommended as closely related prior work on curvature-driven vasculature, curvature/geometry-based cell-fate guidance, and in vivo vascularisation and hepatic tissue engineering documentation. As these references were not cited or discussed in the revised manuscript, we would kindly ask the authors to revisit them in the Discussion with one or two sentences each, briefly comparing matrix, fabrication strategy, geometric control, and biological readout relative to the present study. Reference [1] (Park et al., Nature Communications 2023) is most directly relevant to Comment 3, as it demonstrates how vascular geometry controls endothelial behaviour and cancer-cell dynamics; reference [2] (Werner et al., Advanced Science 2017) is highly relevant to Comment 4, as it isolates surface curvature as an independent geometric variable under parameter-matched conditions and links it to stem-cell migration and differentiation via attachment

morphology and nuclear deformation; reference [3] (Moon et al., Materials Today Bio 2025) is closely aligned with the present work and with Comment 8, as it reports gyroid-architecture thick vascularised hepatic constructs; and reference [4] (Shukla et al., International Journal of Bioprinting 2024) provides a broader vascularisation-strategy context for Comment 8. Including these together would place the gyroid-based liver tumour platform more clearly within the existing soft-tissue curvature and vascularisation literature and provide appropriate context for the biological claims made in the manuscript.

Response: Thank you for this important comment. The following discussion has been added to Discussion section:

"Recently, Moon et al. (57) bioprinted a thick gyroid construct laden with HepG2 cells, followed by coating the interconnected vascular channels with ECs. Compared with the lattice scaffold, the gyroid construct achieved a more uniform cell distribution and higher cell viability under perfusion conditions, owing to more homogeneous fluid flow and optimised surface shear stress. This finding further underscores the importance of scaffold structural optimisation."

"Park et al. showed that tumour cells located in regions of higher vascular curvature within 3D bioprinted multilayered cerebrovascular conduits could activate the vascular barrier and disrupt endothelial junctions through increased secretion of inflammatory cytokines, highlighting the biomechanical role of curvature in regulating local microenvironments (62)."

The following four references have been included in the revised manuscript:

1. M. Werner, S. B. Blanquer, S. P. Haimi, G. Korus, J. W. Dunlop, G. N. Duda, D. W. Grijpma, A. Petersen. Surface Curvature Differentially Regulates Stem Cell Migration and Differentiation via Altered Attachment Morphology and Nuclear Deformation. Adv Sci. 4(2):1600347 (2016).

2. W. Park, JS. Lee, G. Gao, B. S. Kim, DW. Cho, 3D bioprinted multilayered cerebrovascular conduits to study cancer extravasation mechanism related with vascular geometry. Nat Commun 14, 7696 (2023).

3. YW. Moon, T. Dobroski, K. Willson, JO. Jeong, C. Bishop, A. Atala, J. J. Yoo, S. J. Lee, 3D bioprinted thick hepatic constructs with vascular network as a physiologically relevant in vitro organ model, Materials Today Bio 32:101786, (2025).

4. A. K. Shukla, D. Lee, S. Yoon, M. Ahn, B. S. Kim, Vascularization strategies for human skin tissue engineering via 3D bioprinting. International Journal of Bioprinting 10(3):1727 (2024).
